# Vanillin-tethered quinazolin-2,4-dione analogues through five- and/or six-membered nitrogen-containing heterocycles as antibacterial agents: synthesis, biological evaluation and molecular docking study

**DOI:** 10.1039/d5ra08138f

**Published:** 2026-01-27

**Authors:** Aboubakr H. Abdelmonsef, Saleh M. Elnaby, Ahmed M. Mosallam, Huda R. M. Rashdan, Hesham M. Alsoghier, Mohamed A. Raslan

**Affiliations:** a Department of Chemistry, Faculty of Science, South Valley University 83523 Qena Egypt aboubakr.ahmed@sci.svu.edu.eg; b Chemistry of Natural and Microbial Products Department, Pharmaceutical and Drug Industries Research Institute, National Research Centre 33 El Buhouth St, Dokki Giza 12622 Egypt; c Department of Chemistry, Faculty of Science, Aswan University 81528 Aswan Egypt

## Abstract

The escalating threat of drug-resistant bacteria confirms the urgent need to develop and identify new and potent antibacterial inhibitors. In the present research study, the molecular hybridization strategy involved the synthesis of vanillin linked to a quinazolin-2,4-dione skeleton through five- and/or six-membered nitrogen-containing heterocycles such as pyrazole, isoxazole and/or pyrimidine 1–15 and testing them for their *in vitro* antibacterial studies. The vanillin-derived chalcone 1 was synthesized by treatment of 3-(4-acetyl-phenyl)-1*H*-quinazolin-2,4-dione with 4-hydroxy-3-methoxybenzaldehyde (vanillin) through a base-catalyzed Claisen–Schmidt condensation reaction, then used as a key precursor for synthesis of a series of 14 bioactive compounds 2–15 by treatment with nitrogen nucleophiles *via* Michael addition reaction. Subsequently, the newly prepared compounds were structurally confirmed by well-known spectroscopic techniques such as FT-IR, ^1^H-NMR, ^13^C-NMR, mass spectroscopy, and elemental analysis. All the molecules were evaluated *in vitro* for their antibacterial activities, showing moderate to good potency, with compounds 2, 5 and 11 having significant activity comparable to the standard drug ciprofloxacin. Moreover, molecular docking simulations were also conducted to investigate the possible interactions of the bioactive moieties of compounds, ciprofloxacin, co-crystalized ligand and two references with the target enzyme. The binding scores correlated with the biological activity of the compounds, with energy scores ranging from −9.9 to −7.4 kcal mol^−1^, comparable to ciprofloxacin. Interestingly, the molecules 2, 5 and 11 formed highly stable H-bond, pi-cation and pi–sigma interactions with the amino acid residues LYS487, SER401, THR352, GLN348, SER347, and SER303, which are playing an important role in ensuring efficient binding of the ligand with glucoseamine-6-phosphate (GlcN-6-P) synthase (PDBID׃1moq). The enhanced antibacterial activity was ascribed to the presence of electron donating-groups –OH, and –OCH_3_ attached to pyrazole and/or isoxazole moieties, respectively. To evaluate the structural confirmation and chemical reactivity behavior of the quinazolin-2,4-dione skeleton, semiempirical studies were achieved. Overall, the findings underscore the potential of vanillin linked to quinazolin-2,4-dione skeletons through five- and/or six-membered nitrogen-containing heterocycles, particularly 2, 5 and 11, as promising candidates for development of potent antibacterial inhibitors.

## Abstract 2

The escalating threat of drug-resistant bacteria confirms the urgent need to develop and identify new and potent antibacterial inhibitors.

## Graphical abstract

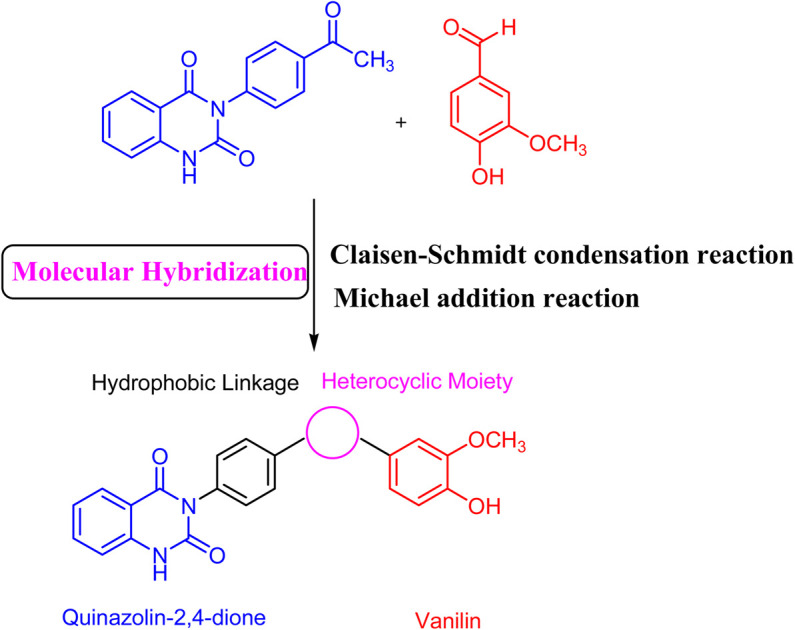

## 1. Introduction

The rise of antimicrobial resistance (AMR) poses a serious global health threat.^1–3^ By 2050, drug-resistant bacterial infections will be responsible for approximately 1.9 million deaths.^4,5^ Overcoming the resistance problem needs the development of new and potential molecules with novel mode of action. Therefore, synthesis of vanillin linked to quinazolin-2,4-dione skeletons through five- and/or six-membered nitrogen-containing heterocycles through a molecular hybridization strategy is disclosed in this regard.

Quinazolin-2,4-dione skeletons are versatile building blocks in pharmaceutical chemistry and drug discovery.^6–10^ They are some of the most prominent, widely used classes for preparation of various drugs with potential biological effects^11^ such as anticancer,^12^ anti-inflammatory,^13^ antimicrobial^14,15^ and anti-tuberculosis.^16^

Vanillin, a biologically active compound, has several pharmacological actions^17^ such as anti-inflammatory,^18^ anticancer,^19^ antidiabetic^20^ and antimicrobial effects.^21^ In addition, it is a promising building block for chemical and pharmaceutical industries.

Five- and/or six-membered nitrogen-containing heterocycles such as pyrazole, isoxazole and/or pyrimidine are fruitful sources of inspiration of medicinal and pharmaceutical chemists due to their spectrum of extensive biological and pharmaceutical potentials such as anticancer,^22^ anti-inflammatory,^23^ antiviral,^9,24,25^ antibacterial properties.^26,27^

**Fig. 1:**
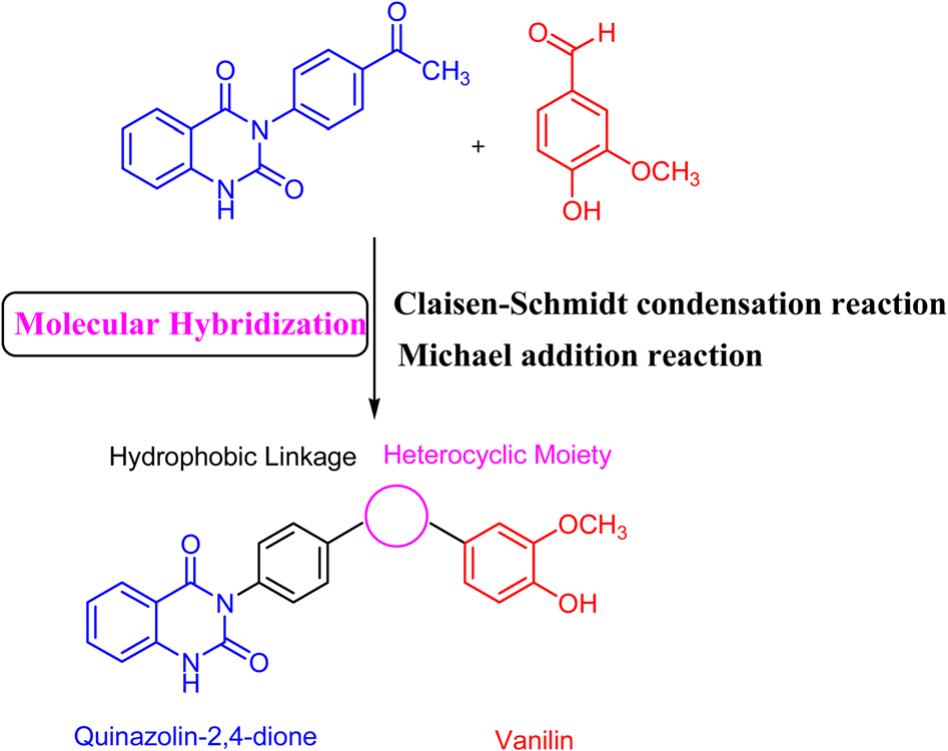
Design the concept of molecular hybridization of compounds incorporating vanillin linked to quinazolin-2,4-dione skeleton through five- and/or six-membered nitrogen-containing heterocycles.

**Fig. 2:**
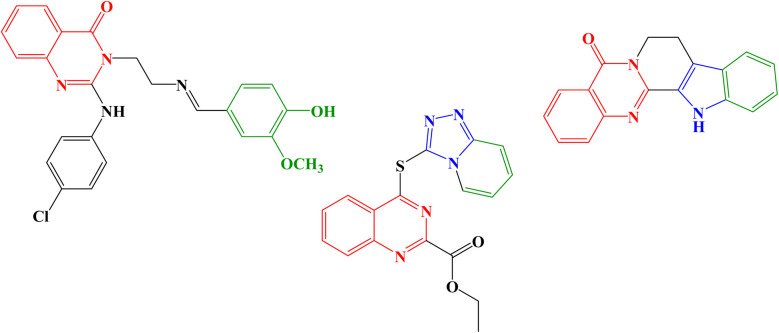
Antibacterial drugs with quinazoline structure, vanillin with five- and/or six-membered nitrogen-containing heterocycles.

Molecular hybridization strategy is a combination of different molecules to develop hybrid one with improved efficacy.^28^ The design strategy of newly prepared compounds 1–15 is declared in Fig. 1. Fig. 2 shows the approved marketed antibacterial drugs with quinazolin-2,4-dione structure, vanillin and five- and/or six-membered nitrogen-containing heterocycles.

Considering these results and our ongoing fascination with the synthesis of bioactive heterocycles,^29–35^ herein, the specific incorporation of vanillin *via* Claisen–Schmidt condensation and Michael addition to form particular series of fifteen quinazolin-2,4-diones was performed. Their chemical structures and purities were elucidated by spectroscopic and elemental analyses. In addition, their antibacterial efficacy was *in vitro* investigated against two Gram +ve strains, *i.e.* (*Bacillus subtilis* ATCC 6633 and *Staphylococcus aureus* NRRL B-767) and two Gram −ve strains, *i.e.* (*Escherichia coli* ATCC 25955, *Pseudomonas aeruginosa* ATCC 10145). Moreover, molecular docking studies of newly quinazolin-2,4-dione derivatives, ciprofloxacin, co-crystalized ligand and two references were conducted against glucoseamine-6-phosphate (GlcN-6-P) synthase, using PyRx virtual screening tool. Finally, their toxicity and pharmacokinetics were screened by the aim of AdmetSAR, and Molinspiration in order to determine the major scaffolds that possess promising antibacterial properties.

## 2. Results and discussion

### 2.1. Chemistry

**Scheme 1:**
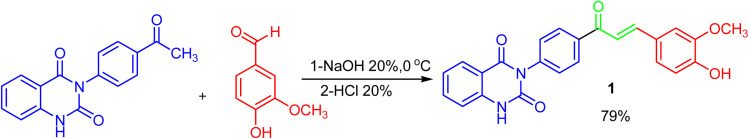
Claisen–Schmidt condensation for synthesis of chalcone 1.

Given the urgent need for new antibacterial inhibitors, the hybrid compounds incorporating known bioactive moieties have gained increasing interest. The general procedure for the synthesis of key precursor chalcone [(*E*)– 3-{4-[3-(4-hydroxy-3-methoxy-phenyl)-acryloyl]-phenyl}-1*H*-quinazolin-2,4-dione] 1 is performed by treatment of 3-(4-acetyl-phenyl)-1*H*-quinazolin-2,4-dione with vanillin *via* Claisen–Schmidt condensation reaction, as depicted in Scheme 1. This reaction, catalyzed by amount of base NaOH, achieved yield of up to 79%. The spectral data and elemental analysis were utilized for identification of chemical structure of α–β-unsaturated carbonyl compound 1. The FT-IR spectrum showed the characteristic absorption bands at 1727 cm^−1^ (C=O carbonyl stretching) due to the conjugation of π-electrons of benzene ring and olefinic linkage. ^1^H-NMR spectrum showed the characteristic signals as two distinct doublets at *δ* 8.30 and 8.33 ppm attributed to olefinic protons adjacent to carbonyl group. The coupling constant (*J* value) of the doublets is 13.00 Hz characterized of an *E*-configuration (Trans). The other peaks appeared at *δ* 11.57 ppm for –NH-quinazoline, in addition to eleven phenyl protons as multiplet at *δ* 7.01–8.04 ppm, and a singlet at *δ* 3.89 ppm for methoxy group –OCH_3_. Also, ^13^C-NMR spectrum showed characteristic signals for –(C=O) at *δ* 191 ppm, *δ* 122 and 145 ppm for olefinic carbons, and *δ* 55 ppm for –OCH_3_. Finally, the structure of 1 was confirmed by its mass spectrum (*m/z* = 414) which agreed with the molecular formula (see Experimental part).

**Scheme 2:**
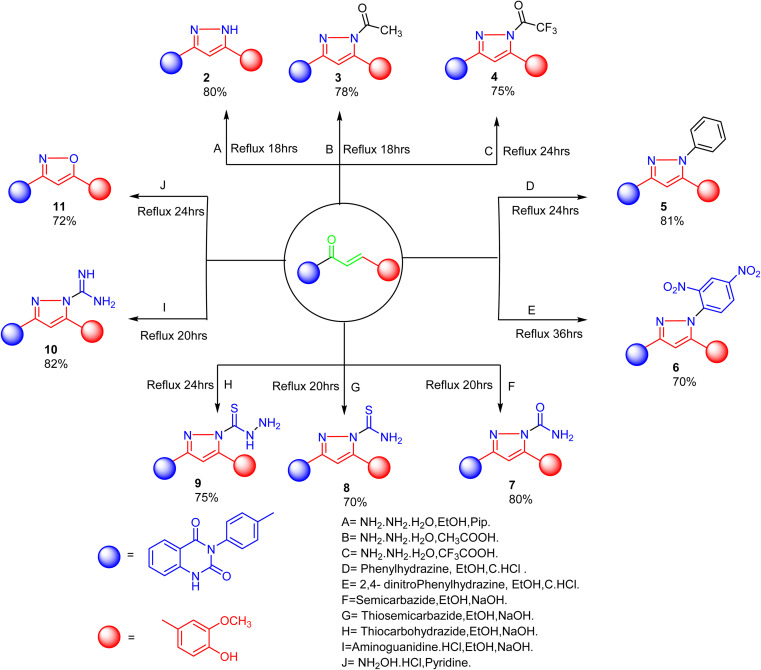
Synthesis of a series of vanillin linked to quinazolin-2,4-dione skeleton through five-membered nitrogen-containing heterocycles 2–11.

**Scheme 3:**
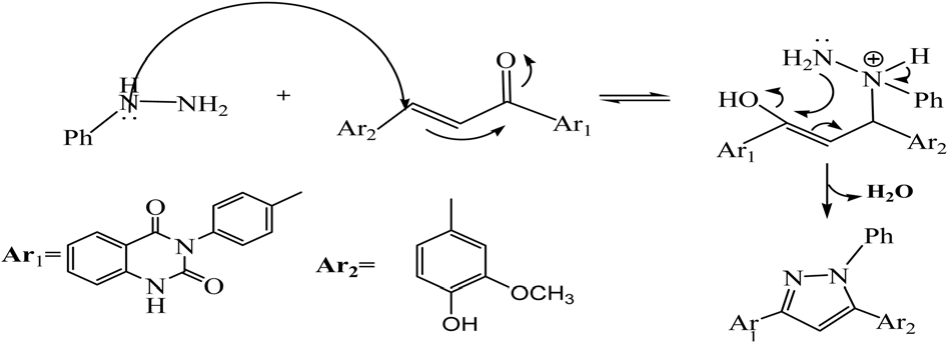
The suggested mechanism for the synthesis of compound 5.

The product 1 is utilized as a good intermediate for a facile synthesis of quinazolin-2,4-dione skeleton through five- and/or six-membered nitrogen-containing heterocycles such as pyrazole, isoxazole and/or pyrimidine 2–15, as declared in Schemes 2 and 3.

As depicted in Scheme 2, treatment of chalcone 1 with an equimolar amount of different nitrogen nucleophiles namely, hydrazine, phenyl hydrazine, 2,4-dinitro phenylhydrazine, semicarbazide, thiosemicarbazide, thiocarbohydrazide, aminoguanidine and/or hydroxylamine under thermal conditions afforded a series of vanillin linked to quinazolin-2,4-dione skeleton through five-membered nitrogen-containing heterocycles 2–11, respectively in good yields. The hydrazinolysis of chalcone 1 in ethanol under reflux afforded the compound 2. On the other hand, the addition of acetic acid and/or trifluoroacetic acid (TFA) provided the expected pyrazoles 3 and 4, respectively. The reaction of hydrazine (binucleophile) with chalcone 1 was carried out utilizing aza-Michael addition, leading to the reaction, then 1,5-*exo*-trig cyclization, which is followed by dehydration. For derivatives 2–4, The FT-IR spectral data exhibited absorption bands at 3210–3301 and 1597–1604 cm^−1^ confirming the presence of NH and C=N groups of pyrazole moieties, correspondingly. In addition, ^13^C-NMR of compound 3 exhibited a characteristic signals at *δ* 26.91 ppm for –COCH_3_, 149.95 ppm for –C=N and 169.45 ppm for –COCH_3_.

**Fig. 3:**
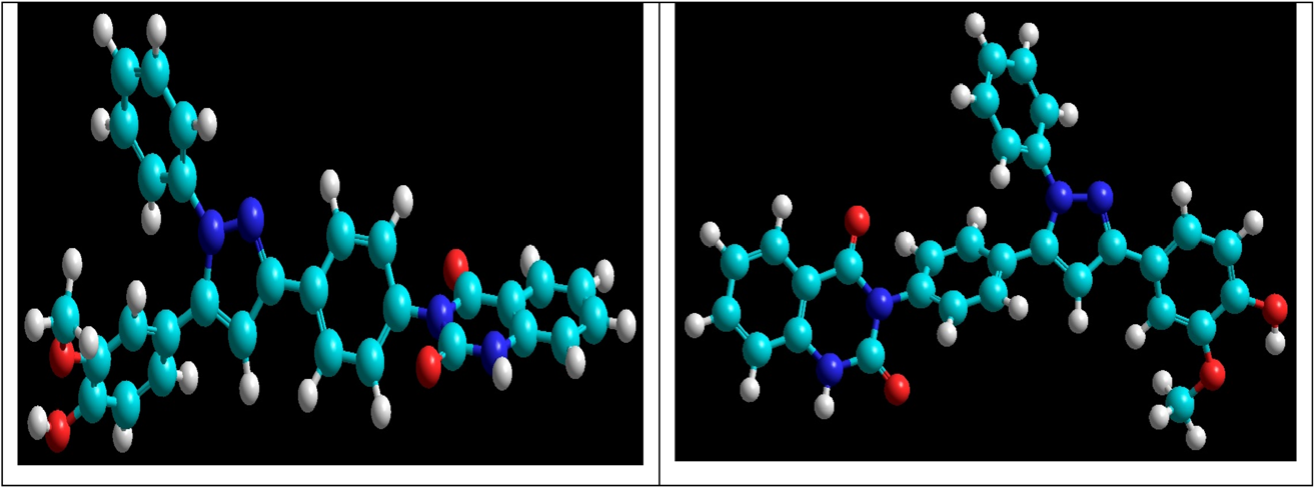
Geometrical optimized structures of compound 5. Left side is more energetically favorable conformer than right side one.

The reaction of chalcone 1 with phenyl hydrazine and/or 2,4-dinitro phenylhydrazine under reflux yielded the pyrazoles 5–6, respectively. The suggested mechanism for the synthesis of compound 5, is represented in Scheme 3. Moreover, PM6 geometrical optimization and heat of formation of two expected conformers of product 5 confirmed that the left side conformer (57.34347 kcal mol^−1^) is more energetically stable than the right side one (57.31583 kcal mol^−1^) by 27.64 cal mol^−1^, as declared in Fig. 3.

Additionally, treatment of compound 1 with semicarbazide, thiosemicarbazide and thiocarbohydrazide afforded the compounds 7–9, respectively. Then, the treatment of the chalcone 1 with aminoguanidine in ethanol with catalytic amount of NaOH under reflux gave compound 10. Finally, addition of hydroxylamine to compound 1 in refluxed pyridine afforded isoxazole 11. The newly prepared compounds were structurally confirmed by their spectral analyses. The FT-IR spectral data showed the disappearance of the characteristic peak for conjugated carbonyl group (–C=C–CO–) at 1727 cm^−1^, along with C=C double bond stretching band at 1577 cm^−1^ and the appearance of NH stretching band at 3288 cm^−1^. The ^1^H-NMR spectra declared the absence of signals related to the CH=CH group of the compound 1, with the appearance of new signal related to olefinic CH group (pyrazole rings) in interference with aromatic proton at *δ* 6.52–8.26 ppm (see Experimental part).

**Scheme 4:**
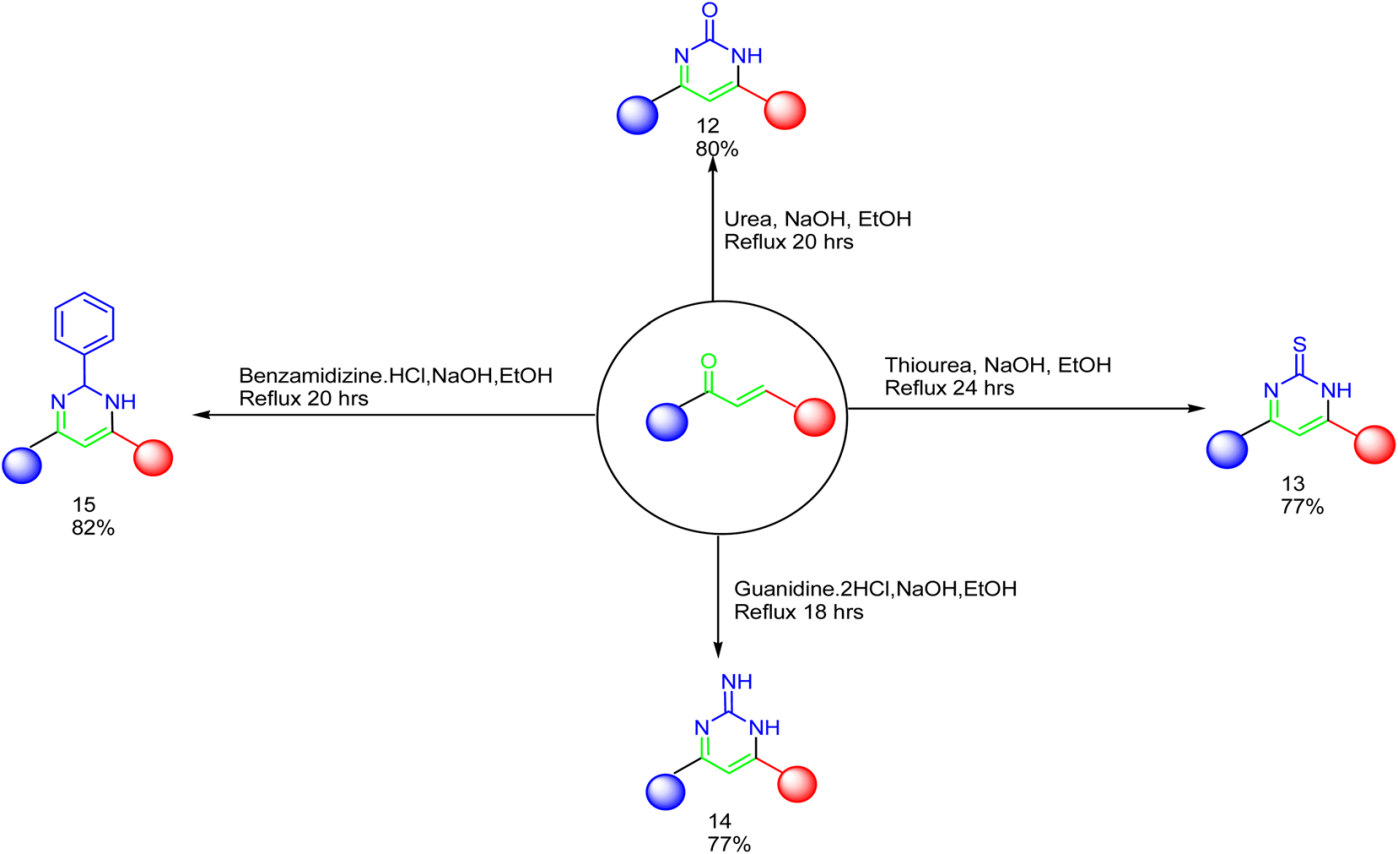
Synthesis of a series of vanillin-attached quinazolin-2,4-dione skeleton through pyrimidine ring 13–15.

As declared in Scheme 4, cyclization of chalcone 1 with an equimolar amount of various nitrogen nucleophiles namely, urea, thiourea, guanidine and/or benzamidinium chloride yielded a series of vanillin-attached quinazolin-2,4-dione skeleton through six-membered nitrogen-containing heterocycles (pyrimidine) 12–15, respectively, in good yields. The spectral data and elemental analysis were utilized for identification of chemical structures of compounds. FT-IR spectra of these compounds exhibited characteristic absorption bands at 3315–3192 for NH, and 1720–1669 cm^−1^ for C=O groups. Considering the ^1^H-NMR spectra of new pyrimidine derivatives 12–15, NH protons of quinazolin-2,4-dione skeletons were observed as singlet signals at the range *δ* 11.50–11.63 ppm. In addition, the aromatic protons were observed at *δ* 6.58–8.39 ppm. A table of comparison with the previously published reports^36,37^ is included in the SI File, as Table S1. The spectral data of all prepared compounds are included in the SI File, as Fig. S1–S57.

### 2.2 Antibacterial activity

Microbial chemotherapeutic resistance considered as a serious threat to global public health, reducing the effectiveness of antimicrobial drugs and causing infections to become difficult or impossible to treat. A critical strategy for combating this challenge is the development of new antimicrobials, including novel heterocyclic compounds. In our study, the antibacterial assay was performed for fifteen quinazolin-2,4-diones incorporating vanillin connecting by biologically active five- and/or six-membered nitrogen rings. These hybrid compounds were tested for their antibacterial activity against two Gram +ve strains, *i.e.* (*Bacillus subtilis* ATCC 6633 and *Staphylococcus aureus* NRRL B-767) and two Gram −ve strains, *i.e.* (*Escherichia coli* ATCC 25955, *Pseudomonas aeruginosa* ATCC 10145). The MIC of the tested novel compounds was also mentioned, as shown in Table 1.

**Table 1:** Antimicrobial efficacy and minimum inhibitory concentration (MIC) of the compounds and standard drug^a^

Sample no.	Minimum inhibitory concentration (MIC, µg mL^−1^)			
	*Escherichia coli*	*Pseudomonas aeruginosa*	*Bacillus subtilis*	*Staphylococcus aureus*
1	160 ± 0.28	80 ± 0.14	ND	40
2	2.5 ± 0.18	5 ± 0.25	10 ± 0.22	2.5 ± 0.51
3	80 ± 0.03	40 ± 0.21	ND	16 ± 0.22
4	40 ± 0.15	20 ± 0.15	ND	ND
5	2.5 ± 0.08	5 ± 0.18	10 ± 0.02	5 ± 0.07
6	10 ± 0.09	15 ± 0.17	40 ± 0.18	160 ± 0.80
7	20 ± 0.11	5 ± 0.20	10 ± 0.25	20 ± 0.14
8	20 ± 0.12	40 ± 0.15	80 ± 0.45	ND
9	ND	160 ± 0.17	40 ± 0.21	ND
10	120 ± 0.05	40 ± 0.31	20 ± 0.05	15 ± 0.23
11	10 ± 0.04	5 ± 0.01	2.5 ± 0.08	5 ± 0.05
12	ND	16 ± 0.07	7 ± 0.14	ND
13	10 ± 0.11	40 ± 0.18	80 ± 0.16	120 ± 0.16
14	40 ± 0.14	ND	80 ± 0.11	ND
15	20 ± 0.17	15 ± 0.10	10 ± 0.24	10 ± 0.03
Ciprofloxacin	5 ± 0.11	7 ± 0.33	2.5 ± 0.17	1.25 ± 0.05

a Ciprofloxacin was used as standard drugs as control,^38,39^ ND: not determined.

Minimum inhibition concentration (MIC) was defined as the average of the lowest concentrations with no observation of microbial growth. The results obtained revealed that compounds 2, 5 and 11 exhibited significant antimicrobial potency against all the tested microbes at low concentration ranging from 2.5 to 10 µg mL^−1^. The results showed also, that compounds 7 and 15 exhibited a considerable wide broad spectrum of antibacterial potency against all the strains of the tested pathogenic bacteria with low concentrations ranged from 5 to 20 µg mL^−1^. Otherwise, compound 6 revealed significant antimicrobial effect at low concentrations against the two tested Gram-negative bacteria (*Escherichia coli* ATCC 25955 and *Pseudomonas aeruginosa* ATCC 10145). Meanwhile, compound 10 showed strong antibacterial effect at relatively low concentrations against the two tested Gram-positive bacteria (*Bacillus subtilis* ATCC 6633 and *Staphylococcus aureus* NRRL B-767).

### 2.3. Structure–activity relationship

In this part, we summarize the effect of substituents on antibacterial activity of the newly synthesized compounds. The enhanced antibacterial activity of the molecules 2, 5 and 11 was ascribed to the presence of electron donating-groups –OH, and –OCH_3_ attached to pyrazole and/or isoxazole moieties, respectively. In addition, aromatic and heteroaromatic rings are favored as hydrophobic heads over aliphatic and alicyclic moieties.

### 2.4. Computational studies

#### 2.4.1. *In silico* docking studies

**Fig. 4:**
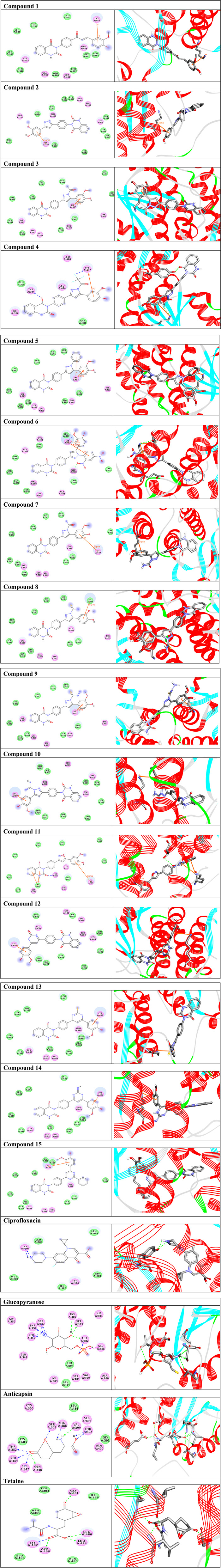
2D (Left side) and 3D interactions (right side) of the compounds, ciprofloxacin, co-crystalized ligand and two references with glucosamine-6-phosphate synthase. Left side; compounds are represented in line models, and amino acid residues are shown in balls. Hydrogen bonds are shown in blue and green dotted lines, and Pi-stacked are represented in orange lines. Right side; compounds are represented in stick models, and residues are declared in gray stick color. Hydrogen bonds are shown in green dotted lines, and Pi-stacked are represented in orange lines.

Glucosamine-6-phosphate (GlcN-6-P) synthase is an important enzyme in the synthesis UDP-*N*-acetyl glucosamine.^40,41^ Herein, GlcN-6-P has gained a huge attention as promising target for identification of new drug candidates for bacterial infections. The docking approaches^42,43^ were performed for all synthesized molecules and standard drug with the active site residues^44,45^ of the target, utilizing PyRx-virtual screening software, to elucidate the interaction strength of various moieties with the target enzyme. The binding scores correlated with the IC_50_ values of the compounds, with energy values ranging from −9.9 to −7.4 kcal mol^−1^. The 2D and 3D structures of the docked complexes were analyzed utilizing Discovery Studio 3.5 Visualizer as shown in Fig. 4. In Table 2, the chemical structures/IUPAC names of the compounds, binding energy (kcal mol^−1^) along with interactions and distances (Å) are depicted. The standard ciprofloxacin exhibited the binding energy −5.4 kcal mol^−1^ and showed two hydrogen bond interactions with the residue TYR476 at 2.84 and 2.46 Å, respectively. The Chalcone 1 docked to the target through one π-cation interaction with the residue LYS487 at 4.15 Å. Compound 2 with binding energy −9.7 kcal mol^−1^ showed one π–sigma interaction with the target at 3.76 Å. Compound 5 (−9.9 kcal mol^−1^) exhibited one π-cation and one π-sigma interactions with the residue LYS487 at 4.36 and 3.91 Å, respectively. Compound 12 declared π-cation interaction with the residue LYS487 at 4.28 Å. The enhanced antibacterial activity of the best docked compounds 2, 5 and 12 was ascribed to the presence of electron donating-groups –OH, and –OCH_3_ attached to aromatic and heteroaromatic rings such as pyrazole and/or isoxazole.

**Table 2:** The binding scores (kcal mol^−1^) and interactions between the compounds, ciprofloxacin, co-crystalized ligand and two references with the target enzyme (GlcN-6-P) synthase

	2D Structure/name	Binding energy kcal mol^−1^	Docked complex (amino acid–ligand) interactions	Distance (Å)
1	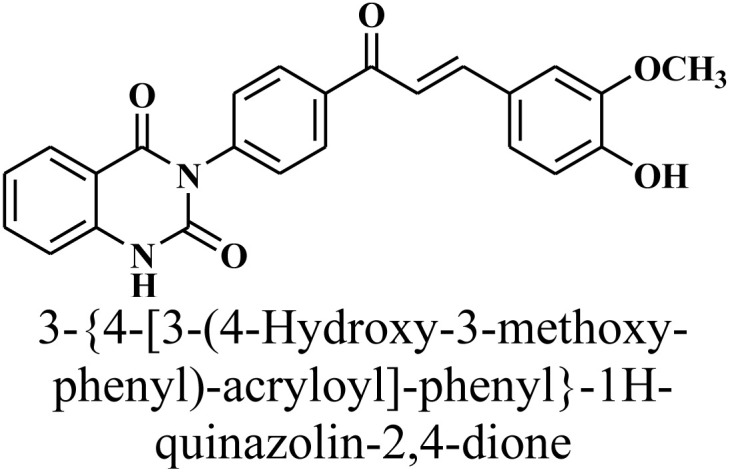	−7.6	π-Cation interaction	4.15
			LYS487:NZ⋯compound 1	
2	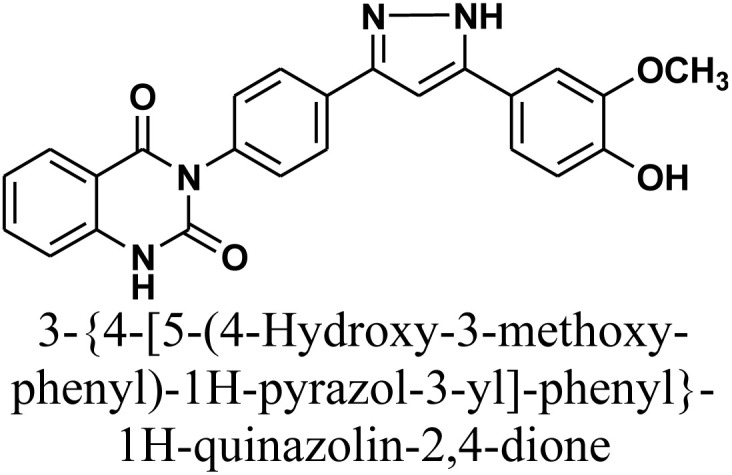	−9.7	π-Sigma interaction	3.76
			LYS487:NZ⋯compound 2	
3	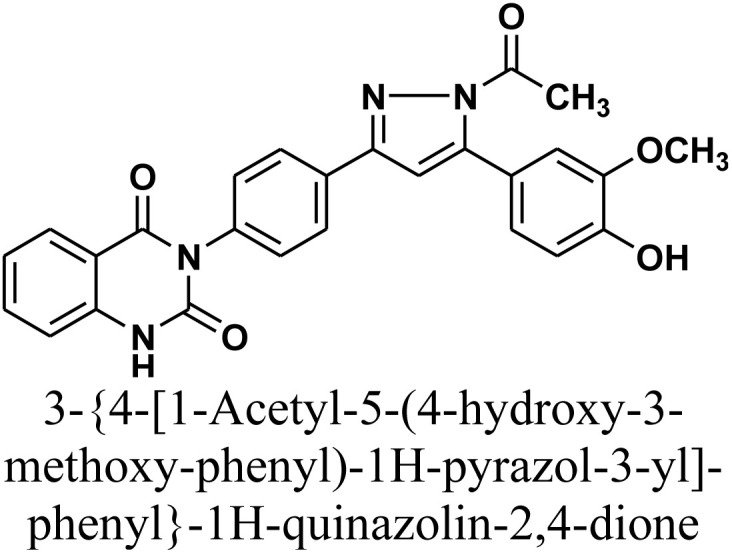	−9.5	π-Sigma interaction	3.90
			LYS487:NZ⋯compound 3	
4	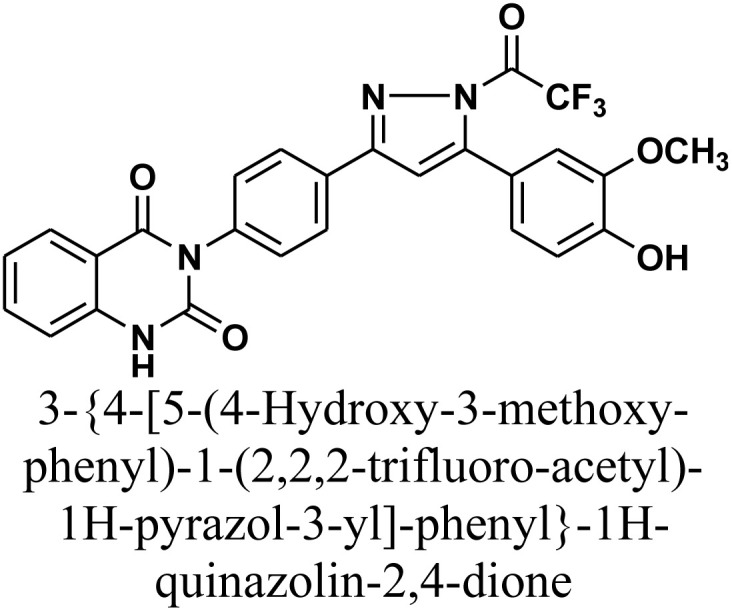	−8.7	H-Bonds	2.70
			TYR304:OH⋯compound 4	
			LYS487:NZ⋯compound 4	2.96
			π-Cation interaction	5.39
			LYS487:NZ⋯compound 4	
5	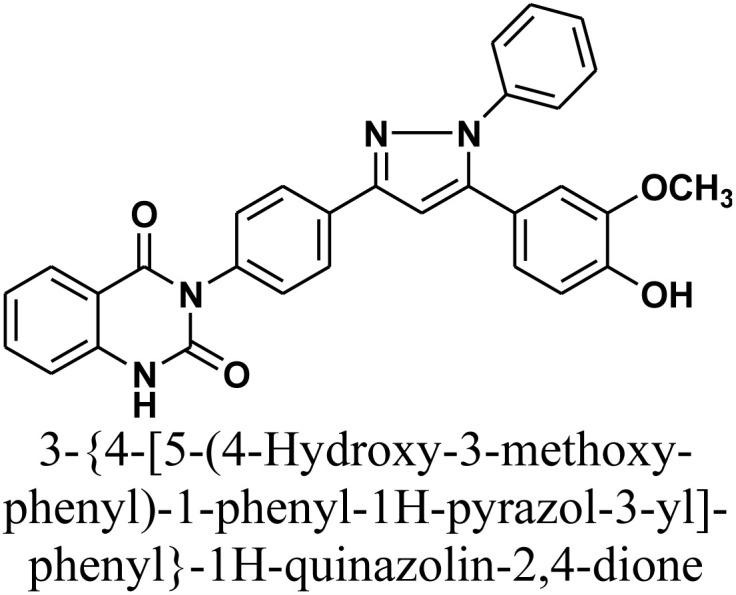	−9.9	π-Cation interaction	4.36
			LYS487:NZ⋯compound 5	
			π-Sigma interaction	3.91
			LYS487:CD⋯compound 5	
6	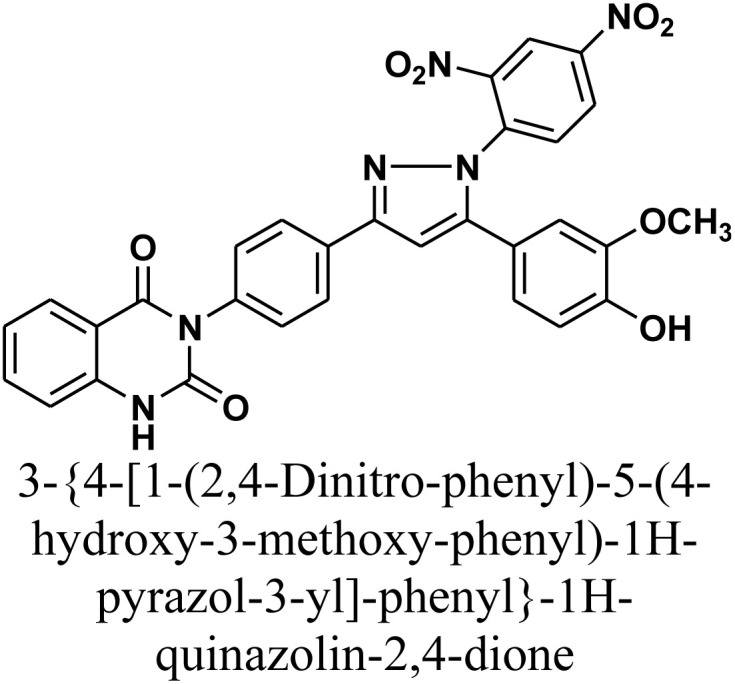	−9.3	H-Bonds	2.31
			LYS487:NZ⋯compound 6	
			GLU488:N⋯compound 6	3.00
			π-Cation interaction	4.17
			LYS487:NZ⋯compound 6	
			LYS487:NZ⋯compound 6	6.00
			π-Sigma interaction	3.90
			LYS487:CD⋯compound 6	
7	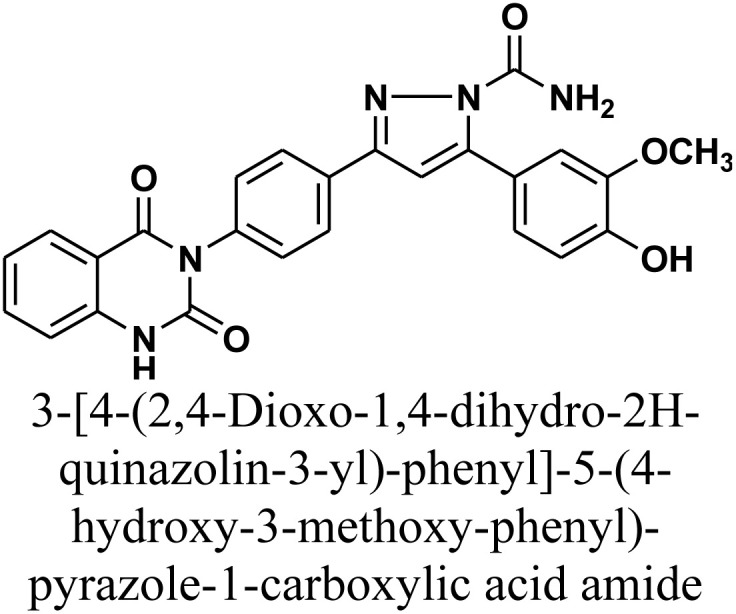	−9.4	π-Sigma interaction	3.86
			LYS487:CD⋯compound 7	
8	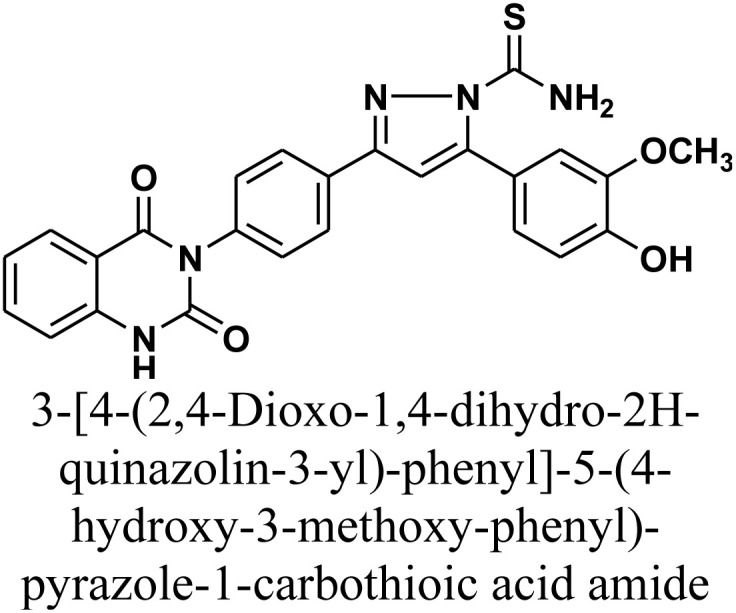	−9.2	π-Sigma interaction	3.79
			LYS487:CB⋯compound 8	
9	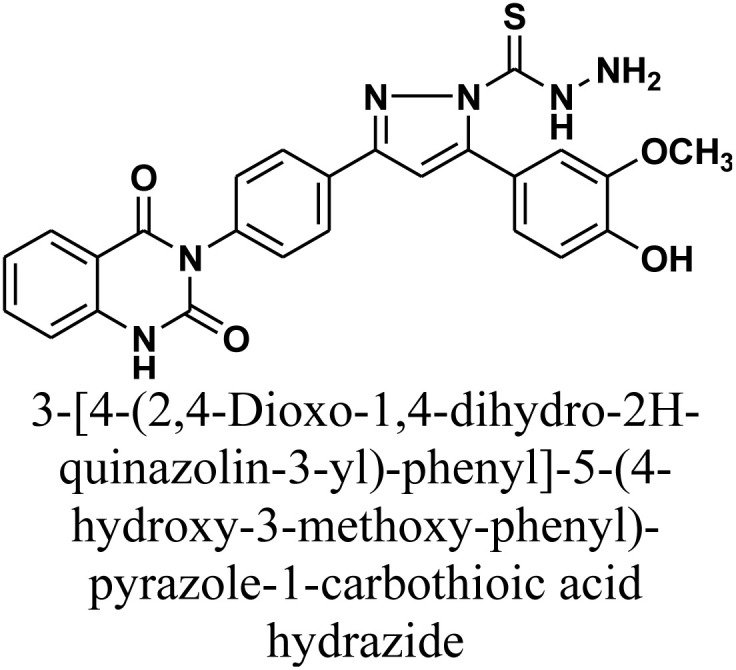	−8.9	π-Sigma interaction	3.88
			LYS487:CB⋯compound 9	
10	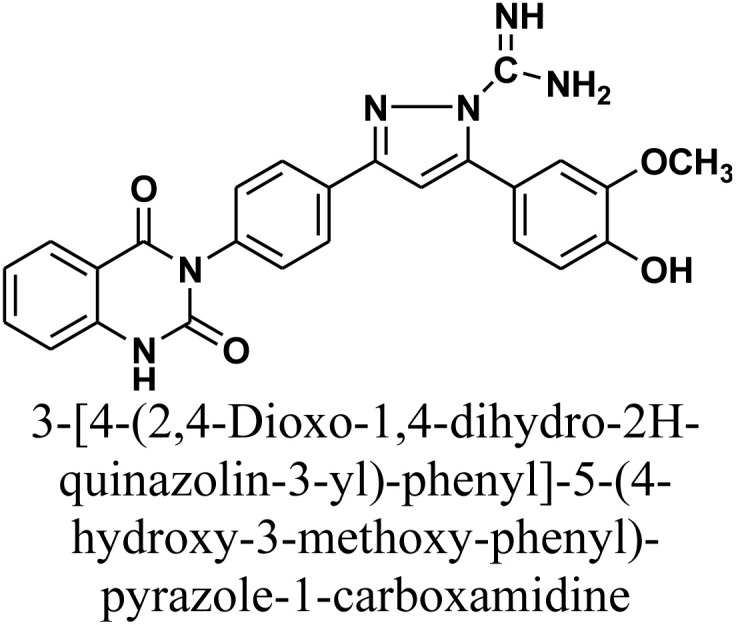	−8.8	π-Cation interaction	4.33
			LYS487:NZ⋯compound 10	
11	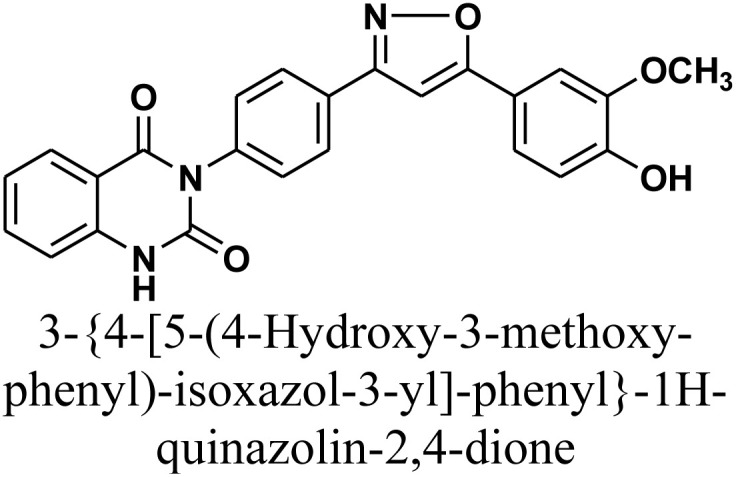	−9.7	H-Bonds	2.99
			THR302:OG1⋯compound 11	
			π-Cation interaction	6.00
			LYS603:NZ⋯compound 11	
			LYS603:NZ⋯compound 11	5.86
			π-Sigma interaction	3.90
			LYS487:CB⋯compound 11	
12	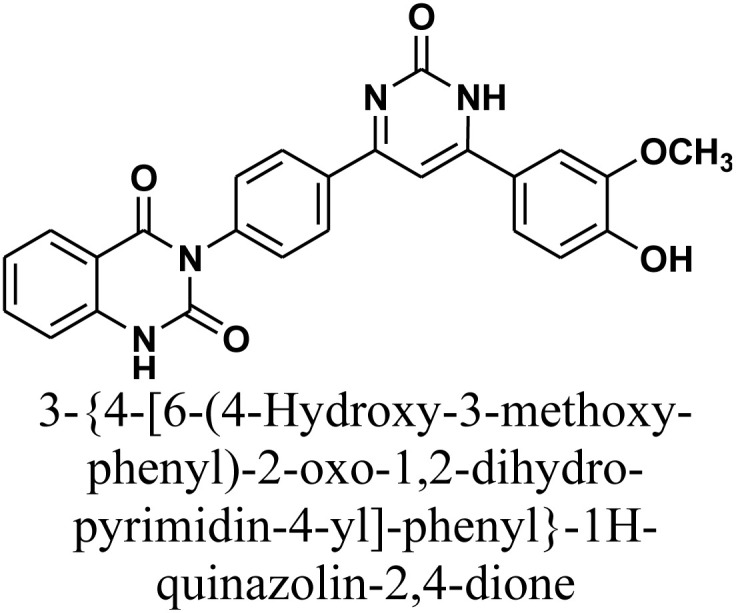	−9.6	π-Cation interaction	4.29
			LYS487:NZ⋯compound 12	
13	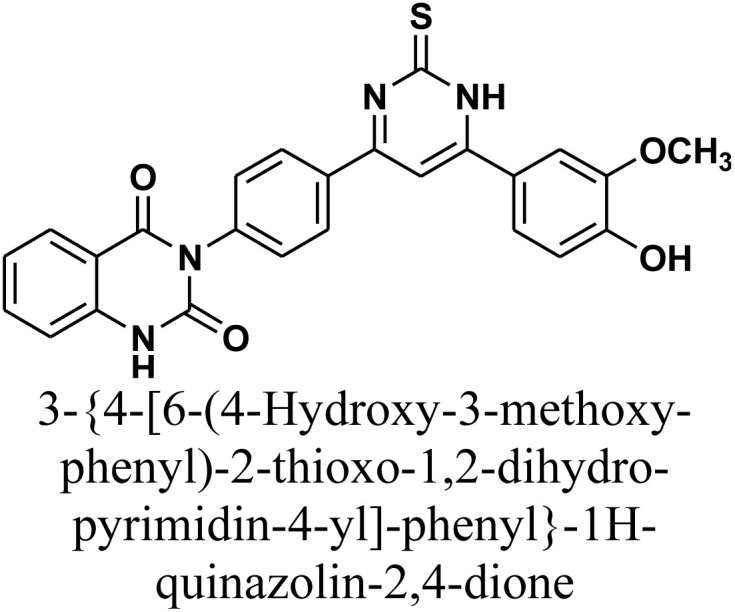	−7.4	π-Cation interaction	4.24
			LYS487:NZ⋯compound 13	
14	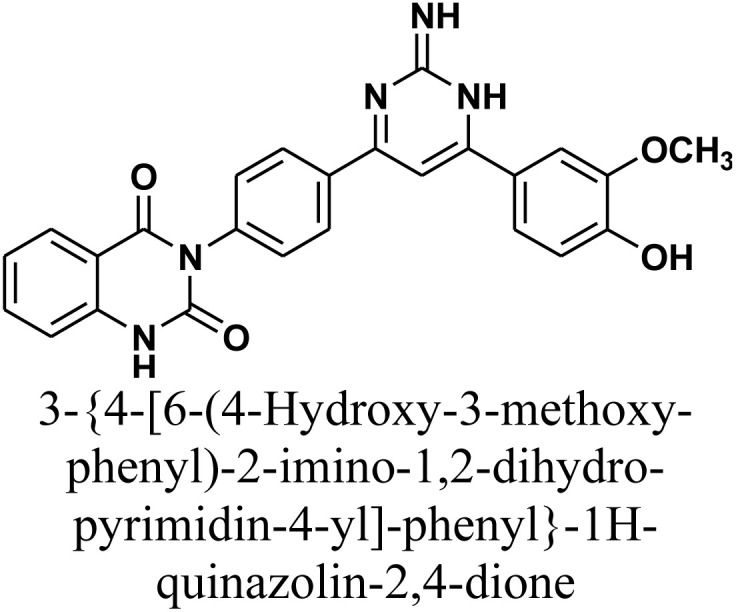	−8.4	π-Cation interaction	4.27
			LYS487:NZ⋯compound 14	
15	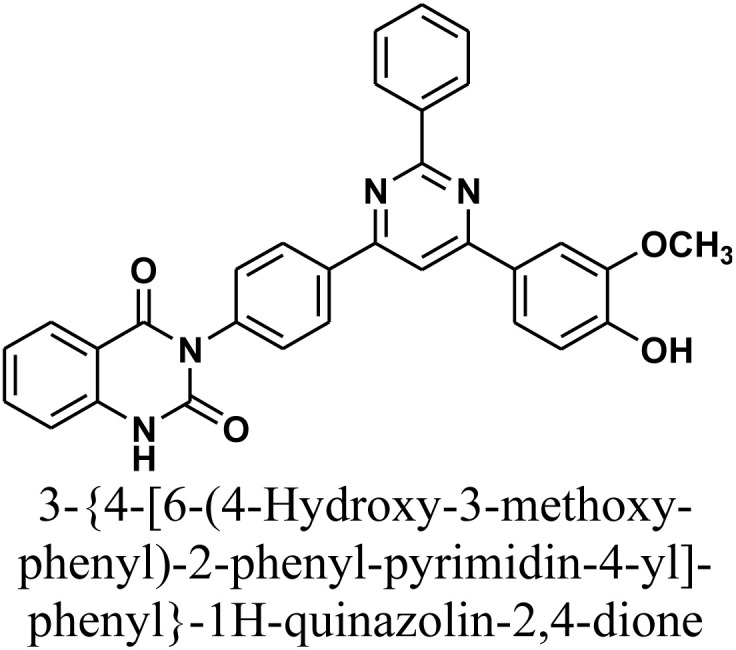	−9.3	π-Cation interaction	4.11
			LYS487:NZ⋯compound 15	
Cipro-floxacin	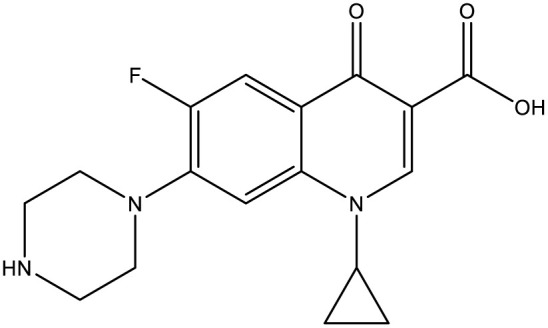	−10.6	H-Bonds	2.84
			TYR476:OH⋯ciprofloxacin	
			TYR476:OH⋯ciprofloxacin	2.46
Gluco-pyranose	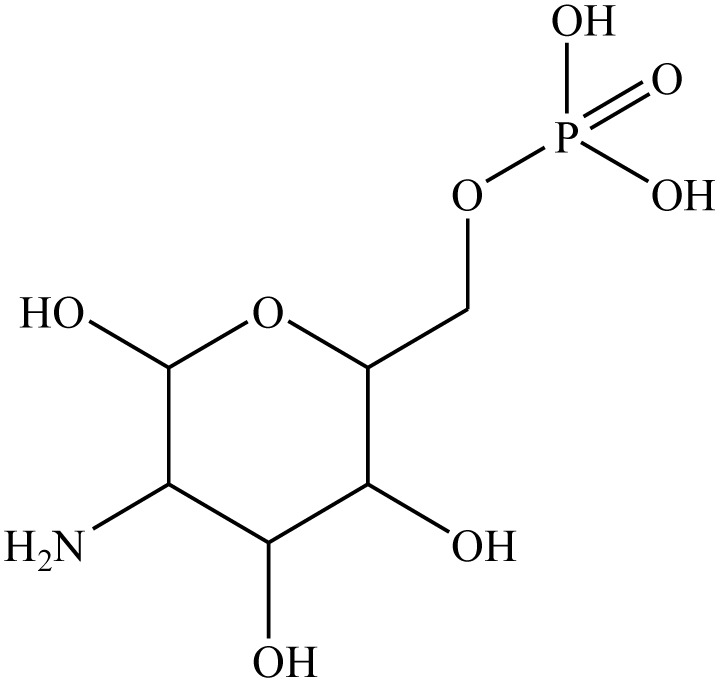	−9.9	H-Bonds	2.95
			THR302:N⋯glucopyranose	
			THR302:OG1⋯glucopyranose	2.91
			SER303:N⋯glucopyranose	3.00
Anti-capsin	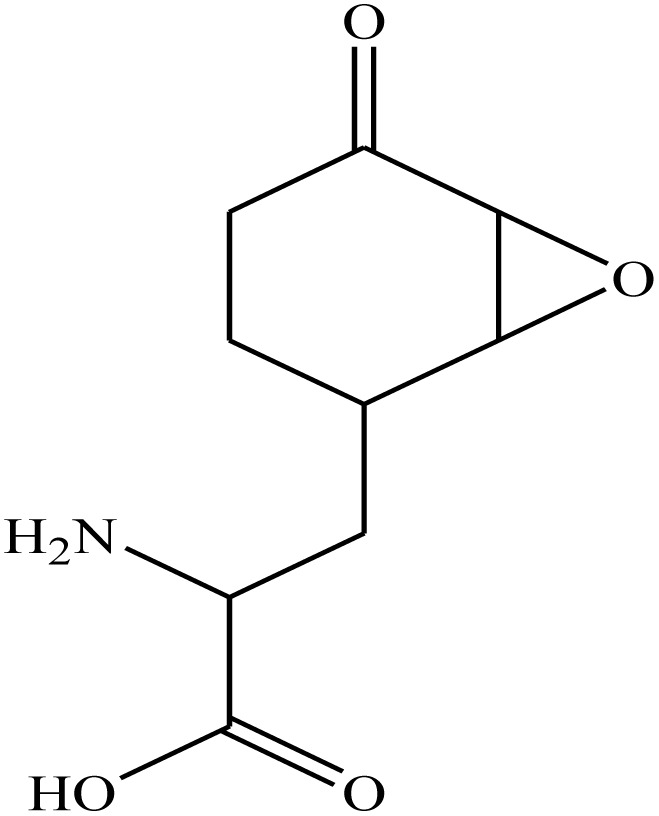	−7.0	H-Bonds	3.00
			THR302:N⋯anticapsin	
			THR302:OG1⋯anticapsin	3.00
			SER303:OG⋯anticapsin	2.74
			SER347:OG⋯anticapsin	2.87
			GLN348:N⋯anticapsin	2.99
			SER349:N⋯anticapsin	3.00
			SER349:OG⋯anticapsin	2.88
			THR352:OG1⋯anticapsin	3.00
			GLU488:OE2⋯anticapsin	2.28
			VAL399:O⋯anticapsin	2.01
			THR302:OG1⋯anticapsin	2.08
Teta-ine	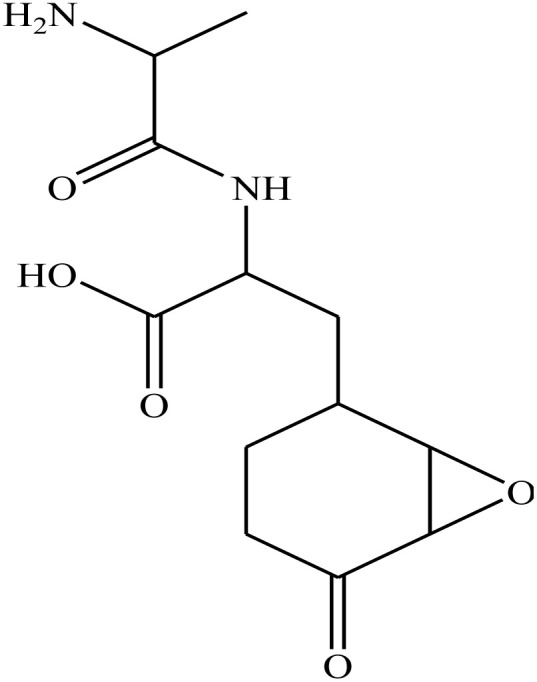	−5.4	H-Bonds	2.65
			GLY301:N⋯tetaine	
			LEU484:N⋯tetaine	2.99
			LYS487:NZ⋯tetaine	3.00
			ALA496:N⋯tetaine	2.85
			LEU480:O⋯tetaine	2.17

Additionally, the redocking process of 2-amino-2-deoxy-6-*O*-phosphono-alpha-d-glucopyranose (a co-crystallized ligand) with the active site of GlcN-6-P enzyme was performed (RMSD ˂ 2 Å) and the results exhibited similar fitness to the docked molecules. 2-Amino-2-deoxy-6-*O*-phosphono-alpha-d-glucopyranose showed nine H-bond interactions with CYS300, THR302, SER303, SER347, SER349, THR352 at 2.91, 3.00, 2.81, 2.80, 2.93, 2.84, 2.14, 2.15 and 2.01 Å, respectively (Table 2).

Finally, anticapsin (a reference drug 1) showed eleven HB interactions with THR302, SER303, SER347, GLN348, SER349, THR352, GLU488, and VAL399 at 3.00, 2.74, 2.87, 2.99, 2.88, 2.28, 2.01and 2.08 Å, respectively. Whereas, tetaine (a reference drug 2) exhibited five HB interactions with the active site residues of GlcN-6-P enzyme^44^ (Table 2).

Moreover, ADMET properties of the compounds were simulated to determine their potential as drug candidates against bacterial diseases, as declared in Table 3. The hydrogen bond donors (HBD) and hydrogen bond acceptors (HBA) of the tested compounds are in acceptable range (≤10) and (≤5), respectively; indicating high solubility of these compounds. In addition, they have good absorption properties (%HIA). Their lipophilicities (measured by MLOGP) are in an optimal value. Further, they exhibited high gastrointestinal GI absorption. None of them exhibited blood–brain barrier (BBB) permeability; indicating no side effect on central nervous system CNS. When taken as a whole, the pharmacokinetics properties of the compounds agreed with Lipinski's rule of five; confirming that could serve as antibacterial drug candidates.

**Table 3:** ADMET profile and drug-likeness properties of the docked molecules, ciprofloxacin, co-crystalized ligand and two references^a^

	Molecular weight (g mol^−1^)	BBB permeant	%Human intestinal absorption (HIA+)	Log *p*	TPSA A^2^	HBA	HBD	N rotatable	N violations	Bioavailability	GI absorption	AMES toxicity	Carcinogenicity
1	414.41	No	98.21	2.84	101.39	5	2	5	0	0.55	High	Nontoxic	Noncarcinogenic
2	426.42	No	98.98	2.83	113.00	5	3	4	0	0.55	High	Nontoxic	Noncarcinogenic
3	468.46	No	99.62	3.21	119.21	6	2	5	0	0.55	High	Nontoxic	Noncarcinogenic
4	522.43	No	100.00	3.51	119.21	9	2	6	0	0.55	Low	Nontoxic	Noncarcinogenic
5	502.52	No	99.63	4.10	102.14	5	2	5	0	0.55	High	Nontoxic	Noncarcinogenic
6	592.52	No	98.87	2.53	193.78	9	2	7	0	0.55	Low	Nontoxic	Noncarcinogenic
7	469.45	No	99.67	2.91	145.23	6	3	5	0	0.55	Low	Nontoxic	Noncarcinogenic
8	485.51	No	98.94	2.87	160.25	5	3	5	0	0.55	Low	Nontoxic	Noncarcinogenic
9	500.53	No	96.65	2.91	172.28	6	4	6	0	0.55	Low	Nontoxic	Noncarcinogenic
10	468.46	No	99.09	2.91	152.01	6	4	5	0	0.55	Low	Nontoxic	Noncarcinogenic
11	427.41	No	99.43	2.83	110.35	6	2	4	0	0.55	High	Nontoxic	Noncarcinogenic
12	454.43	No	96.32	2.57	130.07	6	3	4	0	0.55	High	Nontoxic	Noncarcinogenic
13	470.50	No	78.46	2.53	145.09	5	3	4	0	0.55	Low	Nontoxic	Noncarcinogenic
14	453.45	No	92.55	2.57	136.85	6	4	6	0	0.55	Low	Nontoxic	Noncarcinogenic
15	514.53	No	98.54	3.85	110.10	6	2	5	0	0.55	High	Nontoxic	Noncarcinogenic
Ciprofloxacin	331.34	No	97.95	1.28	74.57	5	2	3	0	0.55	High	Nontoxic	Noncarcinogenic
Glucopyranose	259.15	No	86.28	−3.64	172.51	9	6	3	1	0.11	Low	Nontoxic	Noncarcinogenic
Anticapsin	199.20	No	88.76	3.00	92.92	5	2	3	0	0.55	High	Nontoxic	Noncarcinogenic
Tetaine	270.28	No	86.23	−1.21	122.02	6	3	6	0	0.55	High	Nontoxic	Noncarcinogenic

a Log *p*, logarithm of partition coefficient between *n*-octanol and water; *n* rotatable, number of rotatable bonds; TPSA, topological polar surface area; HBA, number of hydrogen bond acceptors; HBD, number of hydrogen bond donors. The acceptable ranges are as follows; Mol wt: (130–500); %Human oral absorption: >80% high, <25% low; donor HB: (0.0–6.0); accept HB: (2.0–20.0).

**Fig. 5:**
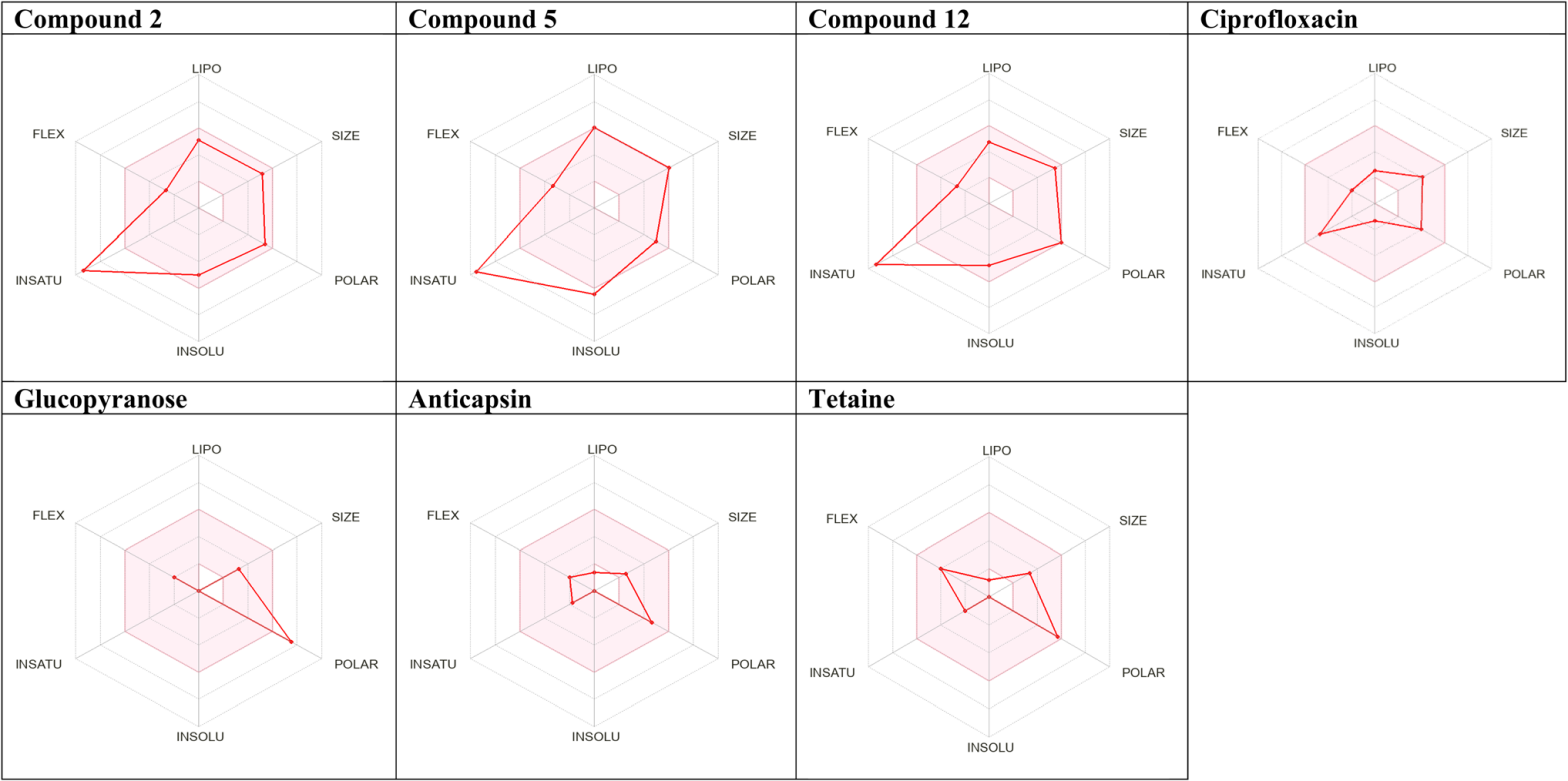
Radar of the best docked compounds, ciprofloxacin, co-crystalized ligand and two references.

In addition, Fig. 5 shows the physicochemical characters such as solubility, polarity, lipophilicity, saturation, flexibility, and size of compounds 2, 5 and 12 “bioavailability radar”. The inner pink indicates the optimal values for acceptable bioavailability. The molecular weights of compounds and afatinib are in acceptable range (˂500 g mol^−1^). The compounds and afatinib have rotatable bonds within the allowed range (<10 bond) that enhance their flexibility. To conclude, compounds 2, 5 and 12 are predicted to have acceptable bioavailability.

#### 2.4.2. Semiempirical study

**Fig. 6:**
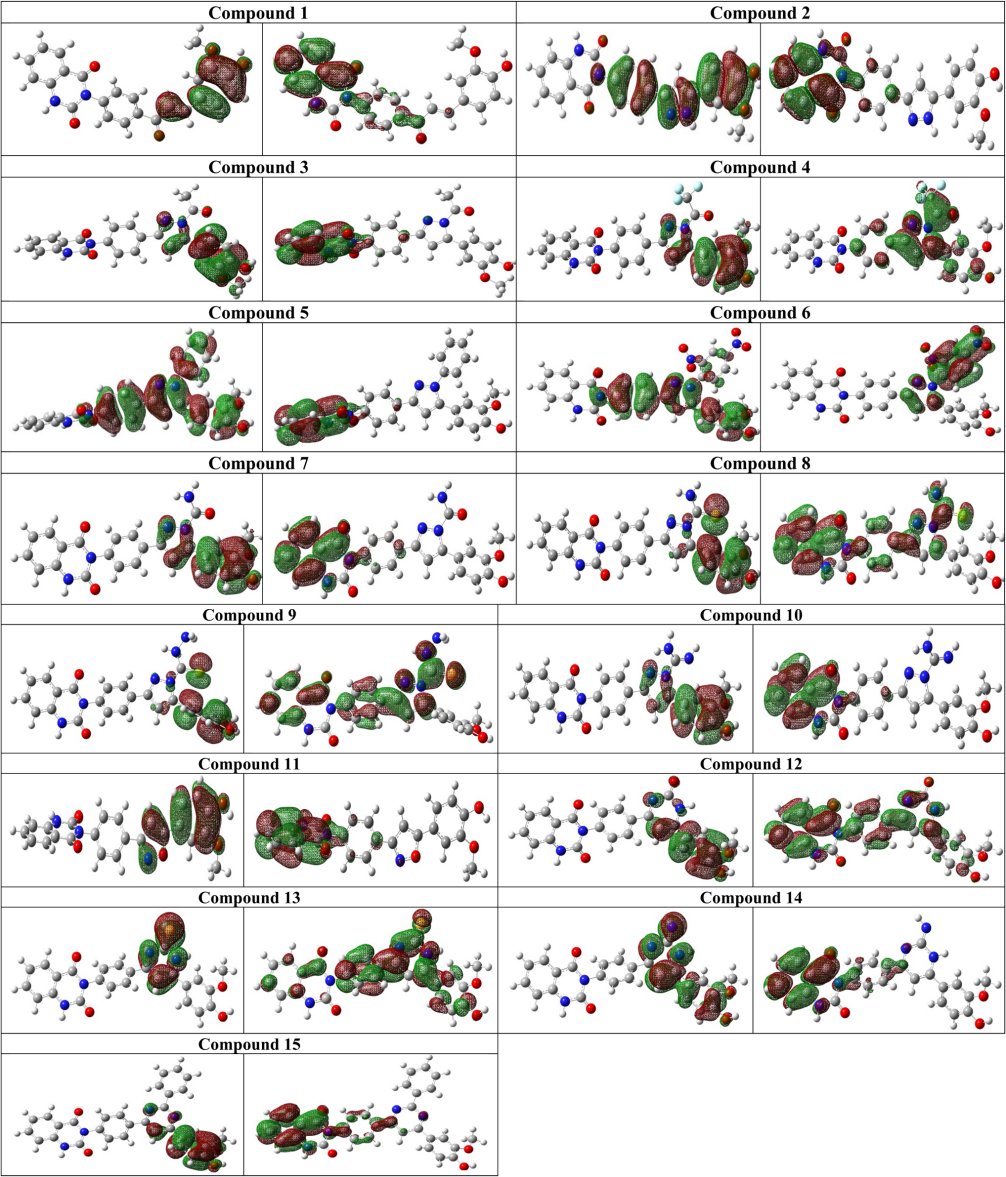
Molecular orbitals of all docked compounds. HOMO (left side) and LUMO (right side).

A PM6 semiempirical calculation exhibited HOMO (highest occupied molecular orbital) and LUMO (lowest unoccupied molecular orbital) of all compounds 1–15 (Fig. 6). The π donor delocalized electron density is condensed on substituted pyrazole rings of the compound 2–10 (as shown in HOMO orbitals). Meanwhile, the π acceptor electron cloud is localized on quinazolin-2,4-dione skeleton as shown in LUMO orbitals for compounds 2, 3, 5, 7, 8, 9 and 10. The existence of electron-withdrawing groups such as (–COCF_3_ and dinitro at 2-, and 4-position on phenyl ring) in compounds 4 and 6, respectively, shifted the electron cloud deficiency from the quinazolin-2,4-dione skeleton to the opposite sides. Thus donor –π- acceptor system (D–π–A) of pyrazole/isoxazole/pyrimidine of compound 2–15 played pivotal role in the Charge Transfer Interaction (CTI) with the active site of the target enzyme. However, compounds 4 and 6 show acceptor delocalized electron clouds on (–COCF_3_ and dinitro at 2-, and 4-position on phenyl ring), facilities charge transfer interaction with the active site LYS487 and GLU488, respectively.

## 3. Materials and methods

### 3.1. Chemistry

All chemicals and solvents were obtained from Aldrich Chemical Co. and used without purification. The melting points of the obtained compounds were investigated on capillary tubes by using MEL-TEMP II, and are uncorrected. The completion of the reactions was monitored by thin layer chromatography TLC using benzene/EtOH (3 : 1) as mobile phase, and the spots were visualized by irradiation with UV light (254 nm). FT-IR, ^1^H&^13^C-NMR and MS spectra were recorded on Shimadzu 408, Bruker Vect. 22, JEOL, and Mass 5988 Mass spectrometer, respectively. Elemental analyses were performed at Cairo University, Egypt.

#### 3.1.1. 3-{4-[3-(4-Hydroxy-3-methoxy-phenyl)-acryloyl]-phenyl}-1*H*-quinazolin-2,4-dione 1

In a dried round-bottom flask, a solution of 3-(4-acetyl-phenyl)-1*H*-quinazolin-2,4-dione (2.80 g, 10 mmol) in absolute ethanol (100 mL), vanillin (2.27 g, 15 mmol) was introduced in the presence of a catalytic amount of 50% NaOH (10 mL), and the mixture was stirred for 2 h at (0 to −10 °C) then stirring completed overnight. Following this, it was poured onto ice-diluted HCl, and the resultant product was filtered out and recrystallized from ethanol to obtain compound 1 as yellow powder. Yield: 79% (3.27 gm); M.P. 220–222 °C. FT-IR (KBr, *ν*, cm^−1^) analysis showed peaks at: 3445 (OH), 3250 (NH), 1727 and 1652 (C=O stretches); ^1^H-NMR (400 MHz, DMSO, *δ*, ppm): 3.89 (s, 3H, OCH_3_), 7.01–8.04 (m, 13H, Ar H), 8.30 (d, 1H, CH = CO), 8.33 (d, 1H, CH = CH-Ph), 11.57 (s, 1H, NH); ^13^C-NMR (100 MHz, DMSO, *δ*, ppm): 55, 114, 115, 117, 118, 122, 127, 128, 129, 135, 139, 140, 145, 149, 162, 169, 191; MS: *m/z* = 414 [M^+^]. Elemental analysis calculated for C_24_H_18_N_2_O_5_: C, 69.56%; H, 4.38%; N, 6.76%. Found: C, 69.85%; H, 4.62%; N, 6.45%.

#### 3.1.2. 3-{4-[5-(4-Hydroxy-3-methoxy-phenyl)-1*H*-pyrazol-3-yl]-phenyl}-1*H*-quinazolin-2,4-dione 2

Reaction of chalcone 1 (0.414 g, 1 mmol) and hydrazine hydrate (0.12 mL, 2 mmol) in ethanol (15 mL) in presence of drops of piperidine as a basic catalyst was heated under reflux for 18 h (monitored by TLC). The precipitate that formed after cooling was collected through filtration, dried, and recrystallized using benzene/ethanol to yield product 2 as white crystals. Yield: 80% (0.34 gm); M.P. 275–277 °C. FT-IR (KBr, *ν*, cm^−1^): 3445 (OH), 3210 (NH), 1720 and 1673 (C=O stretches); ^1^H-NMR (400 MHz, DMSO-d6, *δ*, ppm): 3.90 (s, 3H, OCH_3_), 5.50 (s, 1H, NH), 7.25–8.08 (m, 12H, Ar–H + CH), 11.60 (s, 1H, NH); MS: *m/z* = 426 [M^+^]. Elemental analysis calculated for C_24_H_18_N_4_O_4_: C, 67.60%; H, 4.25%; N, 13.14%. Found: C, 67.90%; H, 4.51%; N, 12.71%.

#### 3.1.3. 3-{4-[1-Acetyl-5-(4-hydroxy-3-methoxy-phenyl)-1*H*-pyrazol-3-yl]-phenyl}-1*H*-quinazolin-2,4-dione 3

Reaction of chalcone 1 (0.414 g, 1 mmol) and hydrazine hydrate (0.12 mL, 2 mmol) in glacial acetic acid (10 mL) was heated under reflux for 18 h. The precipitate that formed was collected through filtration, dried, and recrystallized using benzene/ethanol to give the desired product 3 as white crystals. Yield: 78% (0.36 gm); M.P. 280–282 °C. FT-IR (KBr, *ν*, cm^−1^): 3473 (OH), 3233 (NH), 1727 and 1673 (C=O stretches); ^1^H-NMR (400 MHz, DMSO-d6, *δ*, ppm): 1.90 (s, 3H, CH_3_), 3.98 (s, 3H, OCH_3_), 7.23–8.08 (m, 12H, Ar–H + CH), 11.62 (s, 1H, NH); ^13^C-NMR (100 MHz, DMSO, *δ*, ppm): 26, 55, 102, 114, 115, 122, 127, 128, 129, 135, 136, 139, 140, 149, 162, 169; MS: *m/z* = 468 [M^+^]. Elemental analysis calculated for C_26_H_20_N_4_O_5_: C, 66.66%; H, 4.30%; N, 11.96%. Found: C, 66.75%; H, 4.41%; N, 11.75%.

#### 3.1.4. 3-{4-[5-(4-Hydroxy-3-methoxy-phenyl)-1-(2,2,2-trifluoro-acetyl)-1*H*-pyrazol-3-yl]-phenyl}-1*H*-quinazolin-2,4-dione 4

A mixture of chalcone 1 (0.414 g, 1 mmol) and hydrazine hydrate (0.12 mL, 2 mmol) in trifluoroacetic acid (10 mL) was heated under reflux for 24 h (monitored by TLC). The resulting precipitate was isolated by filtration, dried, and recrystallized from benzene/ethanol to yield product 4 as gray crystals. Yield: 75% (0.39 g); M.P. 292–294 °C. FT-IR (KBr, *ν*, cm^−1^): 3438 (OH), 3233 (NH), 1727 and 1666 (C=O stretches); ^1^H-NMR (400 MHz, DMSO-d6, *δ*, ppm): 3.95 (s, 3H, OCH_3_), 7.20–8.08 (m, 12H, Ar–H + CH), 11.58 (s, 1H, NH); MS: *m/z* = 522 [M^+^]. Elemental analysis calculated for C_26_H_17_F_3_N_4_O_5_: C, 59.77%; H, 3.28%; F, 10.91%; N, 10.72%. Found: C, 59.88%; H, 3.37%; F, 10.99%; N, 10.60%.

#### 3.1.5. 3-{4-[5-(4-Hydroxy-3-methoxy-phenyl)-1-phenyl-1*H*-pyrazol-3-yl]-phenyl}-1*H*-quinazolin-2,4-dione 5

A mixture of chalcone 1 (0.414 g, 1 mmol) and phenylhydrazine (0.11 g, 1 mmol) in absolute ethanol (15 mL) with a few drops of concentrated hydrochloric acid as an acidic catalyst was heated under reflux for 24 h (monitored by TLC). The resulting precipitate was isolated by filtration, dried, and recrystallized from benzene/ethanol to afford compound 5 as white crystals. Yield: 81% (0.4 g); M.P. 250–252 °C. FT-IR (KBr, *ν*, cm^−1^): 3438 (OH), 3301 (NH), 1720 and 1652 (C=O stretches); ^1^H-NMR (400 MHz, DMSO-*d*6, *δ*, ppm): 3.90 (s, 3H, OCH_3_), 6.78–8.08 (m, 17H, Ar–H + CH), 11.59 (s, 1H, NH); ^13^C-NMR (100 MHz, DMSO, *δ*, ppm): 55, 100, 112, 114, 115, 118, 122, 125, 127, 128, 129, 135, 139, 140, 162; MS: *m/z* = 502 [M^+^]. Elemental analysis calculated for C_30_H_22_N_4_O_4_: C, 71.70%; H, 4.41%; N, 11.15%. Found: C, 71.81%; H, 4.52%; N, 11.01%.

#### 3.1.6. 3-{4-[1-(2,4-Dinitro-phenyl)-5-(4-hydroxy-3-methoxy-phenyl)-1*H*-pyrazol-3-yl]-phenyl}-1*H*-quinazolin-2,4-dione 6

A mixture of chalcone 1 (0.414 g, 1 mmol) and 2,4-dinitrophenylhydrazine (0.198 g, 1 mmol) in absolute ethanol (15 mL), catalyzed by a few drops of concentrated hydrochloric acid, was heated under reflux for 36 h. The reaction mixture was cooled and acidified by pouring onto an ice-water mixture containing hydrochloric acid. The resulting solid, which precipitated was isolated by filtration, dried, and recrystallized from benzene/ethanol to afford compound 6 as orange crystals. Yield: 70% (0.41 g); M.P. 200–202 °C. FT-IR (KBr, *ν*, cm^−1^): 3450 (OH), 3233 (NH), 1727 and 1673 (C=O stretches), 1495 (NO_2_ asymmetric stretch), 1392 (NO_2_ symmetric stretch); ^1^H-NMR (400 MHz, DMSO-*d*6, *δ*, ppm): 3.90 (s, 3H, OCH_3_), 7.23–8.81 (m, 15H, Ar–H + CH), 11.57 (s, 1H, NH); MS: *m/z* = 592 [M^+^]. Elemental analysis calculated for C_30_H_20_N_6_O_8_: C, 60.81%; H, 3.40%; N, 14.18%. Found: C, 60.90%; H, 3.49%; N, 14.02%.

#### 3.1.7. 3-[4-(2,4-Dioxo-1,4-dihydro-2*H*-quinazolin-3-yl)-phenyl]-5-(4-hydroxy-3-methoxy-phenyl)-pyrazole-1-carboxylic acid amide 7

A solution of chalcone 1 (0.414 g, 1 mmol) and semicarbazide hydrochloride (0.11 g, 1 mmol) in absolute ethanol (15 mL) in presence of catalytic quantity of NaOH (10%) as a basic catalyst (1 mL) was subjected to reflux for 20 h. The reaction mixture was cooled and acidified by pouring onto an ice-water mixture containing hydrochloric acid. The resulting solid, which precipitated was isolated by filtration, dried, and recrystallized from benzene/ethanol to afford compound 7 as white crystals. Yield: 80% (0.37 g); M.P. 320–322 °C. FT-IR (KBr, *ν*, cm^−1^): 3459 (OH), 3201 (NH), 1679 (C=O stretches); ^1^H-NMR (400 MHz, DMSO-*d*_6_, *δ*, ppm): 3.99 (s, 3H, OCH_3_), 5.60 (s, 2H, NH_2_), 6.98–8.01 (m, 12H, Ar–H + CH), 11.57 (s, 1H, NH); MS: *m/z* = 469 [M^+^]. Elemental analysis calculated for C_25_H_19_N_5_O_5_: C, 63.96%; H, 4.08%; N, 14.92%. Found: C, 64.10%; H, 4.18%; N, 14.72%.

#### 3.1.8. 3-[4-(2,4-Dioxo-1,4-dihydro-2*H*-quinazolin-3-yl)-phenyl]-5-(4-hydroxy-3-methoxy-phenyl)-pyrazole-1-carbothioic acid amide 8

Compound 8 was synthesized by refluxing a mixture of chalcone 1 (0.414 g, 1 mmol) and thiosemicarbazide (0.093 g, 1 mmol) in absolute ethanol (15 mL) in presence of catalytic quantity of NaOH (10%) as a basic catalyst (1 mL) for 20 h. The reaction mixture was cooled and acidified by pouring onto an ice-water mixture containing hydrochloric acid. The resulting solid, which precipitated was isolated by filtration, dried, and recrystallized from benzene/ethanol to yield the target compound as white crystals. Yield: 70% (0.33 g); M.P. 230–232 °C. FT-IR (KBr, *ν*, cm^−1^): 3384 (OH), 3247 (NH), 1707 and 1659 (C=O stretches); ^1^H-NMR (400 MHz, DMSO-d6, *δ*, ppm): 3.89 (s, 3H, OCH_3_), 5.02 (s, 2H, NH_2_), 7.23–8.32 (m, 12H, Ar–H + CH), 11.60 (s, 1H, NH); ^13^C-NMR (100 MHz, DMSO, *δ*, ppm): 55, 102, 115, 117, 122, 125, 126, 128, 133, 136, 139, 144, 152, 155, 157, 164, 174; MS: *m/z* = 485 [M^+^]. Elemental analysis calculated for C_25_H_19_N_5_O_4_S: C, 61.85%; H, 3.94%; N, 14.42%; S, 6.60%. Found: C, 61.97%; H, 3.99%; N, 14.25%; S, 6.81%.

#### 3.1.9. 3-[4-(2,4-Dioxo-1,4-dihydro-2*H*-quinazolin-3-yl)-phenyl]-5-(4-hydroxy-3-methoxy-phenyl)-pyrazole-1-carbothioic acid hydrazide 9

Compound 9 was synthesized *via* the reflux of chalcone 1 (0.414 g, 1.0 mmol) with thiocarbohydrazide (0.106 g, 1.0 mmol) in absolute ethanol (15 mL). The reaction was catalyzed by a catalytic amount of NaOH (1 mL of a 10% solution). After 24 h of reflux, the mixture was cooled and acidified by pouring onto an ice-water mixture containing hydrochloric acid and the resulting precipitate was collected by filtration, dried, and recrystallized from benzene/ethanol to afford the title compound as white crystals. Yield: 75% (0.37 g); M.P. 292–294 °C. FT-IR (KBr, *ν*, cm^−1^): 3438 (OH), 3260 (NH), 1707 and 1652 (C=O stretches); ^1^H-NMR (400 MHz, DMSO-*d*_6_, *δ*, ppm): 3.85 (s, 3H, OCH_3_), 5.02 (s, 2H, NH_2_), 6.02 (s, 1H, NH-CS), 7.24–8.07 (m, 12H, Ar–H + CH), 11.50 (s, 1H, NH); MS: *m/z* = 500 [M^+^]. Elemental analysis calculated for C_25_H_20_N_6_O_4_S: C, 59.99%; H, 4.03%; N, 16.79%; S, 6.41%. Found: C, 60.12%; H, 4.17%; N, 16.65%; S, 6.52%.

#### 3.1.10. 3-[4-(2,4-Dioxo-1,4-dihydro-2*H*-quinazolin-3-yl)-phenyl]-5-(4-hydroxy-3-methoxy-phenyl)-pyrazole-1-carboxamidine 10

Compound 10 was synthesized by heating a mixture of chalcone 1 (0.414 g, 1.0 mmol) and aminoguanidine hydrochloride (0.11 g, 1.0 mmol) in absolute ethanol (20 mL) with sodium hydroxide (0.04 g, 1.0 mmol) under reflux for 20 h. The resulting precipitate was collected by filtration and recrystallized from benzene/ethanol to afford the product as pale yellow crystals. Yield: 82% (0.37 g); M.P. 280–282 °C. FT-IR (KBr, *ν*, cm^−1^): 3473 (OH), 3363 (NH), 1734 and 1645 (C=O stretches); ^1^H-NMR (400 MHz, DMSO-*d*_6_, *δ*, ppm): 3.98 (s, 3H, OCH_3_), 5.57 (s, 2H, NH_2_), 5.96 (s, 1H, =NH), 7.20–7.95 (m, 12H, Ar–H + CH), 11.58 (s, 1H, NH); MS: *m/z* = 468 [M^+^]. Elemental analysis for C_25_H_20_N_6_O_4_ calculated: C, 64.10%; H, 4.30%; N, 17.94%.Found: C, 64.22%; H, 4.41%; N, 17.72%.

#### 3.1.11. 3-{4-[5-(4-Hydroxy-3-methoxy-phenyl)-isoxazol-3-yl]-phenyl}-1*H*-quinazolin-2,4-dione 11

Isoxazoline derivative 11 was synthesized by refluxing a mixture of chalcone 1 (0.414 g, 1.0 mmol) and hydroxylamine hydrochloride (0.08 g, 1.2 mmol) in pyridine (15 mL) for 24 h. Upon completion, the reaction mixture was cooled and acidified by pouring onto an ice-water mixture containing hydrochloric acid. The resulting precipitate was collected by filtration, dried, and recrystallized from benzene/ethanol to yield compound 11 as white crystals. Yield: 72% (0.30 g); M.P. 298–300 °C. FT-IR (KBr, *ν*, cm^−1^): 3288 (NH), 1714 and 1652 (C=O stretches); ^1^H-NMR (400 MHz, DMSO-d6, *δ*, ppm): 3.90 (s, 3H, OCH_3_), 7.22–8.08 (m, 12H, Ar–H + CH), 11.58 (s, 1H, NH); MS: *m/z* = 427 [M^+^]. Elemental analysis for C_24_H_17_N_3_O_5_ calculated: C, 67.44%; H, 4.01%; N, 9.83%. Found: C, 67.55%; H, 4.21%; N, 9.75%.

#### 3.1.12. 3-{4-[6-(4-Hydroxy-3-methoxy-phenyl)-2-oxo-1,2-dihydro-pyrimidin-4-yl]-phenyl}-1*H*-quinazolin-2,4-dione 12

A stirred solution of chalcone 1 (0.414 g, 1.0 mmol) and urea (0.056 g, 1.0 mmol) in absolute ethanol (20 mL) was treated with sodium hydroxide (0.04 g, 1.0 mmol). The reaction mixture was heated under reflux for 20 h. Upon cooling, the mixture was acidified by pouring onto an ice-water/HCl mixture. The resulting yellow precipitate was collected by filtration, dried, and recrystallized from benzene/ethanol to afford compound 12 as yellow crystals. Yield: 80% (0.36 g); M.P. 288–290 °C. FT-IR (KBr, *ν*, cm^−1^): 3452 (OH), 3192 (NH), 1720 and 1673 (C=O stretches); ^1^H-NMR (400 MHz, DMSO-*d*_6_, *δ*, ppm): 3.89 (s, 3H, OCH_3_), 5.18 (s, 1H, NH), 7.23–8.08 (m, 12H, Ar–H + CH), 11.63 (s, 1H, NH); MS: *m/z* = 454 [M^+^]. Elemental analysis calculated for C_25_H_18_N_4_O_5_: C, 66.08%; H, 3.99%; N, 12.33%. Found: C, 66.15%; H, 4.09%; N, 12.21%.

#### 3.1.13. 3-{4-[6-(4-Hydroxy-3-methoxy-phenyl)-2-thioxo-1,2-dihydro-pyrimidin-4-yl]-phenyl}-1*H*-quinazolin-2,4-dione 13

Compound 13 was synthesized by refluxing a mixture of chalcone 1 (0.414 g, 1.0 mmol), thiourea (0.076 g, 1.0 mmol), and sodium hydroxide (0.04 g, 1.0 mmol) in absolute ethanol (20 mL) for 24 h. The resulting precipitate was isolated by filtration, dried, and recrystallized from benzene/ethanol to afford the product as yellow crystals. Yield: 77% (0.36 g); M.P. 278–280 °C. FT-IR (KBr, *ν*, cm^−1^): 3452 (OH), 3206 (NH), 1727 and 1666 (C=O stretches); ^1^H-NMR (400 MHz, DMSO-*d*_6_, *δ*, ppm): 3.90 (s, 3H, OCH_3_), 7.22–8.08 (m, 12H, Ar–H + CH), 11.58 (s, 1H, NH); MS: *m/z* = 470 [M^+^]. Elemental analysis calculated for C_25_H_18_N_4_O_4_S: C, 63.82%; H, 3.86%; N, 11.91%; S, 6.81%. Found: C, 63.90%; H, 3.97%; N, 11.75%; S, 6.92%.

#### 3.1.14. 3-{4-[6-(4-Hydroxy-3-methoxy-phenyl)-2-imino-1,2-dihydro-pyrimidin-4-yl]-phenyl}-1*H*-quinazolin-2,4-dione 14

A mixture of chalcone 1 (0.414 g, 1.0 mmol), guanidine hydrochloride (0.095 g, 1.0 mmol), and sodium hydroxide (0.04 g, 1.0 mmol) in absolute ethanol (20 mL) was heated under reflux for 18 h. The resulting precipitate was collected by filtration and recrystallized from benzene/ethanol to yield compound 14 as orange crystals. Yield: 77% (0.34 g); M.P. 260–262 °C. FT-IR (KBr, *ν*, cm^−1^): 3397 (OH), 3200 (NH), 1679 (C=O stretches); ^1^H-NMR (400 MHz, DMSO-*d*_6_, *δ*, ppm): 3.80 (s, 3H, OCH_3_), 6.85–8.01 (m, 14H, Ar–H + CH+2NH), 11.50 (s, 1H, NH); ^13^C-NMR (100 MHz, DMSO, *δ*, ppm): 55, 102, 115, 118, 121, 126, 128, 129, 133, 135, 143, 152, 156, 158, 164, 169; MS: *m/z* = 453 [M^+^]. Elemental analysis calculated for C_25_H_19_N_5_O_4_: C, 66.22%; H, 4.22%; N, 15.44%. Found: C, 66.34%; H, 4.33%; N, 15.28%.

#### 3.1.15. 3-{4-[6-(4-Hydroxy-3-methoxy-phenyl)-2-phenyl-pyrimidin-4-yl]-phenyl}-1*H*-quinazolin-2,4-dione 15

To a stirred solution of chalcone 1 (0.414 g, 1.0 mmol) and benzamidiniumchloride (0.156 g, 1.0 mmol) in absolute ethanol (20 mL) was added sodium hydroxide (0.04 g, 1.0 mmol). The reaction mixture was refluxed for 20 h, cooled, and acidified by pouring onto ice-water containing HCl. The precipitated solid was collected by filtration, dried, and recrystallized from benzene/ethanol to yield compound 15 as yellow crystals. Yield: 82% (0.34 g); M.P. 198–200 °C. FT-IR (KBr, *ν*, cm^−1^): 3486 (OH), 3315 (NH), 1714 and 1673 (C=O stretches); ^1^H-NMR (400 MHz, DMSO-d6, *δ*, ppm): 3.89 (s, 3H, OCH_3_), 7.06–8.39 (m, 17H, Ar–H + CH), 11.58 (s, 1H, NH); ^13^C-NMR (100 MHz, DMSO, *δ*, ppm): 55, 100, 115, 117, 120, 121, 122, 127, 128, 129, 130, 131, 133, 135, 141, 144, 152, 162, 169; MS: *m/z* = 514 [M^+^]. Elemental analysis calculated for C_31_H_22_N_4_O_4_: C, 72.36%; H, 4.31%; N, 10.89%. Found: C, 72.56%; H, 4.41%; N, 10.79%.

### 3.2. Biological evaluation

Minimum inhibitory concentration (MIC) and antimicrobial efficacy of the novel derivatives were estimated against two Gram-negative bacteria (*Escherichia coli* ATCC 25955, *Pseudomonas aeruginosa* ATCC 10145) and two Gram-positive bacteria (*Bacillus subtilis* ATCC 6633 and *Staphylococcus aureus* NRRL B-767). The microbes under study were provided by Al-Azhar University, Egypt. They were cultivated in Mueller Hinton broth at 35 ± 2 °C for 24 h. The antimicrobial activity and MIC were carried out as described by Qader *et al*.^46–48^

### 3.3. *In silico* molecular docking study and ADMET analysis

The crystal structure of glucoseamine-6-phosphate (GlcN-6-P) synthase was retrieved from the RCSB database (PDBID׃1moq).^49^ The structure was prepared by removing water, heteroatoms, and co-crystalized ligand utilizing Discovery Studio 3.5 Client.^50^ All synthesized molecules 1–15, ciprofloxacin, co-crystalized ligand and two references were used as a library in the docking process. The 2D chemical structures of the molecules, ciprofloxacin, co-crystalized ligand and two references were sketched using ChemDraw software 16.0, and then converted to the 3D structures by using Open Babel GUI tool.^51^ The energy minimization for the enzyme file and ligand molecules was performed using CHARMm^52^ and AMBER^53^ Force Fields, respectively. PyRx virtual screening tool^54^ was utilized for the docking approach between the ligand molecules and the target. The 2D and 3D poses of the docked complexes were visualized using Discovery Studio visualizer software. Finally, the pharmacokinetics properties of the newly molecules, ciprofloxacin, co-crystalized ligand and two references were calculated using AdmetSAR, and SwissADME web-based tools. Finally, geometric optimization of the best docked molecules was investigated by a semiempirical modeling with PM6 basis set (MOPAC 2009 software).^55,56^

## 4. Conclusion

Herein, a new series of quinazolin-2,4-diones incorporating vanillin connecting by biologically active five- and/or six-membered nitrogen rings was rationally designed and synthesized employing Claisen–Schmidt condensation and Michael addition reactions. Structural elucidation was validated by means of spectroscopic techniques and elemental analysis. The antibacterial potentials of all molecules were evaluated, showing moderate to good potency, with compounds 2, 5 and 11 performing significant activity comparable to the standard drug ciprofloxacin. *In silico* molecular docking simulations were performed against glucoseamine-6-phosphate (GlcN-6-P) synthase (PDBID׃1moq), and the molecules 2, 5 and 11 formed highly binding affinities towards the target. The presence of electron donating-groups –OH, and –OCH_3_ attached to aromatic and heteroaromatic moieties could improve the antibacterial potentials of the compounds. When taken as a whole, the results exhibited that the molecules 2, 5 and 11 are promising lead compounds for the development of novel and potent GlcN-6-P inhibitors for bacterial treatment. Finally, *in vivo* evaluation of the most active compounds in appropriate animal infection models will provide critical insights into their therapeutic potentials, guiding further lead optimization toward the development of novel and potent antibacterial inhibitors.

## Conflicts of interest

There are no conflicts to declare.

## Supplementary Material

RA-016-D5RA08138F-s001

## Data Availability

The data supporting this article have been included as part of the supplementary information (SI). Supplementary information is available. See DOI: https://doi.org/10.1039/d5ra08138f.
