# Supplementary material for: Vanillin-tethered quinazolin-2,4-dione analogues through five- and/or six-membered nitrogen-containing heterocycles as antibacterial agents: synthesis, biological evaluation and molecular docking study
Source: RSC Adv. 2026 Jan 27;16(7):5726–42. doi: 10.1039/d5ra08138f (PMC12836203; doi:10.1039/d5ra08138f)
Supplement: RA-016-D5RA08138F-s001 [file RA-016-D5RA08138F-s001.pdf]

Supplementary Information (SI) for RSC Advances.
This journal is © The Royal Society of Chemistry 2026

Supplementary file

Vanillin-tethered quinazolin-2,4-dione analogues through five- and/or six- membered nitrogen-containing heterocycles as antibacterial agents: synthesis, biological evaluation and molecular docking study

Aboubakr H. Abdelmonsef[1*], Saleh M. Elnaby[1], Ahmed M. Mosallam[1], Huda R. M. Rashdan[2], Hesham M. Alsoghier[1], Mohamed A. Raslan[3]

Table S1. A table of comparison with the previously published reports[1,2]

	2D Structure/Name	Temperature<br>Color<br>Yield %<br>M.P. °C<br>Crystallization	2D Structure/Name<br>(reported work)	Temperature<br>Color<br>Yield %<br>M.P. °C<br>Crystallization
1	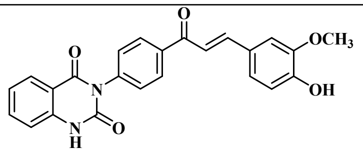<br>3-{4-[3-(4-Hydroxy-3-methoxy-phenyl)-acryloyl]-phenyl}-1H-quinazolin-2,4-dione	stirring 24 hrs<br>(0 to -10 °C)<br>yellow<br>79<br>220-222<br>ethanol	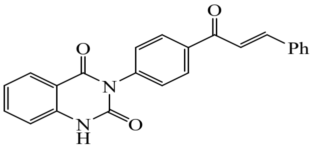<br>3-[4-(3-Phenyl-acryloyl)-phenyl]-1H-quinazolin-2,4-dione	stirring 24 hrs<br>(0 to -10 °C)<br>pale yellow<br>76<br>162-164<br>ethanol
2	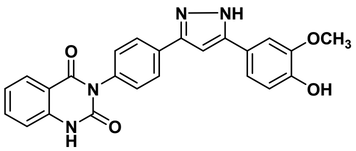<br>3-{4-[5-(4-Hydroxy-3-methoxy-phenyl)-1H-pyrazol-3-yl]-phenyl}-1H-quinazolin-2,4-dione	reflux 18 hrs<br>white<br>80<br>275-277<br>benzene/ethanol	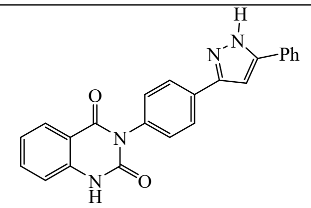<br>3-[4-(5-Phenyl-1H-pyrazol-3-yl)-phenyl]-1H-quinazolin-2,4-dione	reflux 10 hrs<br>white<br>88<br>274-276<br>benzene/ethanol
3	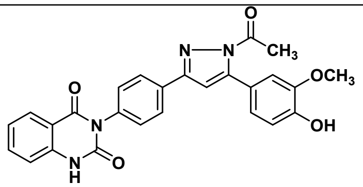<br>3-{4-[1-Acetyl-5-(4-hydroxy-3-methoxy-phenyl)-1H-pyrazol-3-yl]-phenyl}-1H-quinazolin-2,4-dione	reflux 18 hrs<br>white<br>78<br>280-282<br>benzene/ethanol	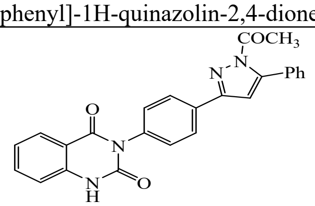<br>3-[4-(1-Acetyl-5-phenyl-1H-pyrazol-3-yl)-phenyl]-1H-quinazolin-2,4-dione	reflux 12 hrs<br>white<br>85<br>256-258<br>ethanol
4	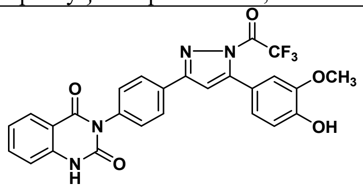<br>3-{4-[5-(4-Hydroxy-3-methoxy-phenyl)-1-(2,2,2-trifluoro-acetyl)-1H-pyrazol-3-yl]-phenyl}-1H-quinazolin-2,4-dione	reflux 24 hrs<br>gray<br>75<br>292-294<br>benzene/ethanol	-	-
5	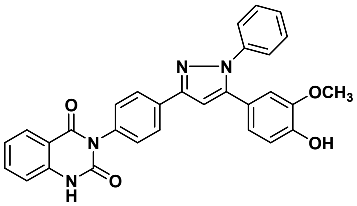<br>3-{4-[5-(4-Hydroxy-3-methoxy-phenyl)-1-phenyl-1H-pyrazol-3-yl]-phenyl}-1H-quinazolin-2,4-dione	reflux 24 hrs<br>white<br>81<br>250-252<br>benzene/ethanol	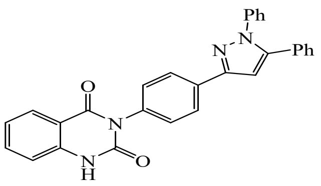<br>3-[4-(1,5-Diphenyl-1H-pyrazol-3-yl)-phenyl]-1H-quinazolin-2,4-dione	reflux 10 hrs<br>yellowish white<br>84<br>144-146<br>benzene/ethanol

**6**	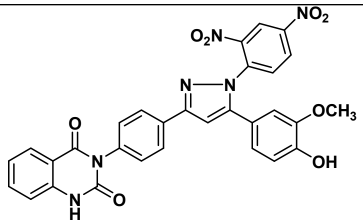<br>3-{4-[1-(2,4-Dinitro-phenyl)-5-(4-hydroxy-3-methoxy-phenyl)-1H-pyrazol-3-yl]-phenyl}-1H-quinazolin-2,4-dione	reflux 36 hrs<br>orange<br>70<br>200-202<br>benzene/ethanol	-	-
**7**	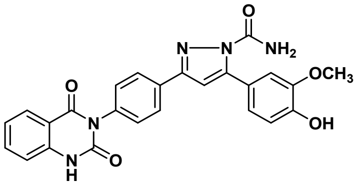<br>3-[4-(2,4-Dioxo-1,4-dihydro-2H-quinazolin-3-yl)-phenyl]-5-(4-hydroxy-3-methoxy-phenyl)-pyrazole-1-carboxylic acid amide	reflux 20 hrs<br>white<br>80<br>320-322<br>benzene/ethanol	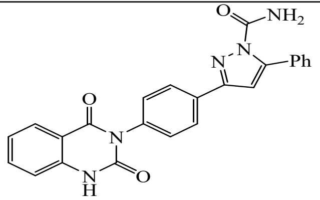<br>3-[4-(2,4-Dioxo-1,4-dihydro-2H-quinazolin-3-yl)-phenyl]-5-phenyl-pyrazole-1-carboxylic acid amide	reflux 10 hrs<br>yellowish white<br>83<br>180-182<br>benzene/ethanol
**8**	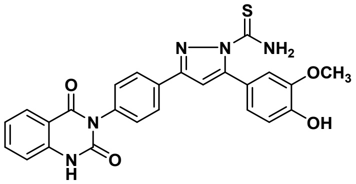<br>3-[4-(2,4-Dioxo-1,4-dihydro-2H-quinazolin-3-yl)-phenyl]-5-(4-hydroxy-3-methoxy-phenyl)-pyrazole-1-carbothioic acid amide	reflux 20 hrs<br>white<br>70<br>230-232<br>benzene/ethanol	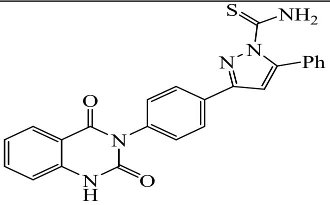<br>3-[4-(2,4-Dioxo-1,4-dihydro-2H-quinazolin-3-yl)-phenyl]-5-phenyl-pyrazole-1-carbothioic acid amide	reflux 10 hrs<br>white<br>84<br>192-194<br>benzene/ethanol
**9**	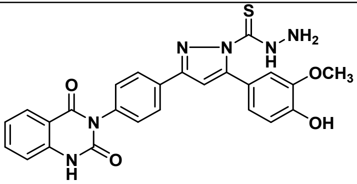<br>3-[4-(2,4-Dioxo-1,4-dihydro-2H-quinazolin-3-yl)-phenyl]-5-(4-hydroxy-3-methoxy-phenyl)-pyrazole-1-carbothioic acid hydrazide	reflux 24 hrs<br>white<br>75<br>292-294<br>benzene/ethanol	-	-
**10**	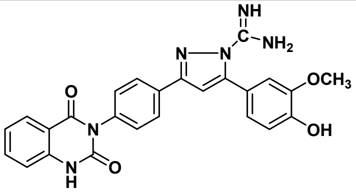<br>3-[4-(2,4-Dioxo-1,4-dihydro-2H-quinazolin-3-yl)-phenyl]-5-(4-hydroxy-3-methoxy-phenyl)-pyrazole-1-carboxamidine	reflux 20 hrs<br>pale yellow<br>82<br>280-282<br>benzene/ethanol	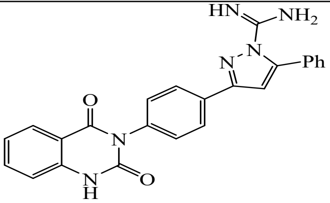<br>3-[4-(2,4-Dioxo-1,4-dihydro-2H-quinazolin-3-yl)-phenyl]-5-phenyl-pyrazole-1-carbox amidine	reflux 8 hrs<br>yellowish white<br>78<br>144-146<br>benzene/ethanol

**11**	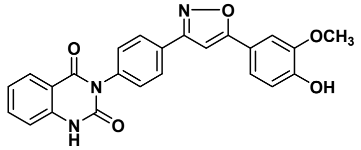<br>3-{4-[5-(4-Hydroxy-3-methoxy-phenyl)-isoxazol-3-yl]-phenyl}-1H-quinazolin-2,4-dione	reflux 24 hrs<br>pale yellow<br>72<br>298-300<br>benzene/ethanol	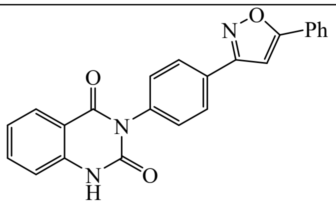<br>3-[4-(5-Phenyl-isoxazol-3-yl)-phenyl]-1H-quinazolin-2,4-dione	reflux 8 hrs<br>white<br>84<br>210-212<br>benzene/ethanol
**12**	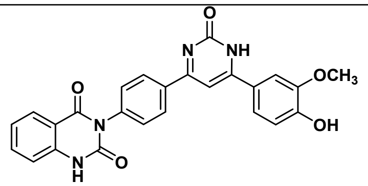<br>3-{4-[6-(4-Hydroxy-3-methoxy-phenyl)-2-oxo-1,2-dihydro-pyrimidin-4-yl]-phenyl}-1H-quinazolin-2,4-dione	reflux 20 hrs<br>yellow<br>72<br>288-290<br>benzene/ethanol	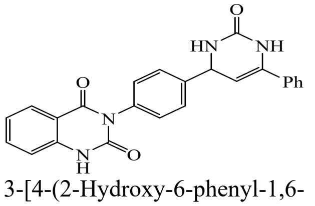<br>3-[4-(2-Hydroxy-6-phenyl-1,6-dihydro-pyrimidin-4-yl)-phenyl]-1H-quinazolin-2,4-dione	reflux 8 hrs<br>white<br>85<br>150-152<br>benzene/ethanol
**13**	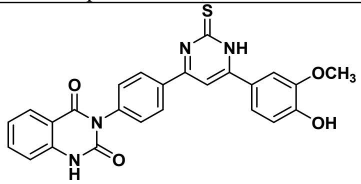<br>3-{4-[6-(4-Hydroxy-3-methoxy-phenyl)-2-thioxo-1,2-dihydro-pyrimidin-4-yl]-phenyl}-1H-quinazolin-2,4-dione	reflux 24 hrs<br>yellow<br>77<br>278-280<br>benzene/ethanol	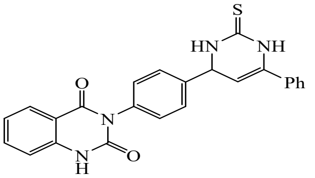<br>3-[4-(2-Mercapto-6-phenyl-1,6-dihydro-pyrimidin-4-yl)-phenyl]-1H-quinazolin-2,4-dione	reflux 10 hrs<br>white<br>82<br>144-146<br>benzene/ethanol
**14**	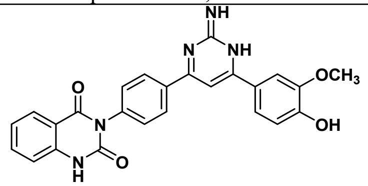<br>3-{4-[6-(4-Hydroxy-3-methoxy-phenyl)-2-imino-1,2-dihydro-pyrimidin-4-yl]-phenyl}-1H-quinazolin-2,4-dione	reflux 18 hrs<br>yellow<br>77<br>260-262<br>benzene/ethanol	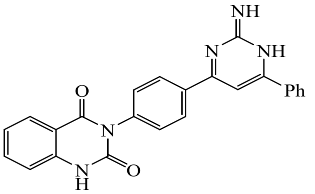<br>3-[4-(2-Amino-6-phenyl-pyrimidin-4-yl)-phenyl]-1H-quinazolin-2,4-dione	reflux 10 hrs<br>yellowish white<br>84<br>152-154<br>benzene/ethanol
**15**	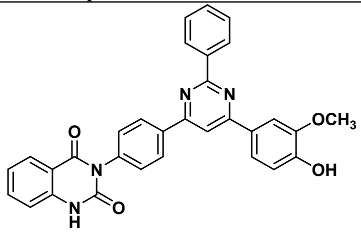<br>3-{4-[6-(4-Hydroxy-3-methoxy-phenyl)-2-phenyl-pyrimidin-4-yl]-phenyl}-1H-quinazolin-2,4-dione	reflux 20 hrs<br>yellow<br>82<br>198-200<br>benzene/ethanol	-	-

(1) Abdelmonsef, A. H.; Mosallam, A. M. Synthesis, in Vitro Biological Evaluation and in Silico Docking Studies of New Quinazolin-2,4-dione Analogues as Possible Anticarcinoma Agents. *J. Heterocycl. Chem.* 2020, *57* (4), 1637–1654. https://doi.org/10.1002/jhet.3889.

(2) El-Naggar, M.; Rashdan, H. R. M.; Abdelmonsef, A. H. Cyclization of Chalcone Derivatives: Design, Synthesis, In Silico Docking Study, and Biological Evaluation of New Quinazolin-2,4-Diones Incorporating Five-, Six-, and Seven-Membered Ring Moieties as Potent Antibacterial

Inhibitors. *ACS Omega* 2023, *8* (30), 27216–27230. https://doi.org/10.1021/acsomega.3c02478.

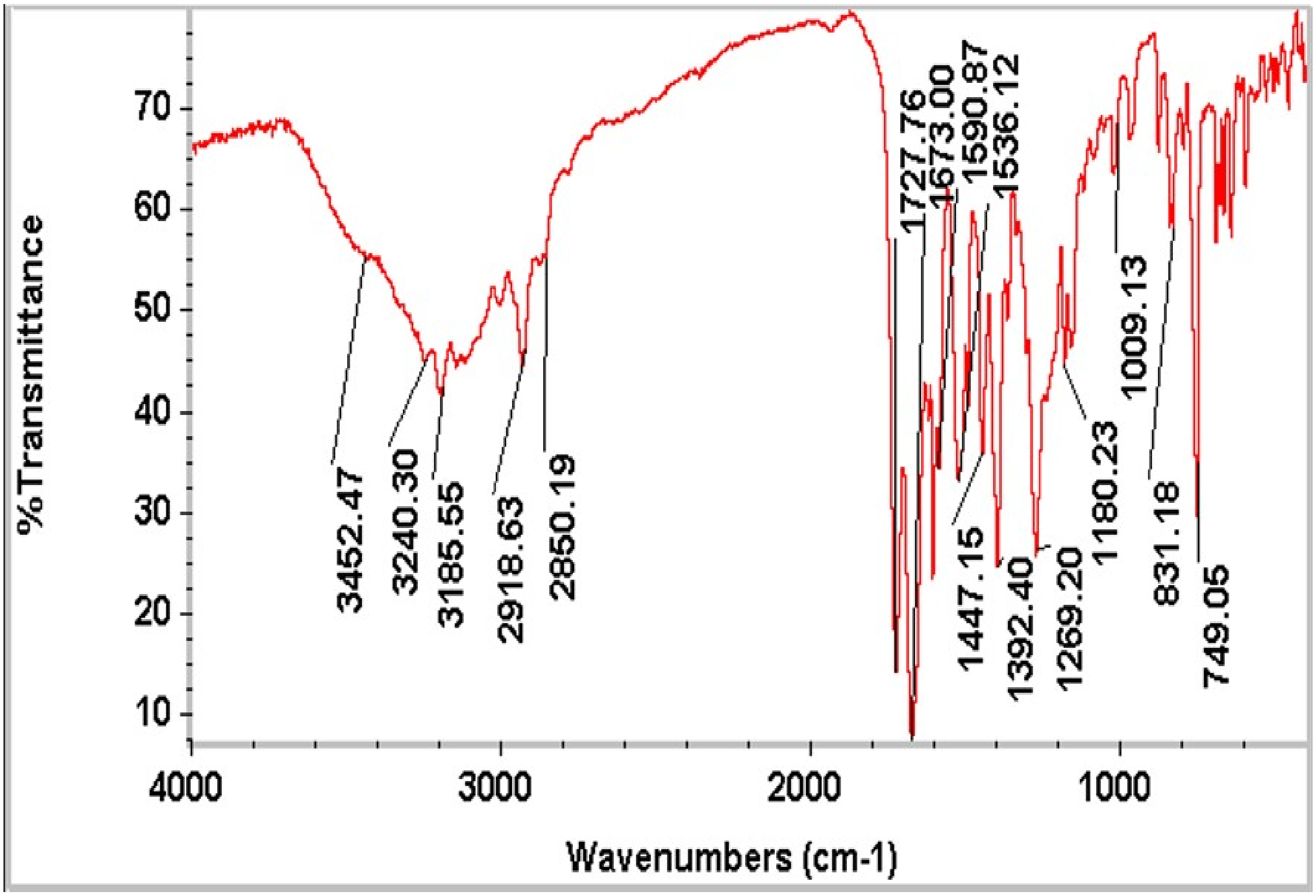

Figure S1. FT-IR spectrum of compound 1

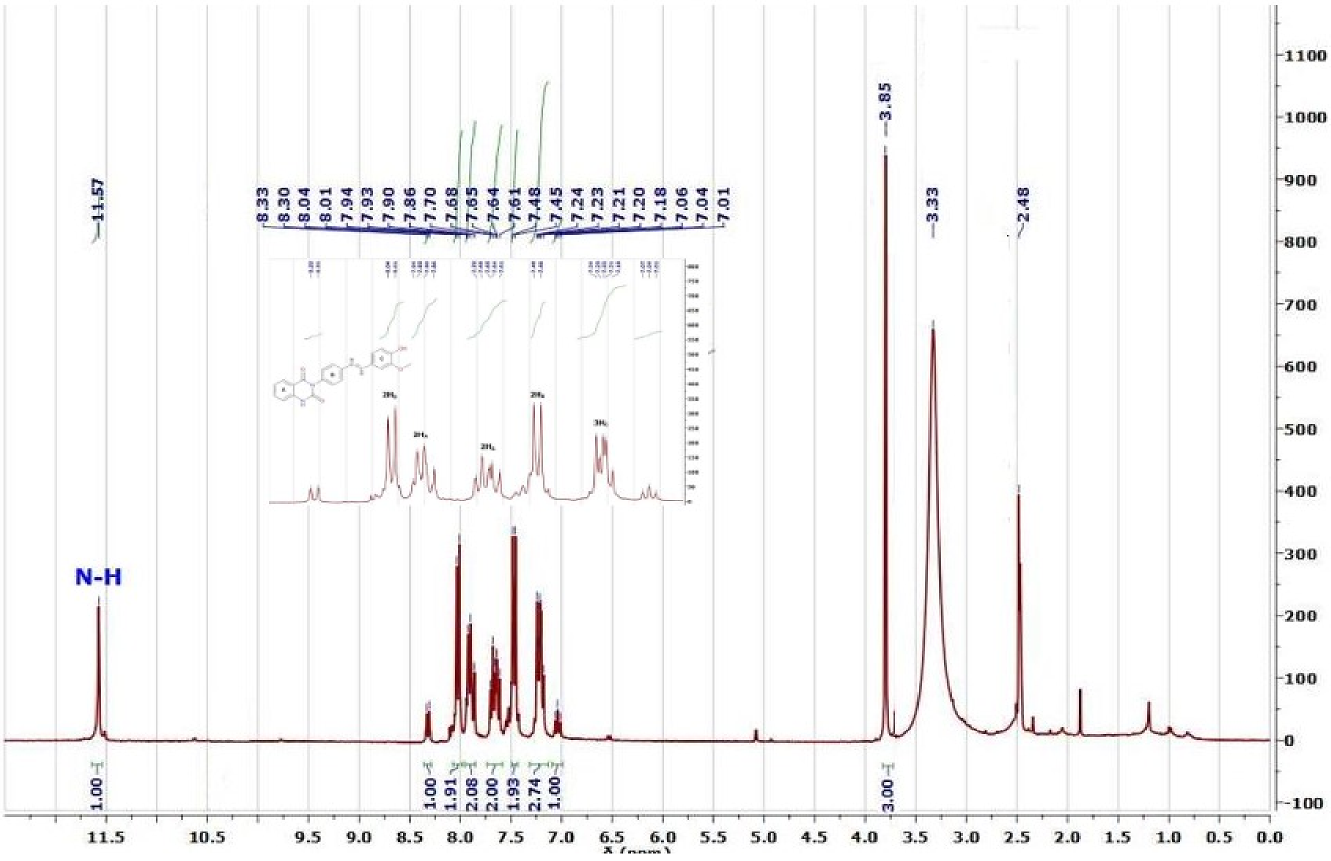

Figure S2. ^{1}HNMR spectrum of compound 1

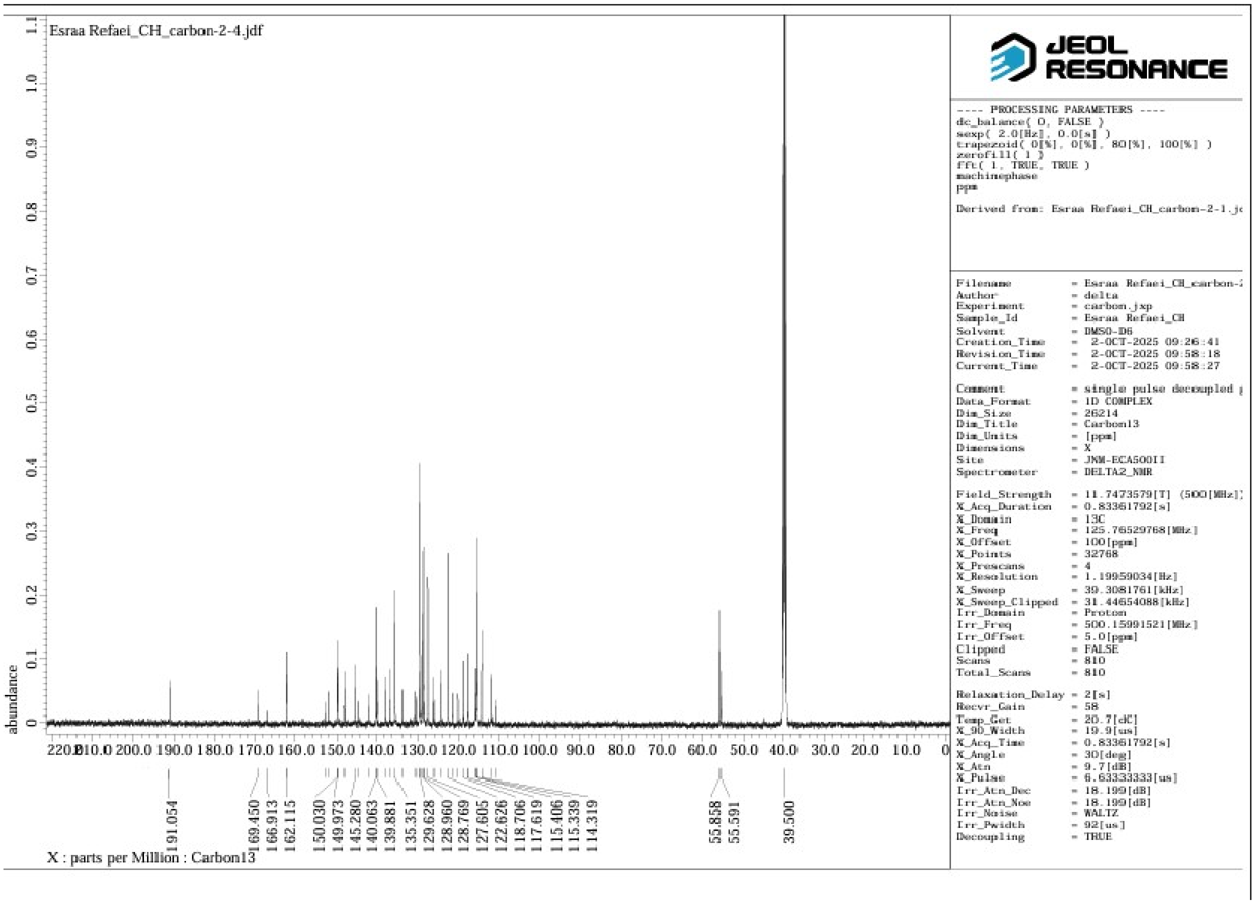

Figure S3. ^{13}CNMR spectrum of compound 1

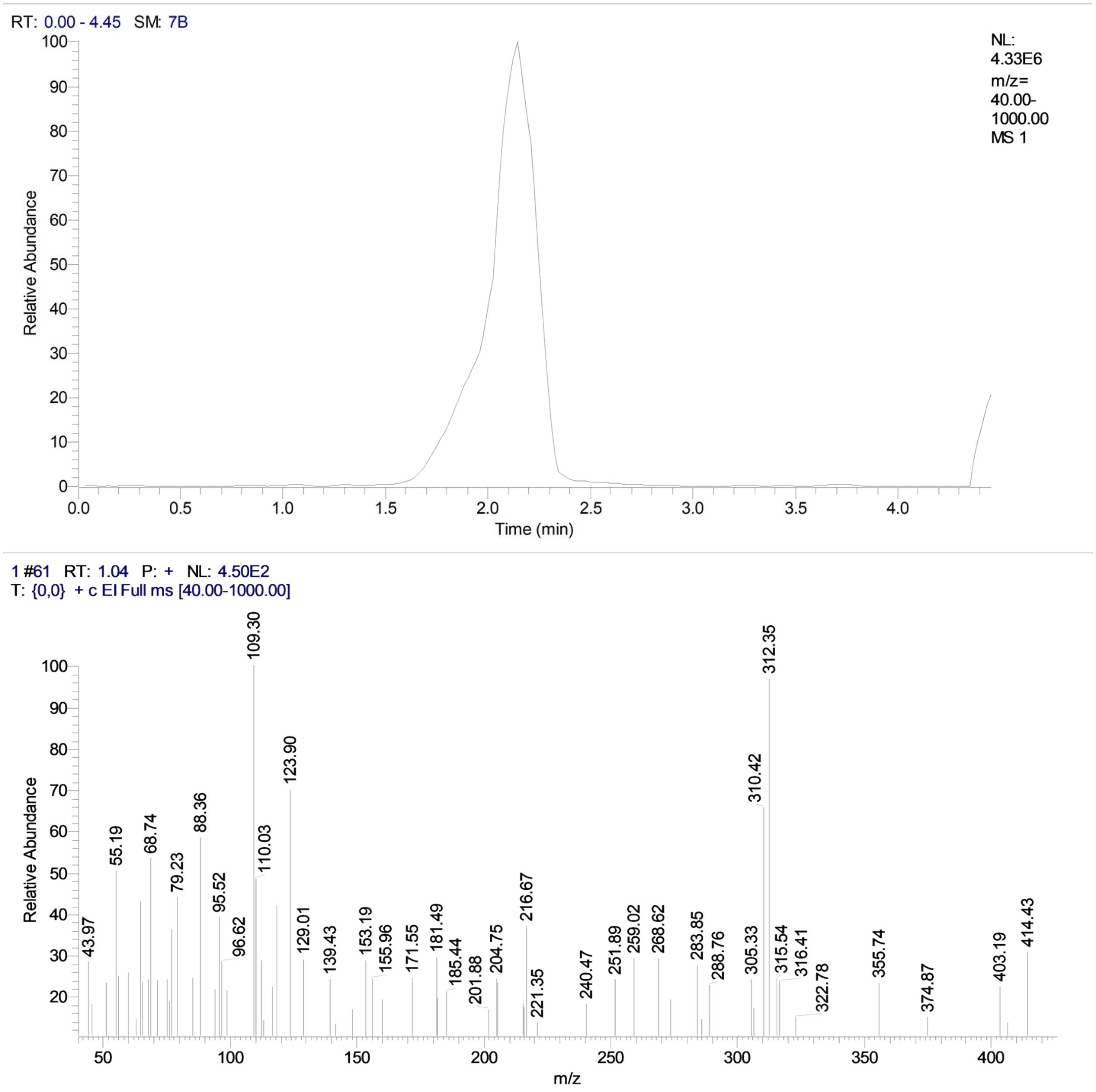

Figure S4. Mass spectrum of compound 1

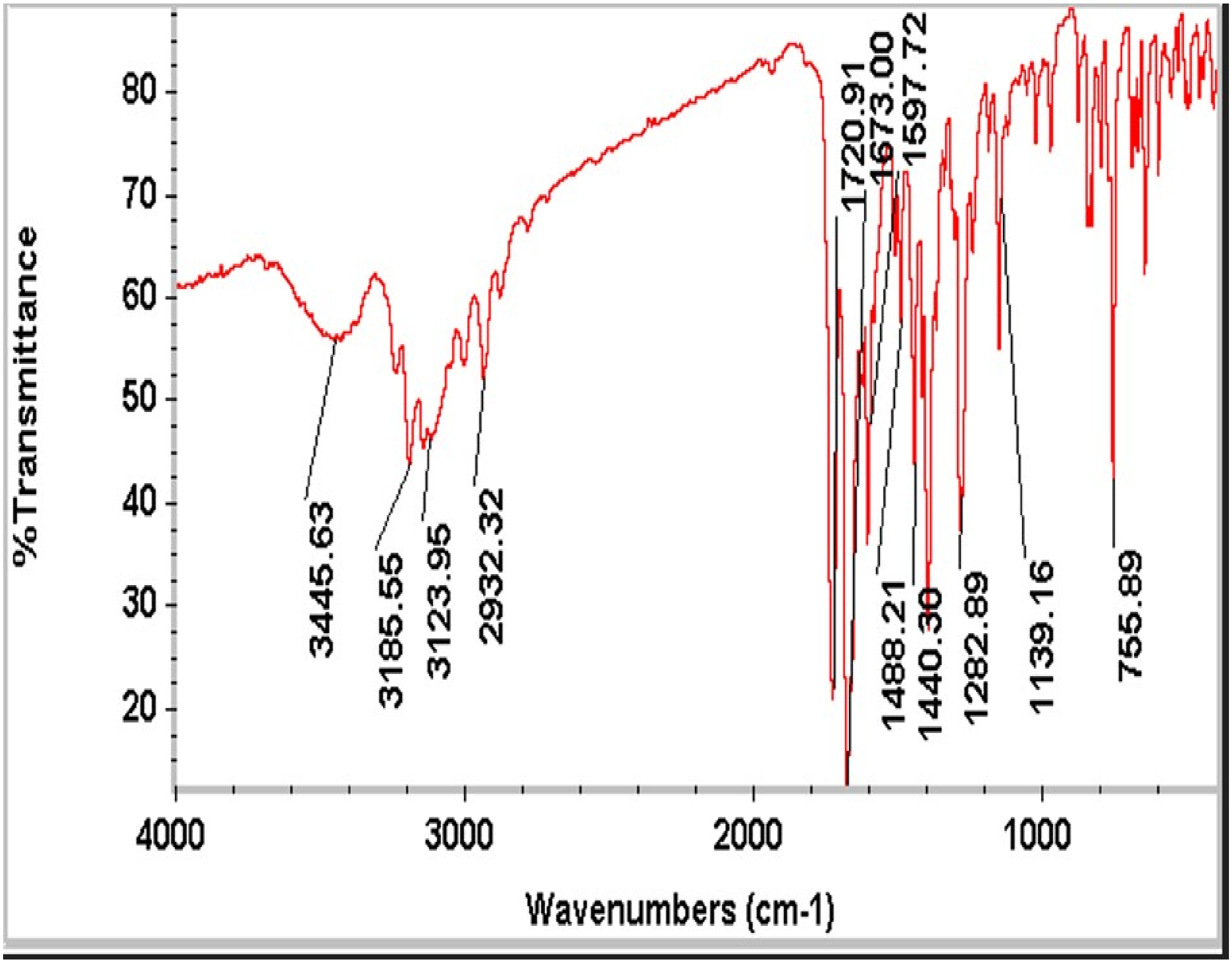

Figure S5. FT-IR spectrum of compound 2

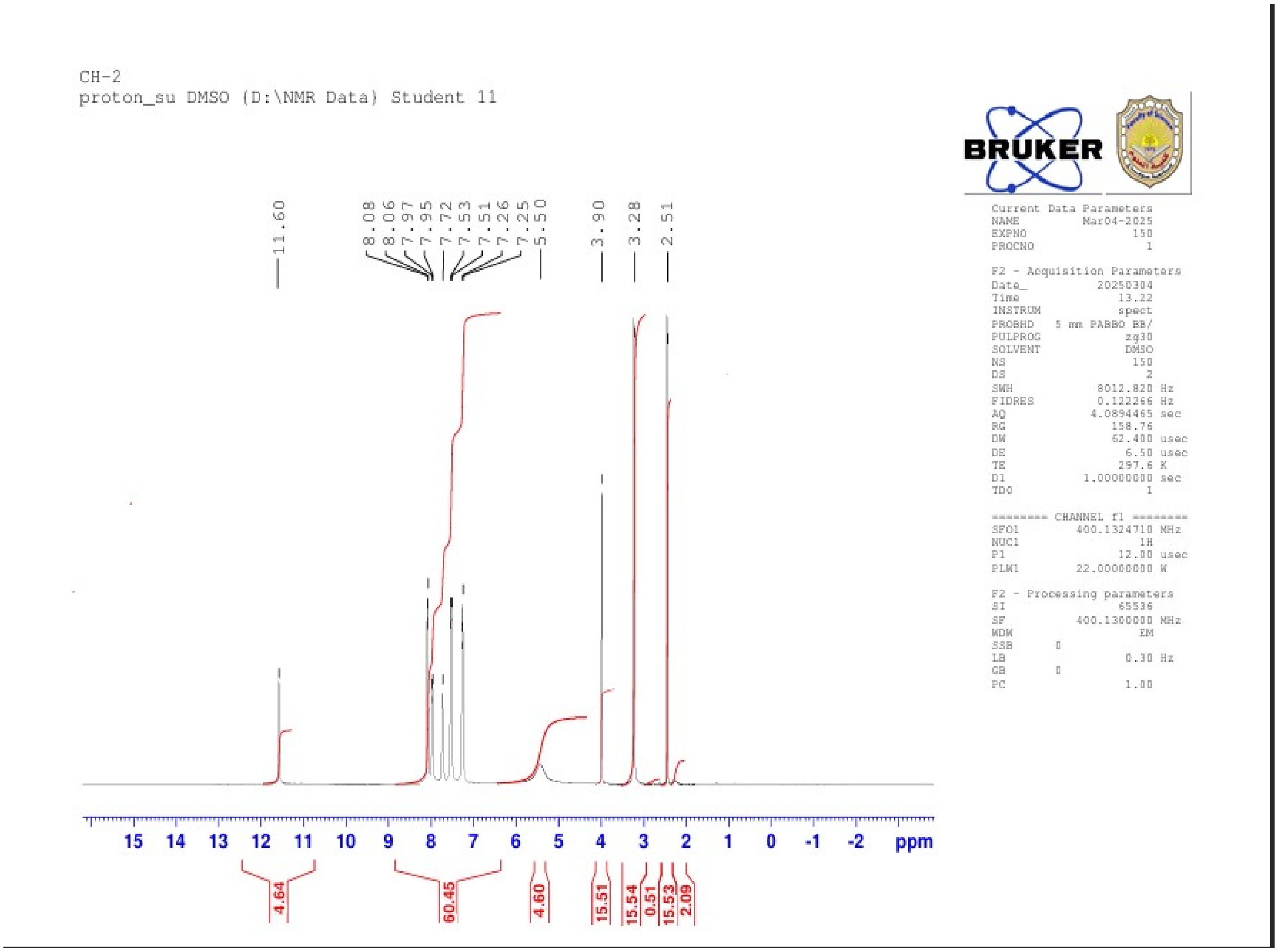

Figure S6. ^{1}HNMR spectrum of compound 2

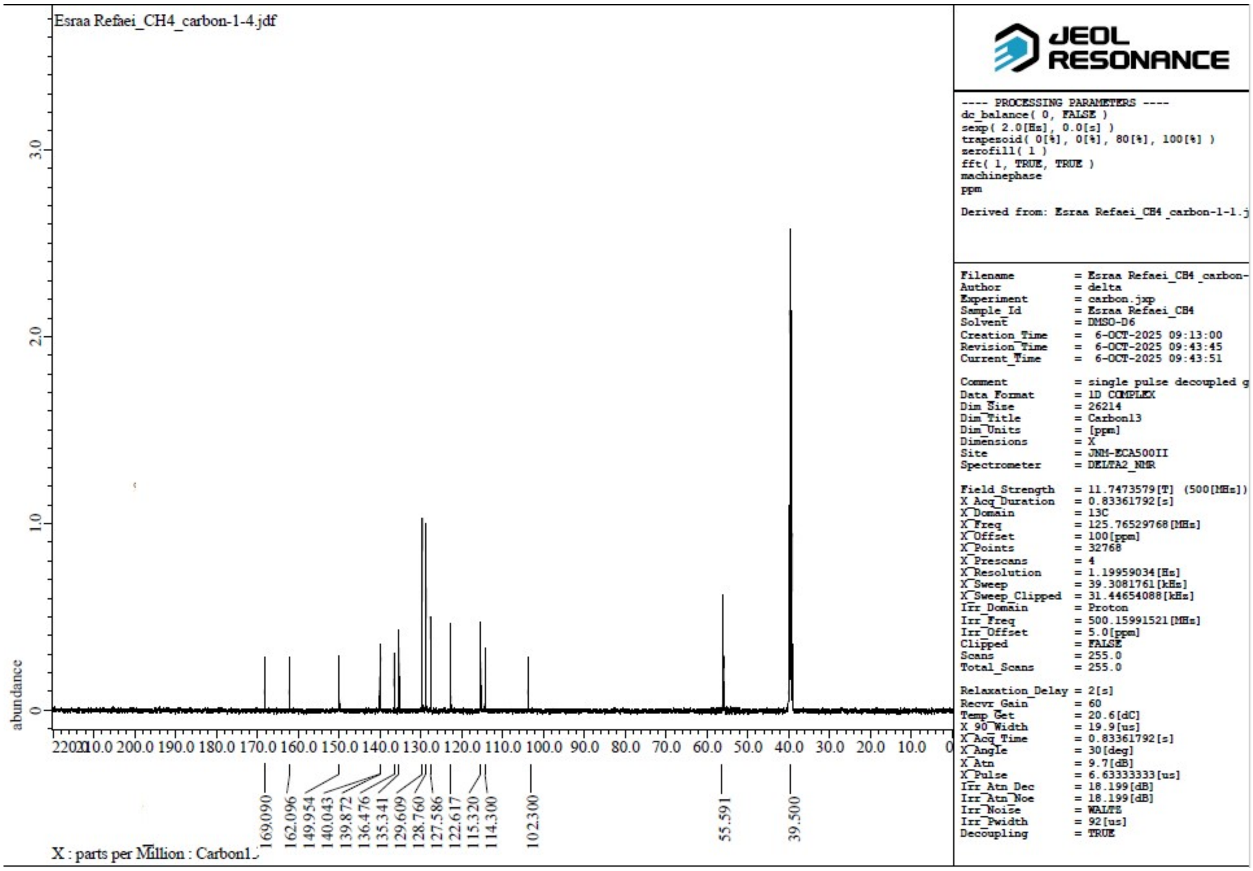

Figure S7. ^{13}CNMR spectrum of compound 2

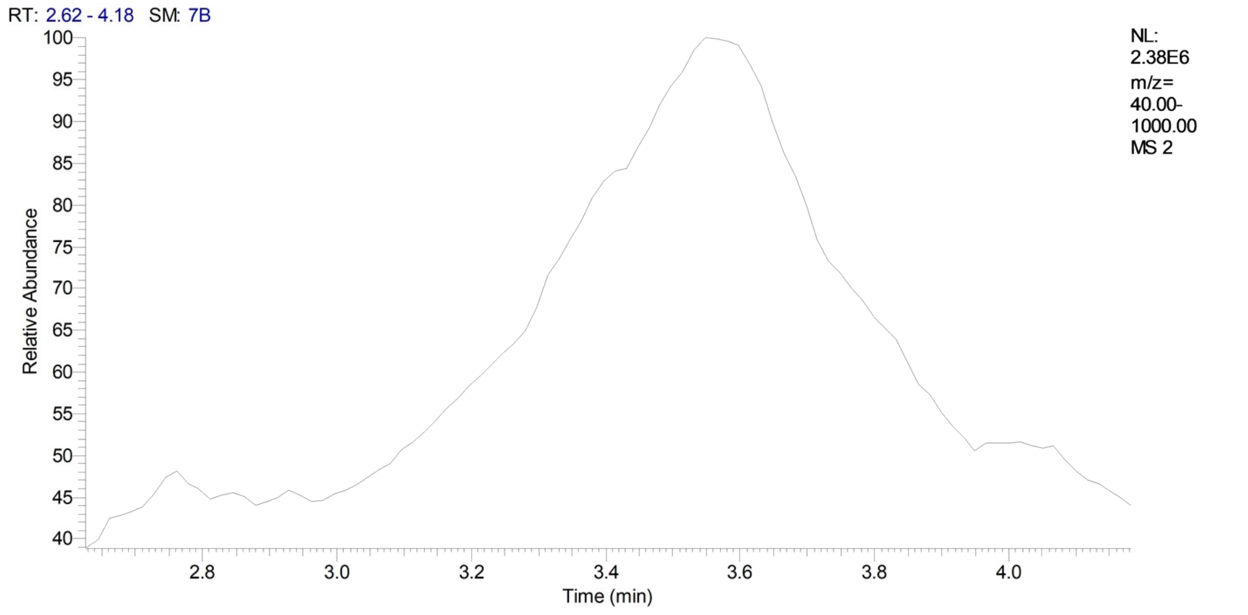

RT: 2.62 - 4.18 SM: 7B
NL:
2.38E6
m/z=
40.00-
1000.00
MS 2
Relative Abundance
Time (min)

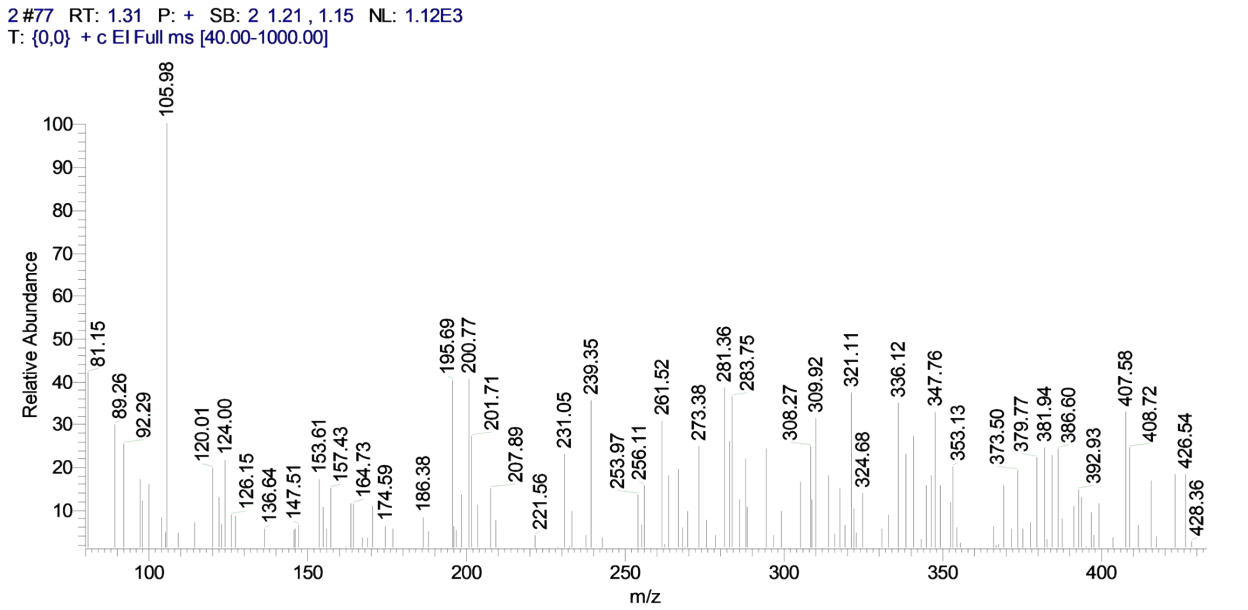

2 #77 RT: 1.31 P: + SB: 2 1.21 , 1.15 NL: 1.12E3
T: {0,0} + c EI Full ms [40.00-1000.00]
Relative Abundance
m/z
105.98
81.15
89.26
92.29
120.01
124.00
126.15
136.64
147.51
153.61
157.43
164.73
174.59
186.38
195.69
200.77
201.71
207.89
221.56
231.05
239.35
253.97
256.11
261.52
273.38
281.36
283.75
308.27
309.92
321.11
324.68
336.12
347.76
353.13
373.50
379.77
381.94
386.60
392.93
407.58
408.72
426.54
428.36

Figure S8. Mass spectrum of compound 2

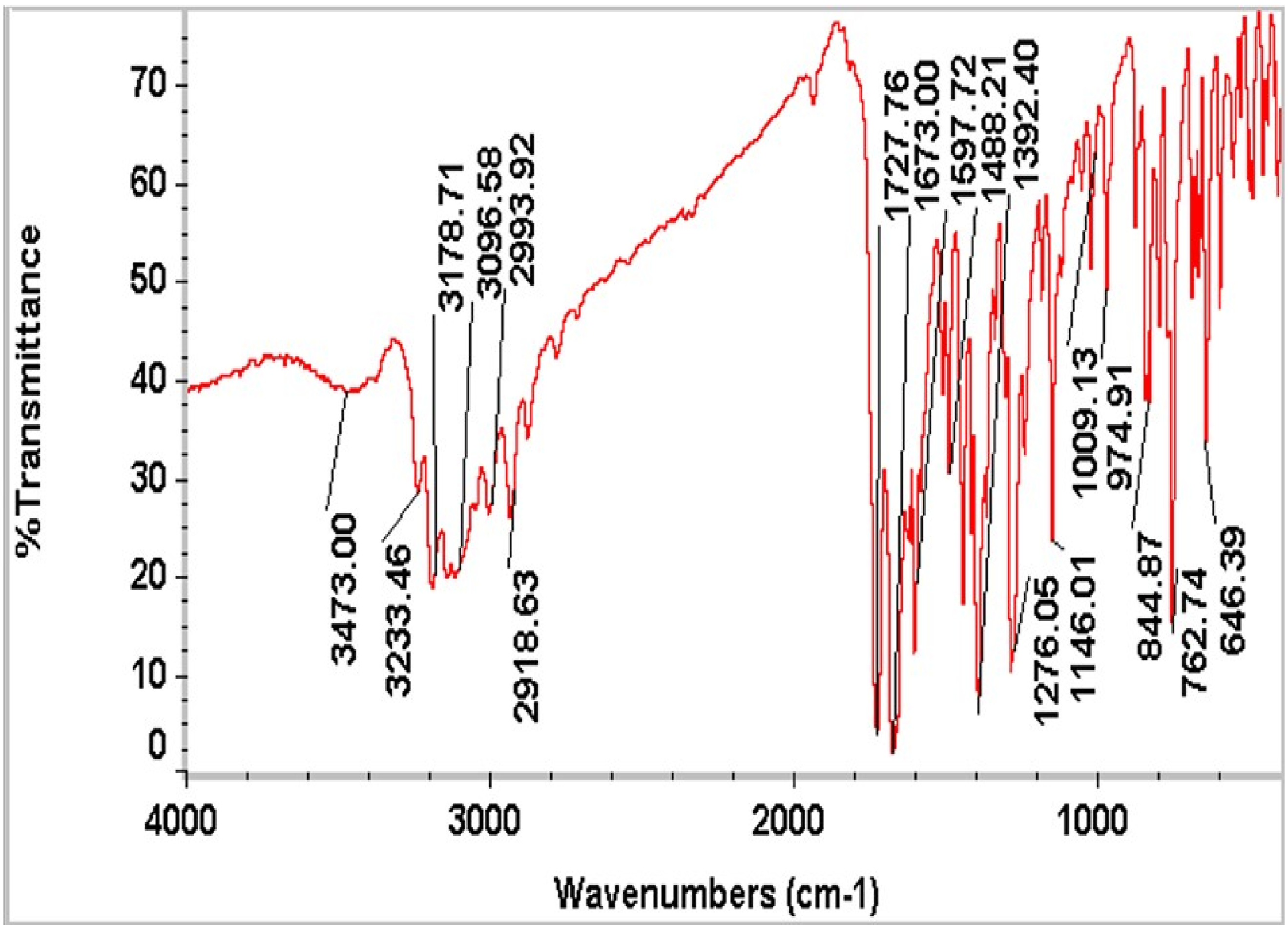

Figure S9. FT-IR spectrum of compound 3

Figure S10. ^{1}HNMR spectrum of compound 3

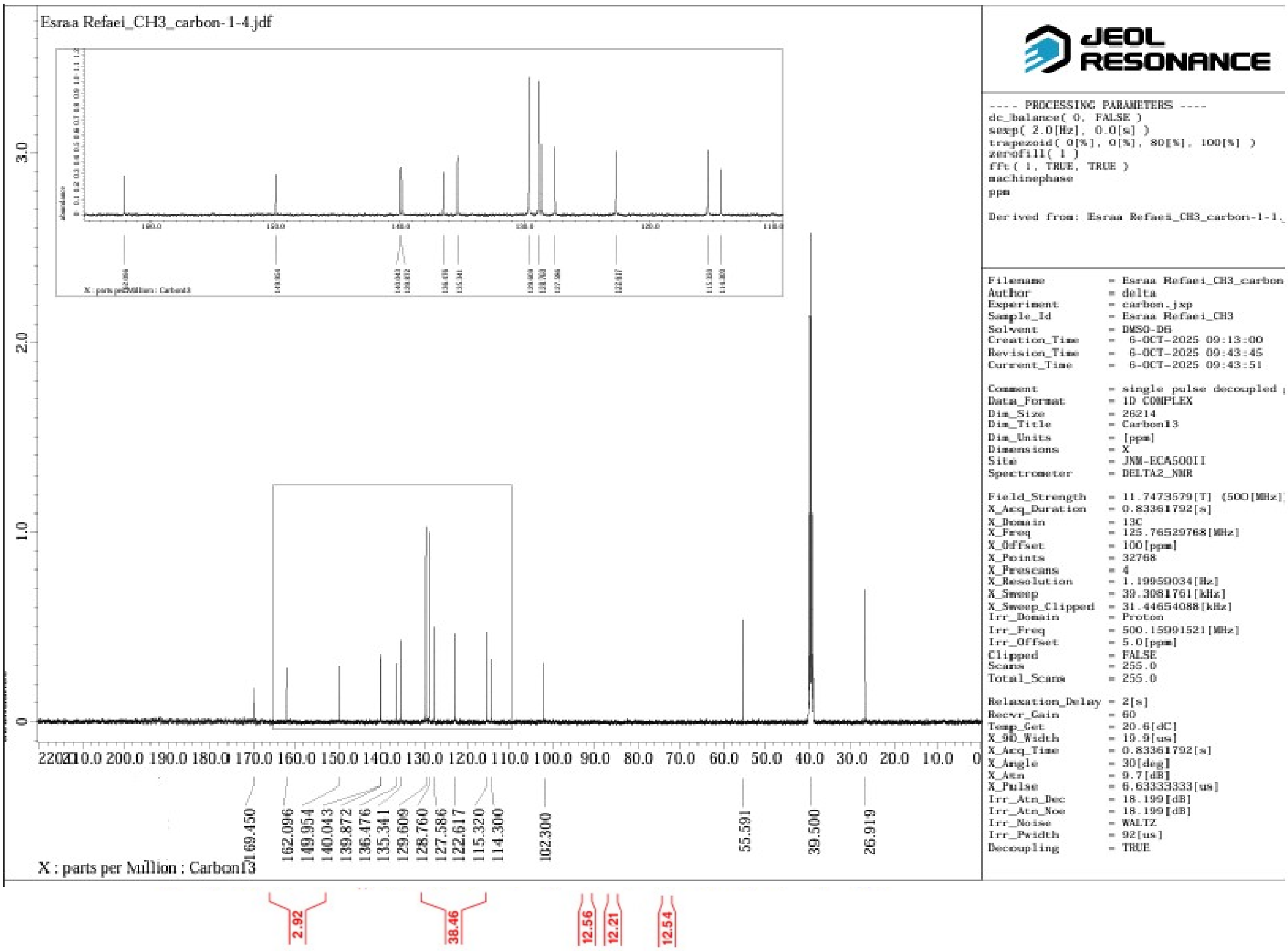

Figure S11. ^{13}CNMR spectrum of compound 3

RT: 0.00 - 4.52 SM: 7B

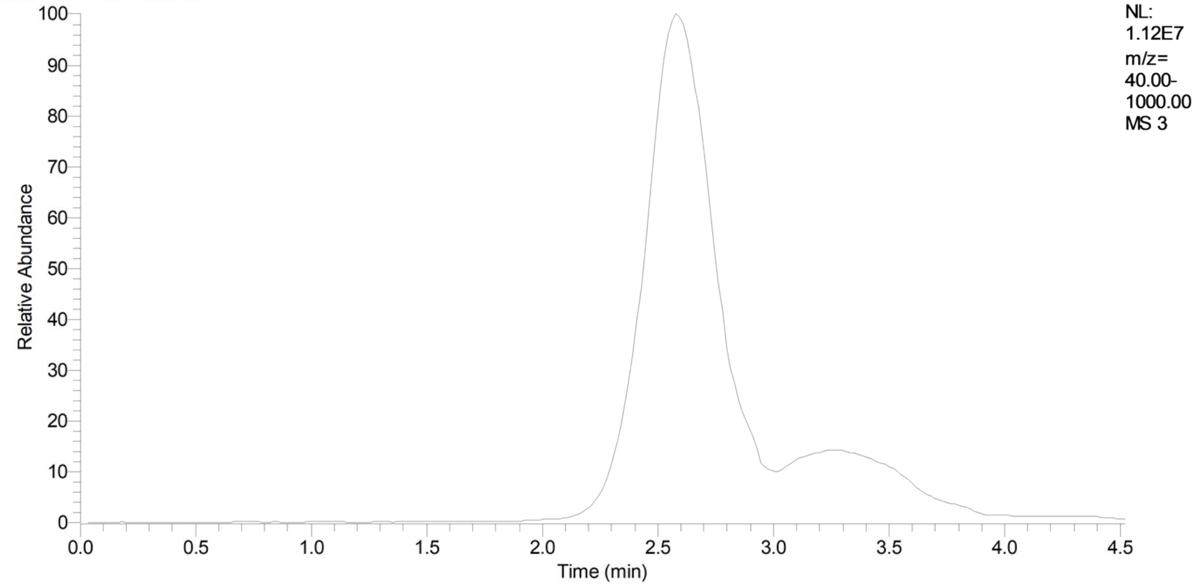

3 #42 RT: 0.72 P: + NL: 6.80E2
T: {0,0} + c EI Full ms [40.00-1000.00]

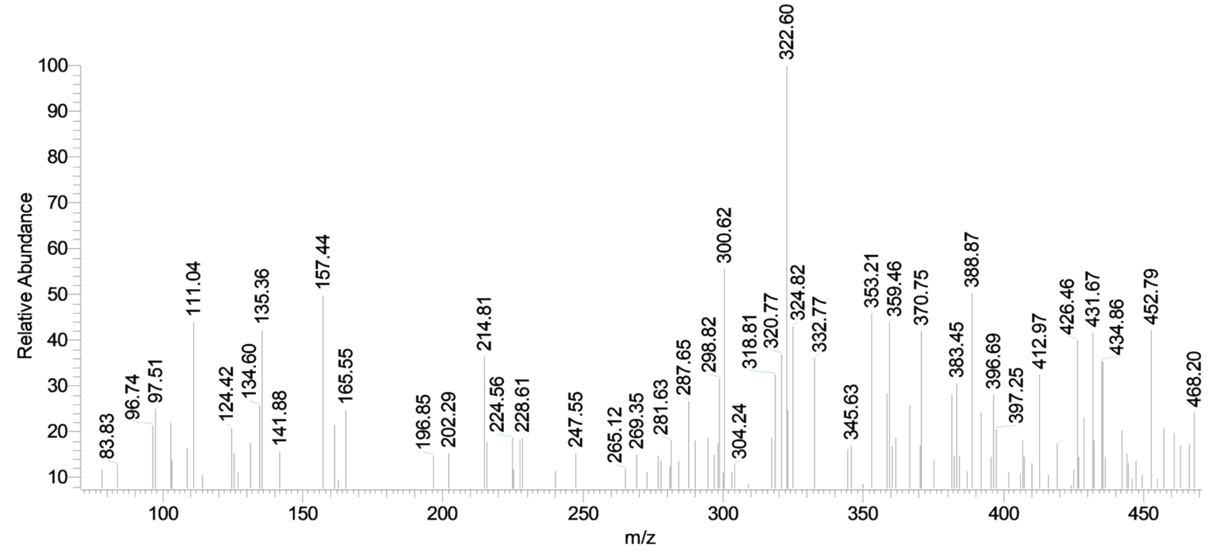

Figure S12. Mass spectrum of compound 3

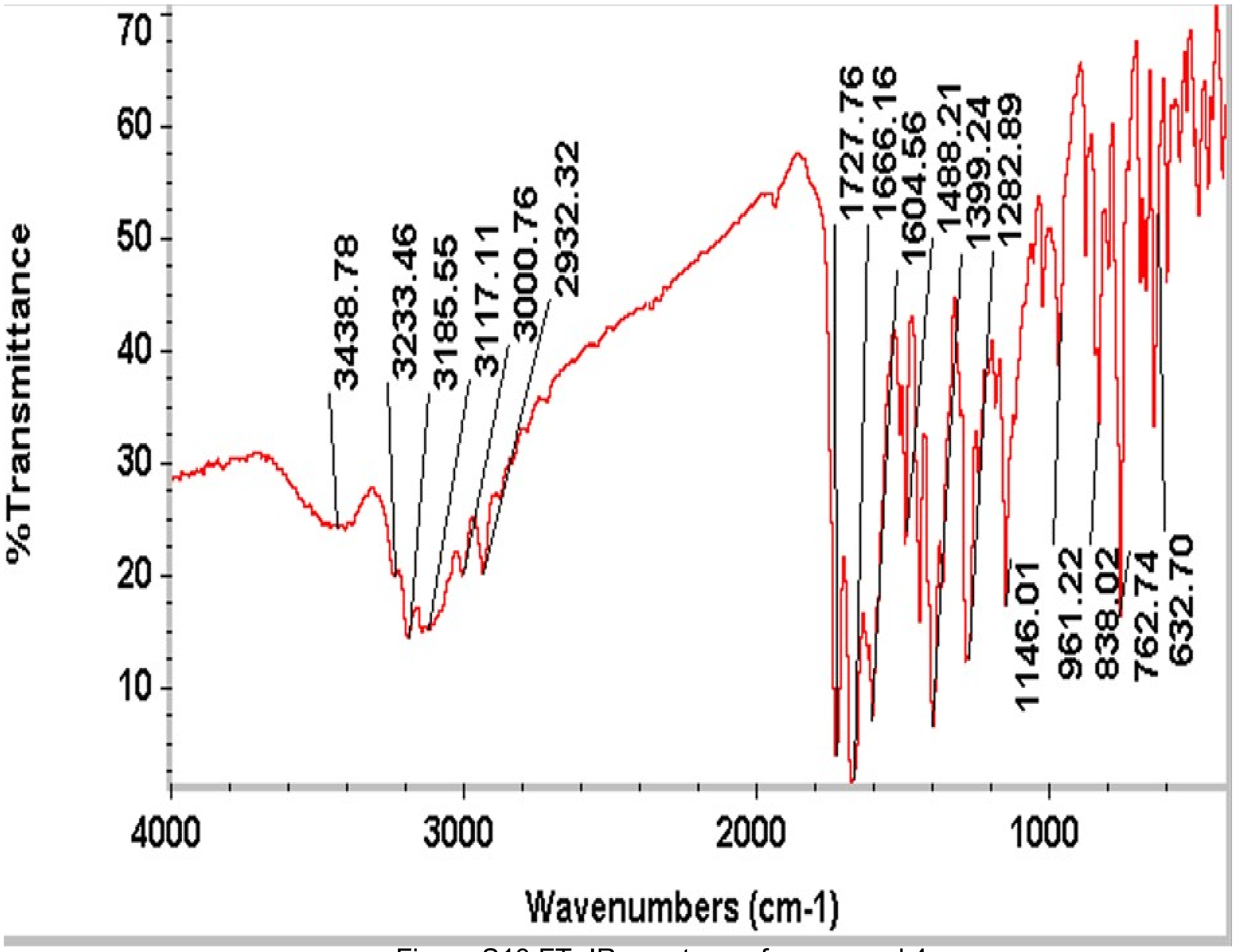

Figure S13.FT- IR spectrum of compound 4

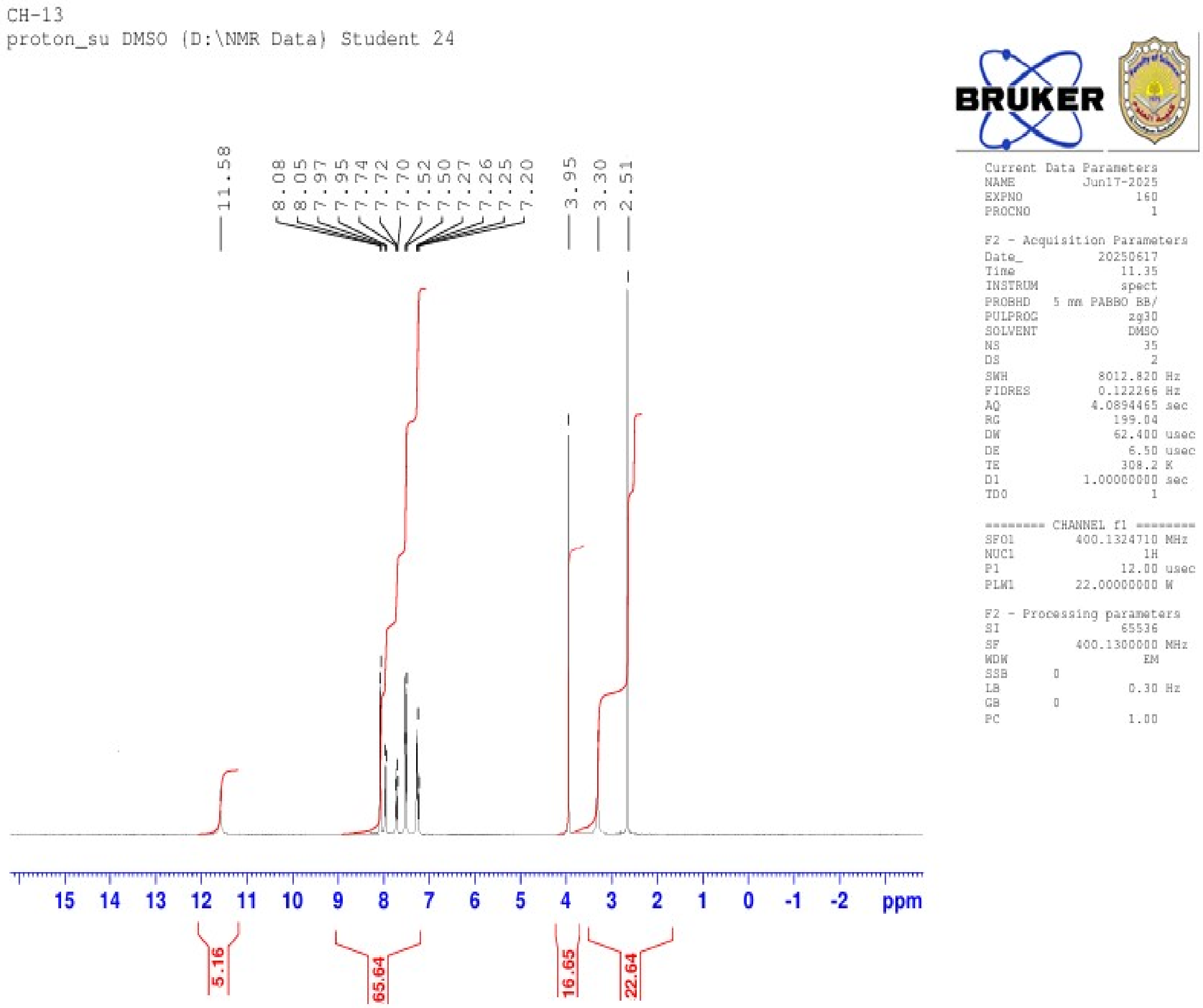

Figure S14. ^{1}HNMR spectrum of compound 4

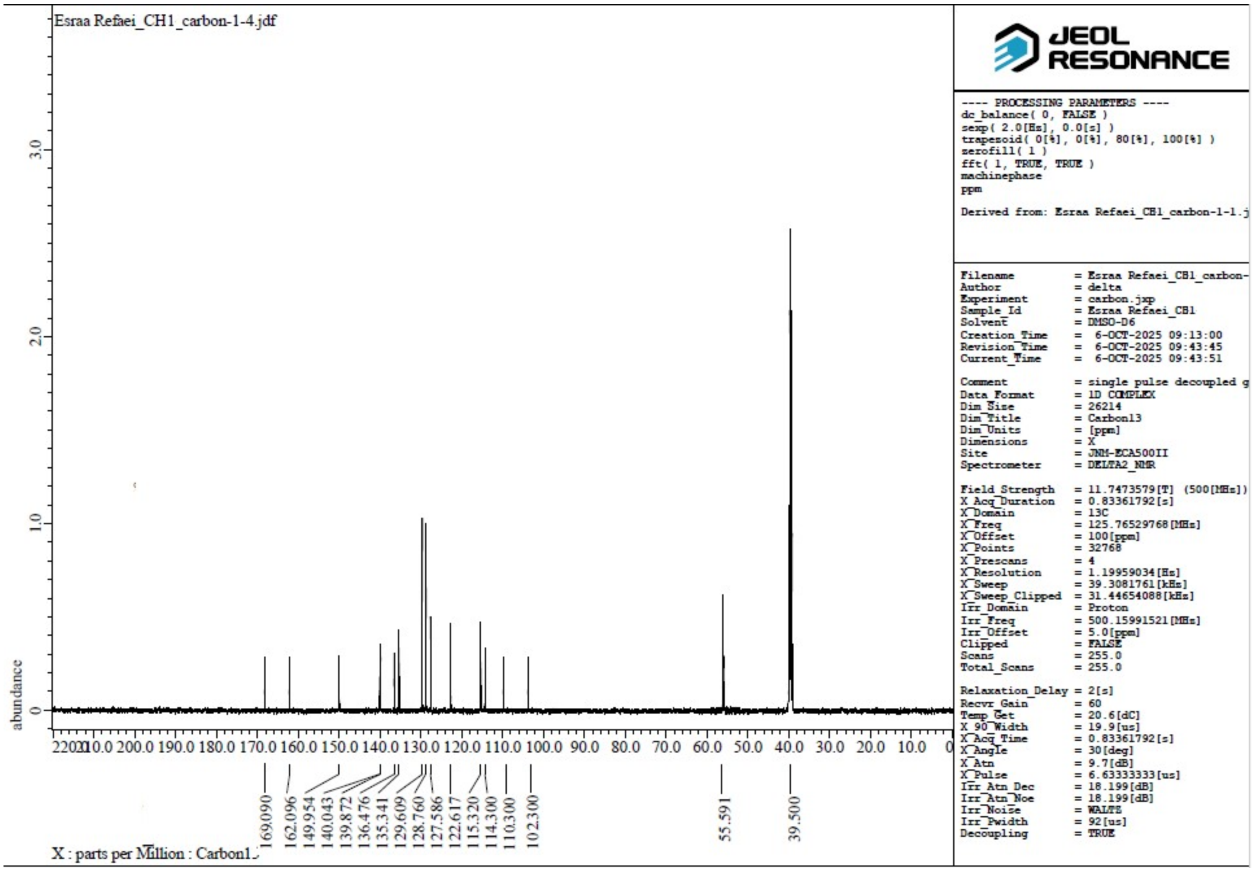

Figure S15. ^{13}CNMR spectrum of compound 4

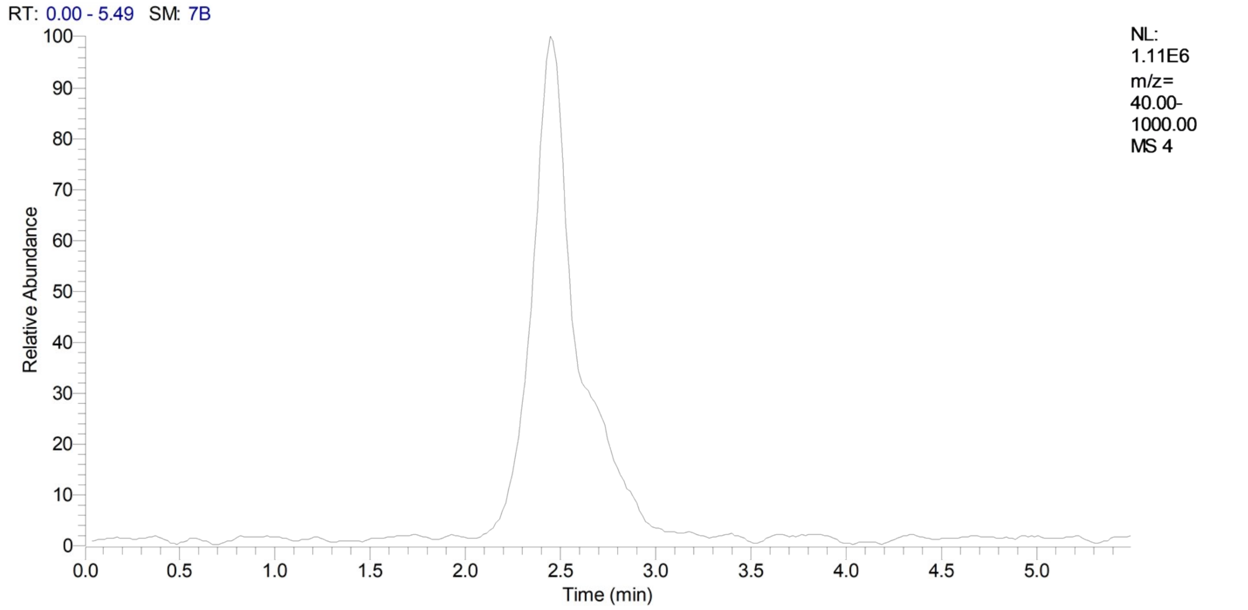
RT: 0.00 - 5.49 SM: 7B
NL:
1.11E6
m/z=
40.00-
1000.00
MS 4
Relative Abundance
Time (min)

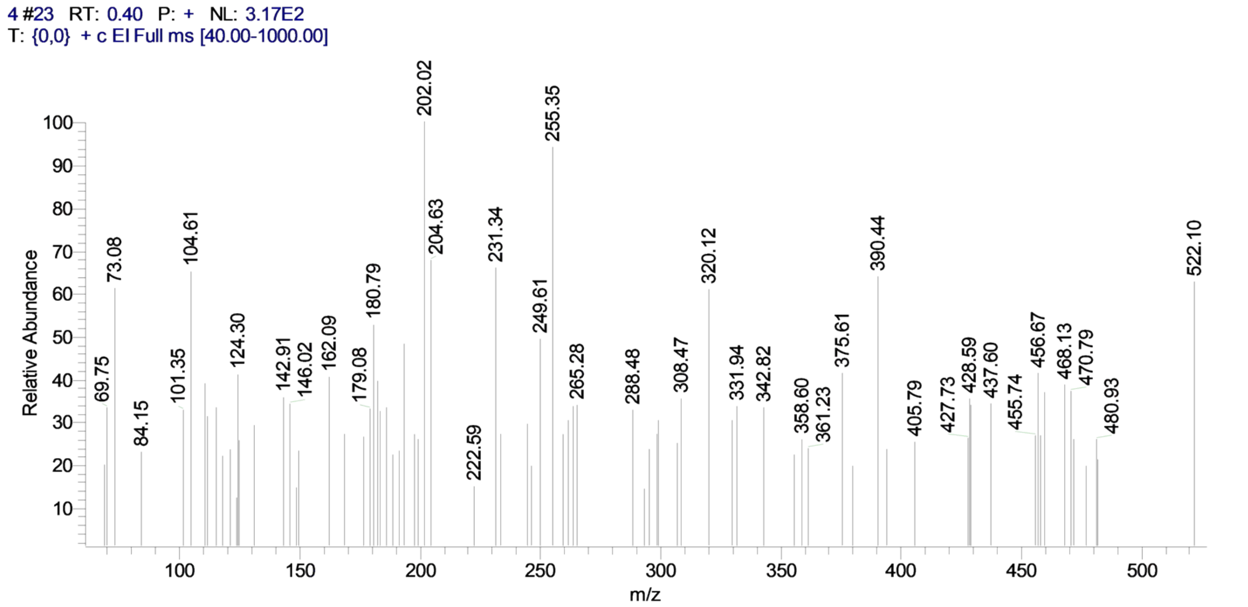
4 #23 RT: 0.40 P: + NL: 3.17E2
T: {0,0} + c EI Full ms [40.00-1000.00]
Relative Abundance
m/z
69.75
73.08
84.15
101.35
104.61
124.30
142.91
146.02
162.09
179.08
180.79
202.02
204.63
222.59
231.34
249.61
255.35
265.28
288.48
308.47
320.12
331.94
342.82
358.60
361.23
375.61
390.44
405.79
427.73
428.59
437.60
455.74
456.67
468.13
470.79
480.93
522.10

Figure S16. Mass spectrum of compound 4

Figure S17.FT- IR spectrum of compound 5

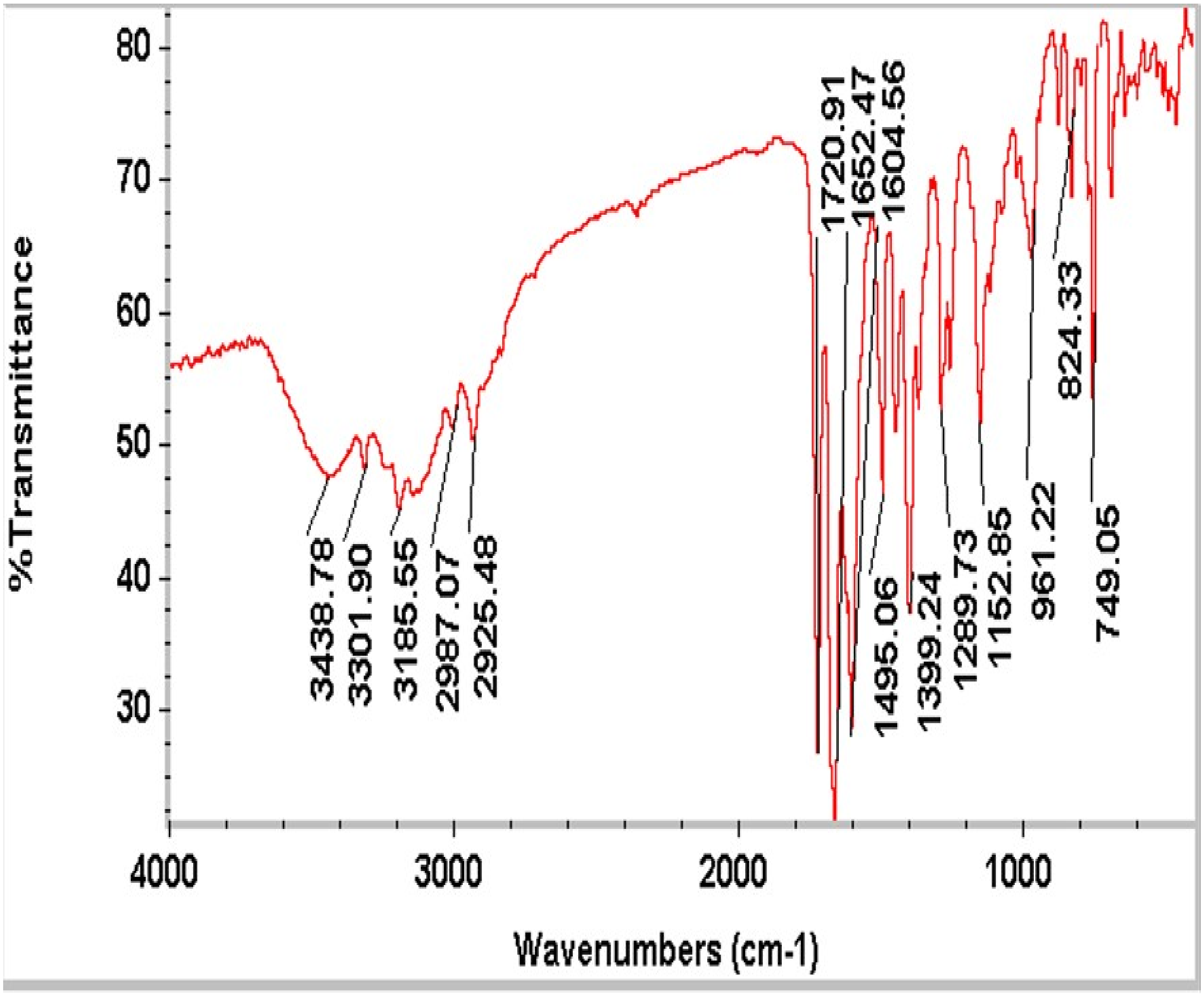

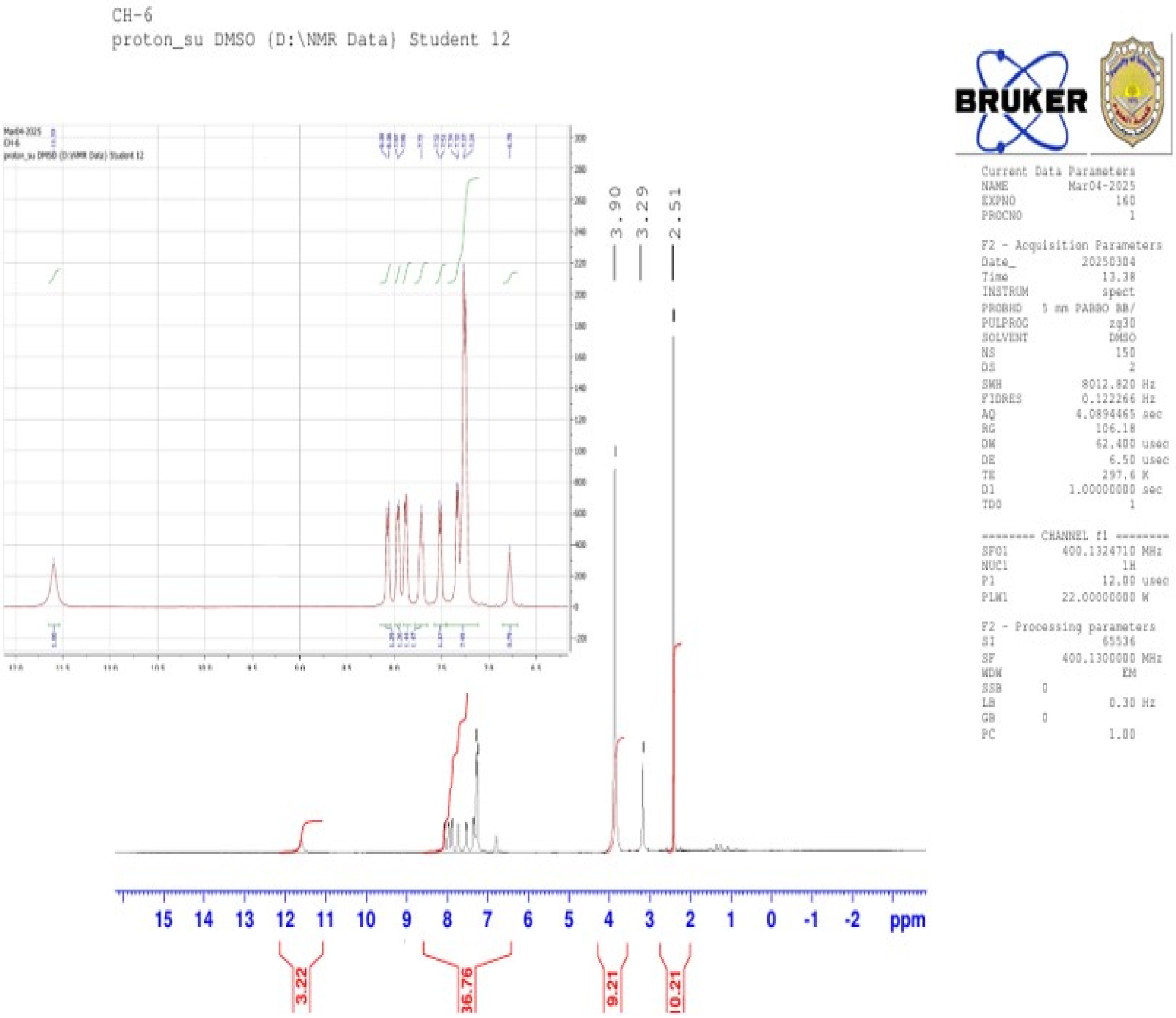

Figure S18. ^{1}HNMR spectrum of compound 5

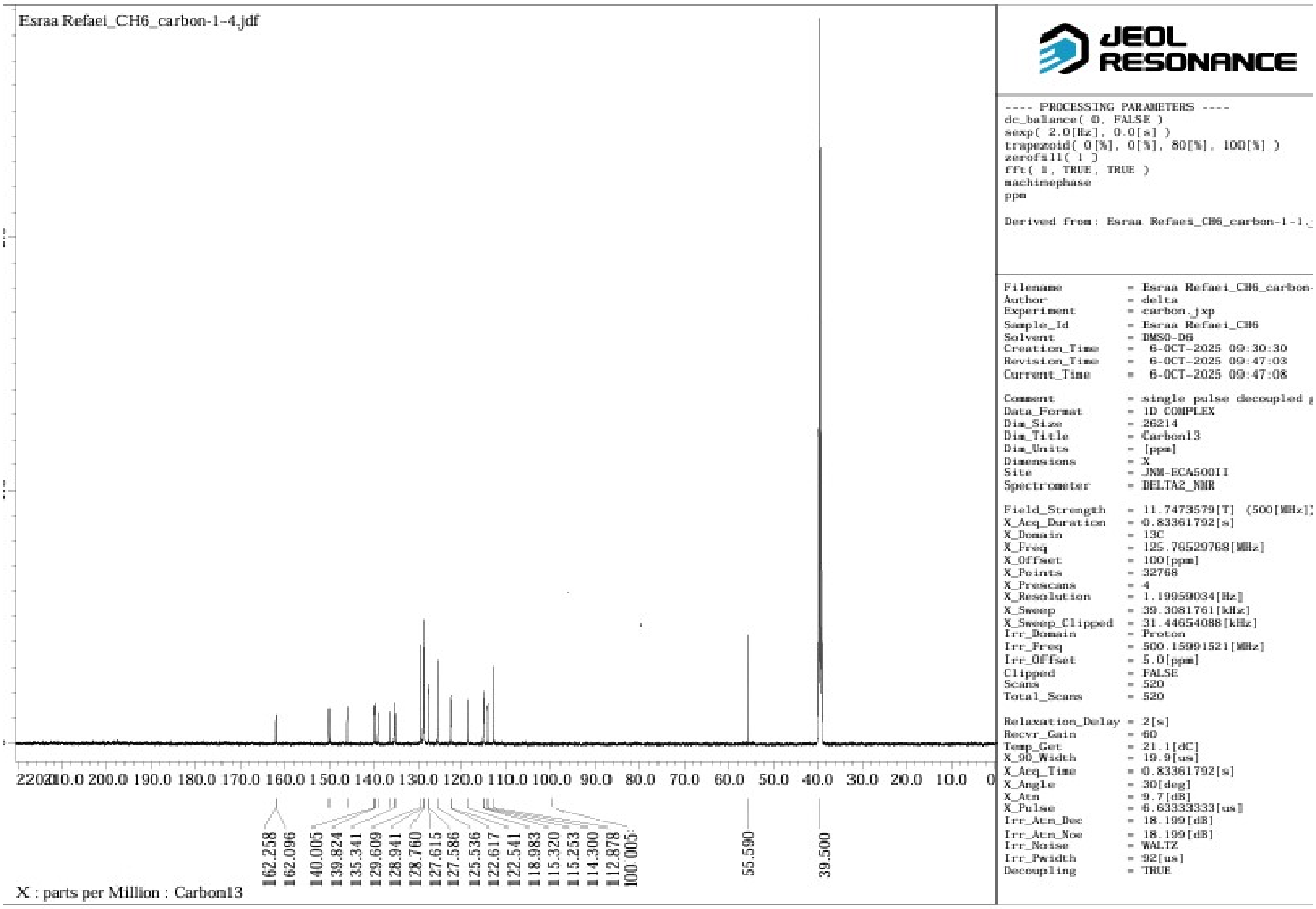

Figure S19. ^{13}CNMR spectrum of compound 5

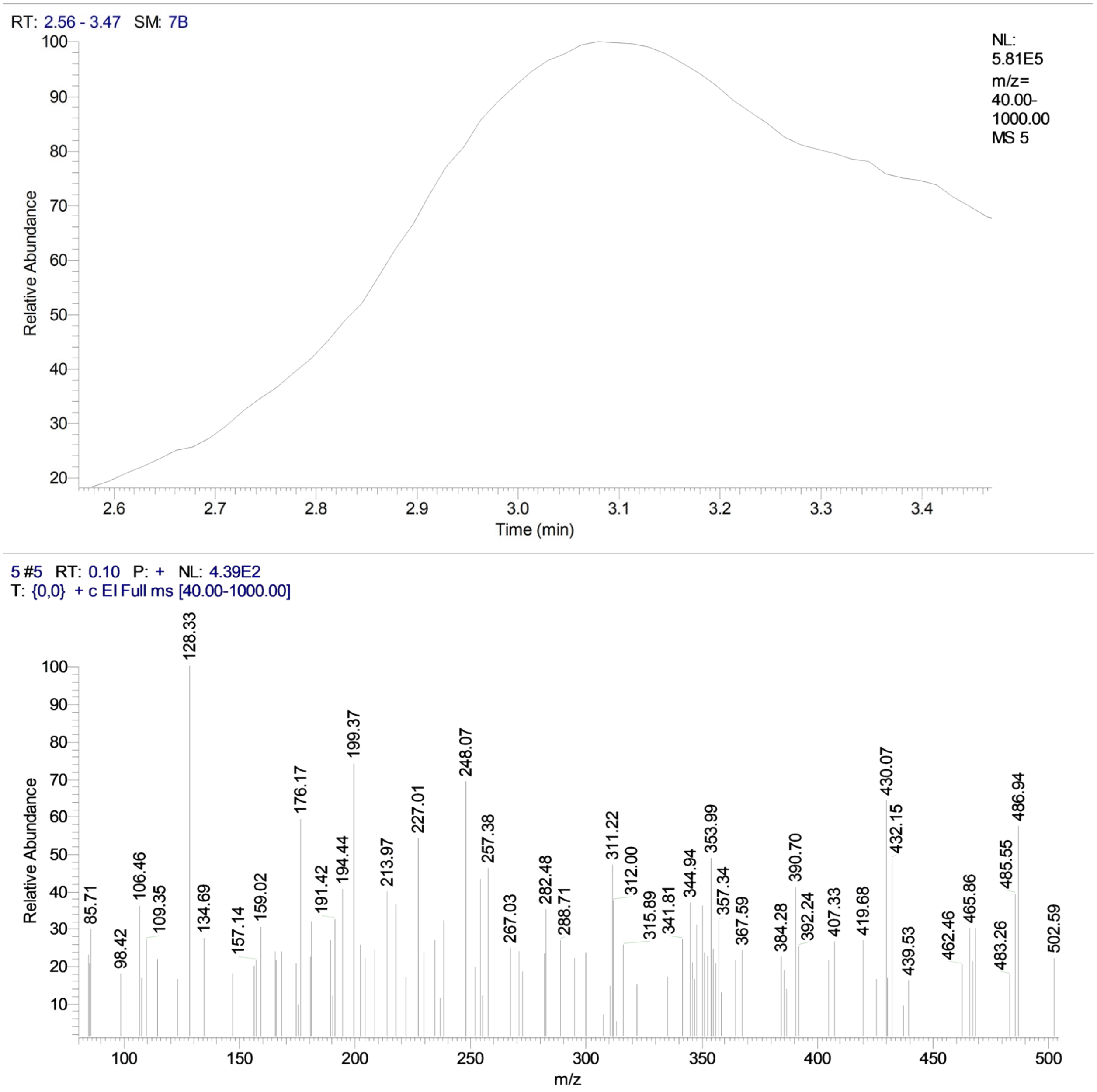

Figure S20. Mass spectrum of compound 5

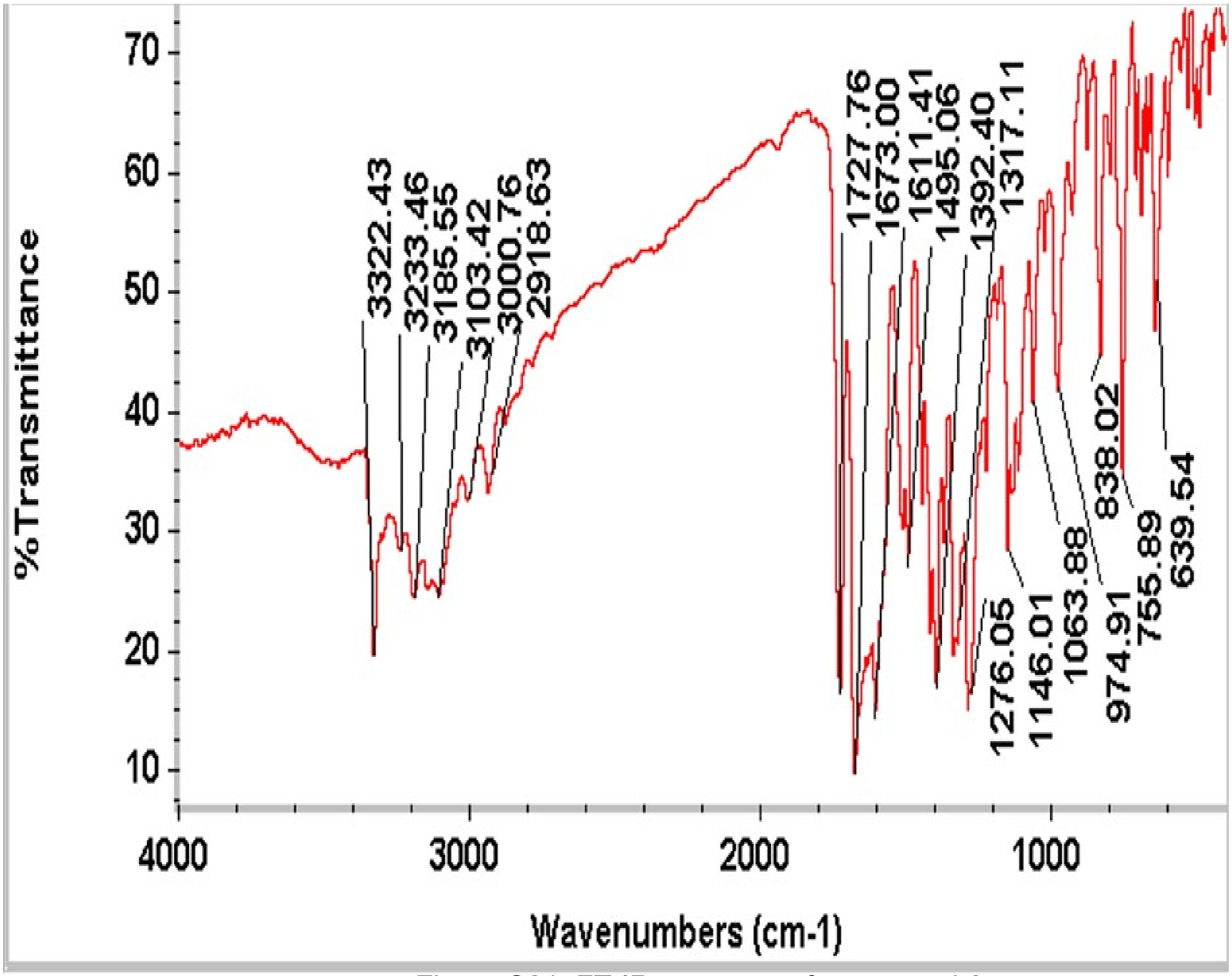

Figure S21. FT-IR spectrum of compound 6

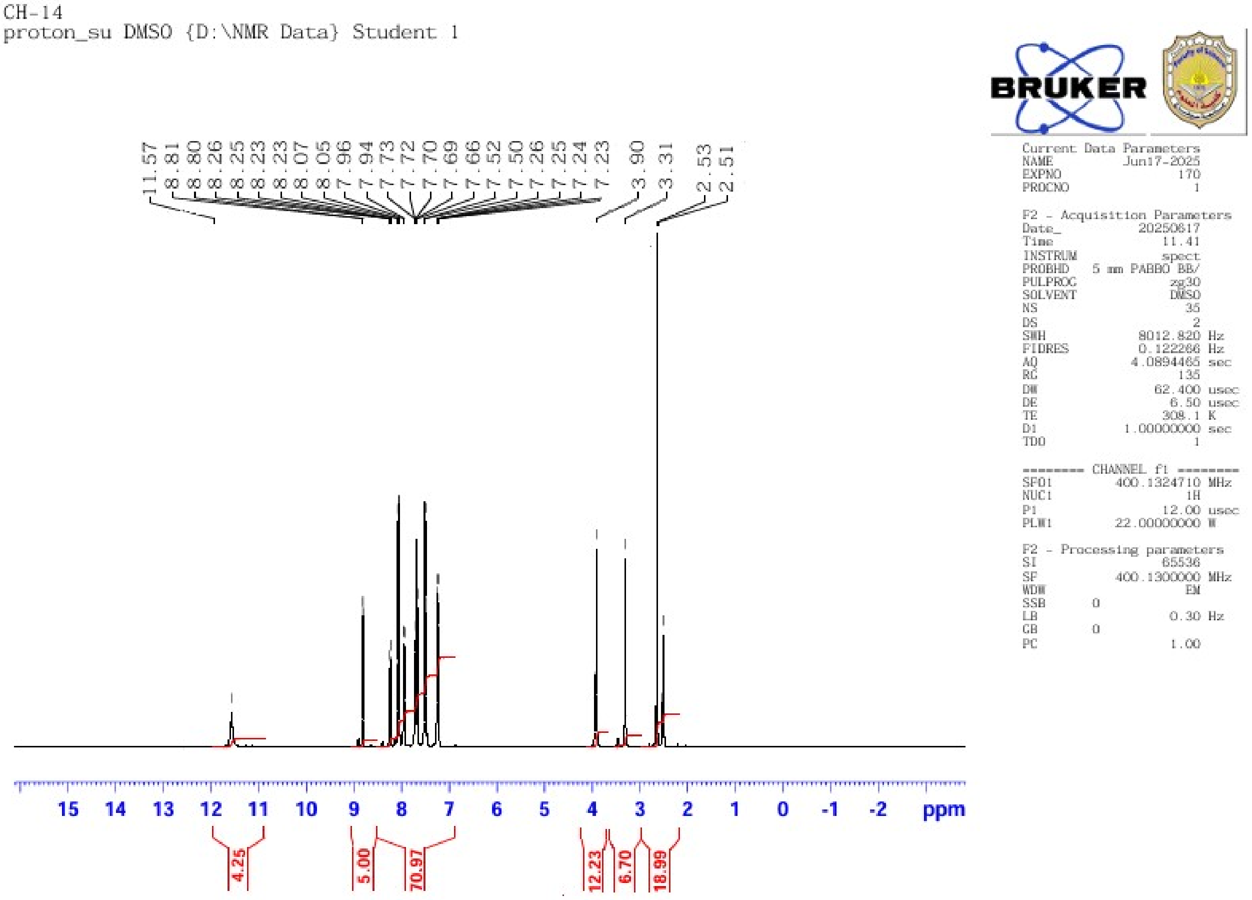

Figure S22. ^{1}HNMR spectrum of compound 6

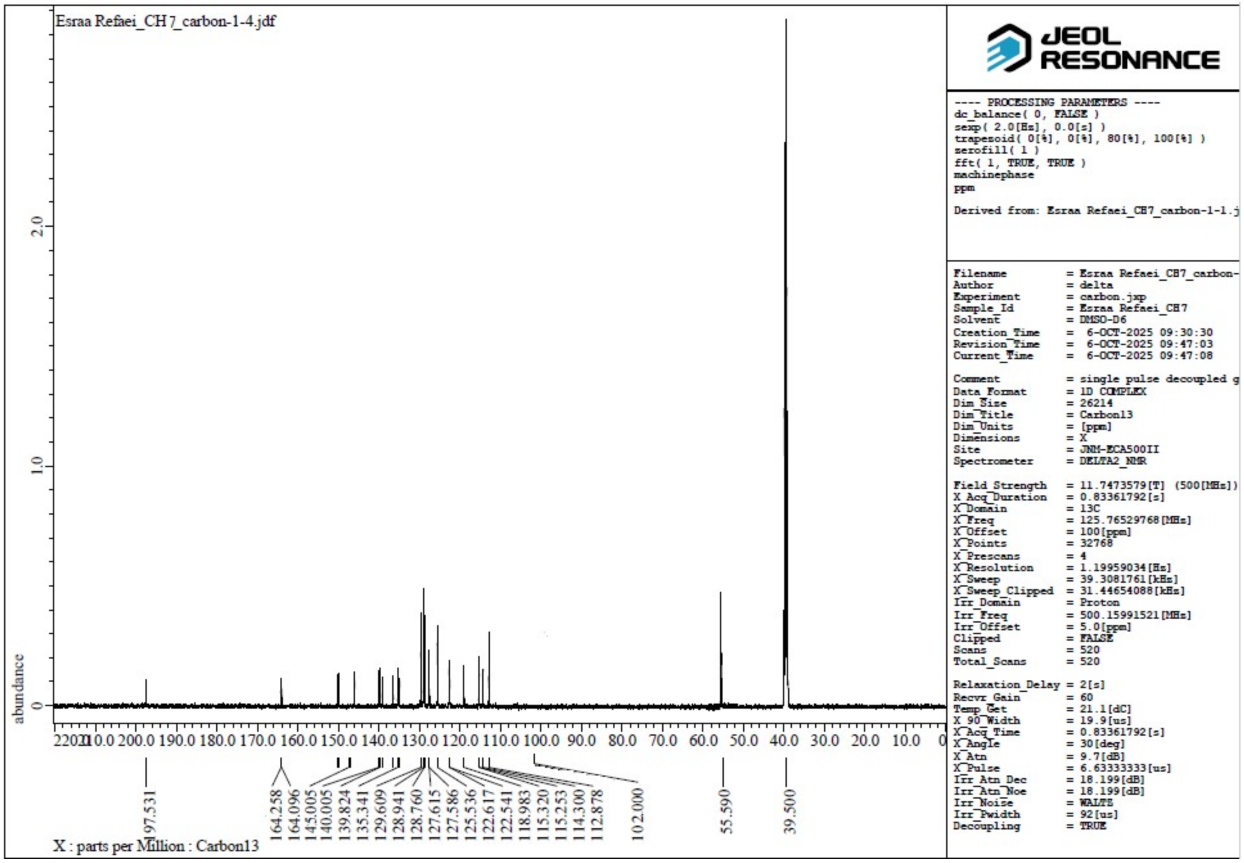

Figure S23. ^{13}CNMR spectrum of compound 6

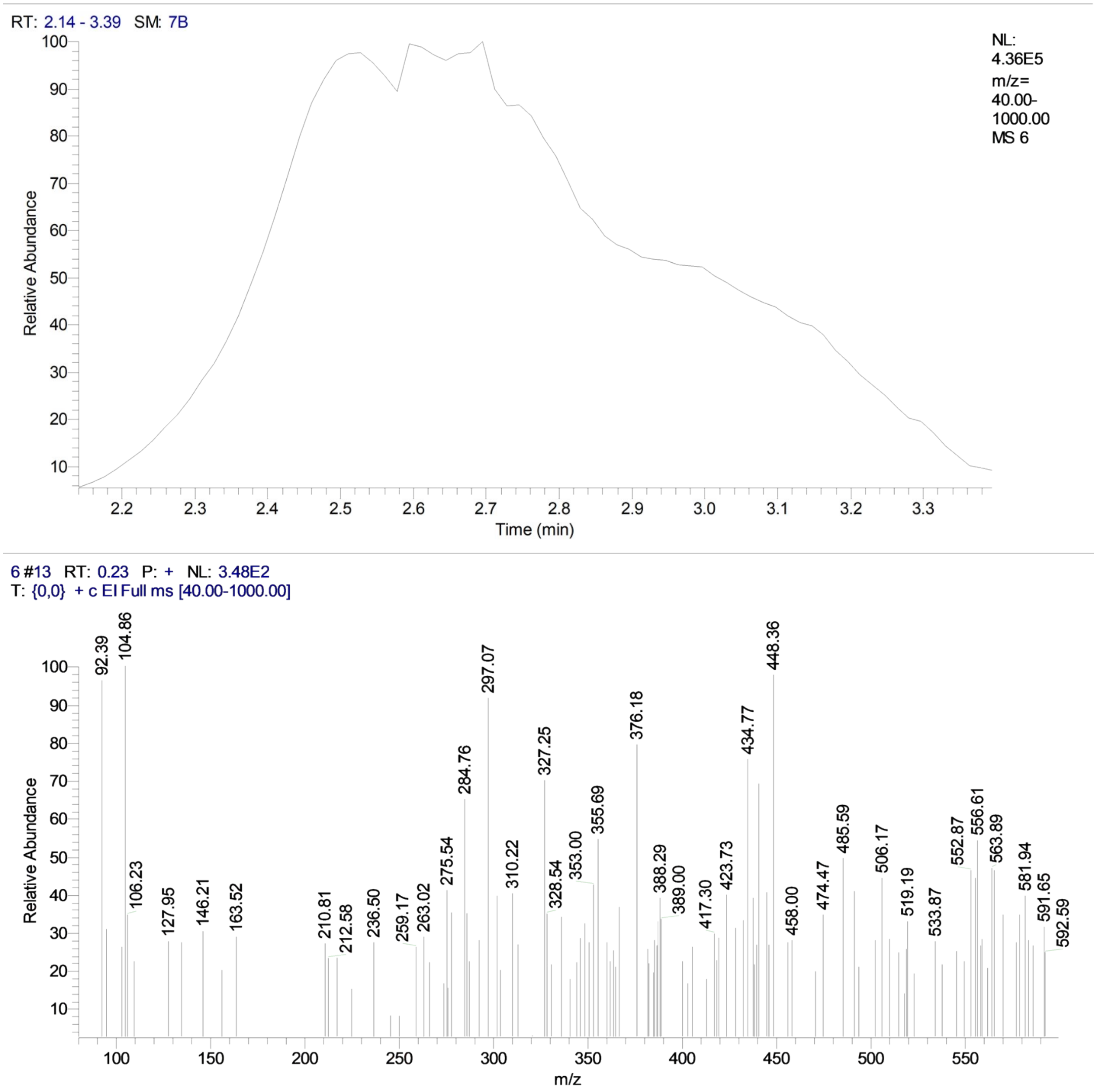

Figure S24. Mass spectrum of compound 6

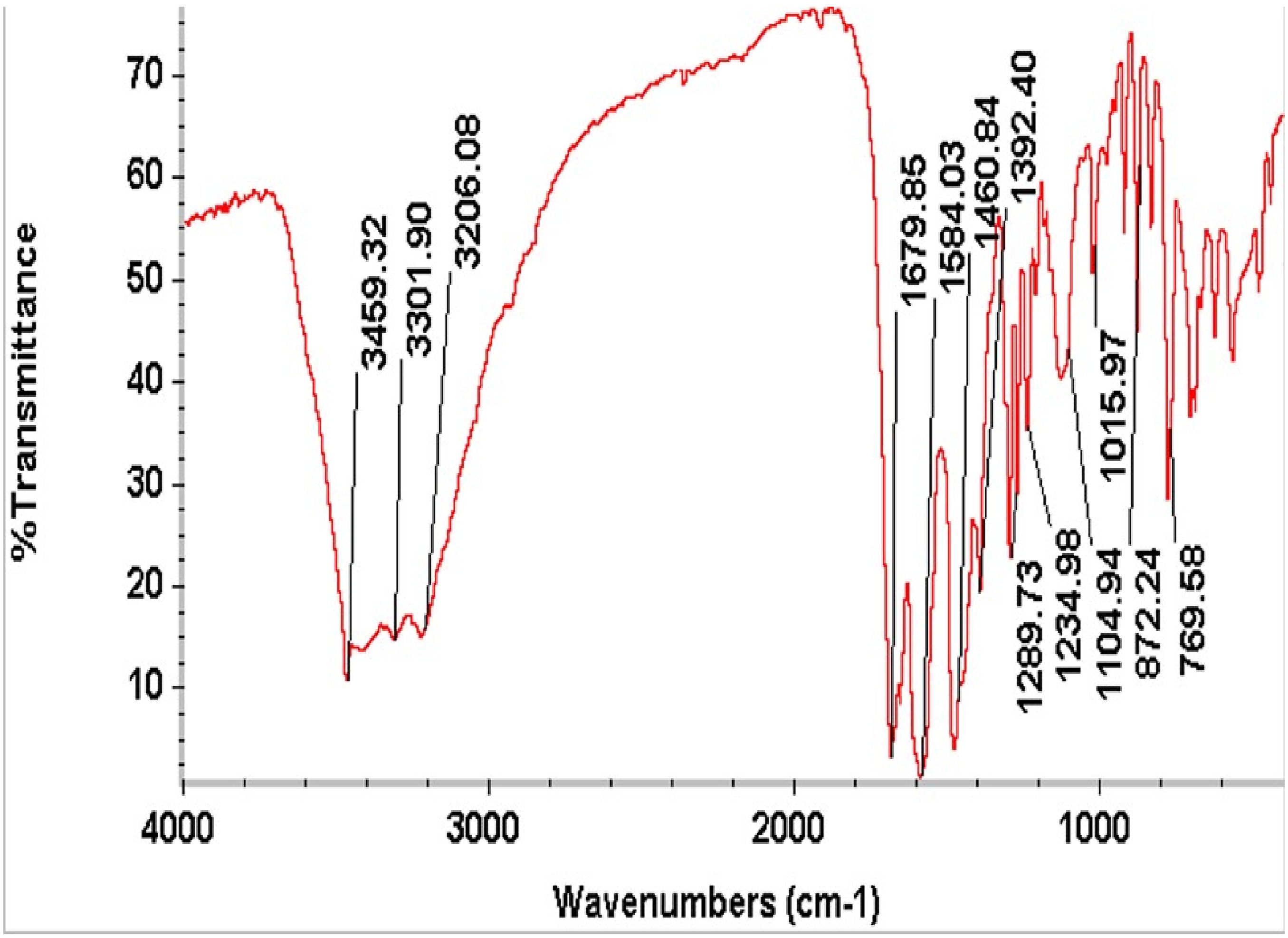

Figure S25.FT- IR spectrum of compound 7

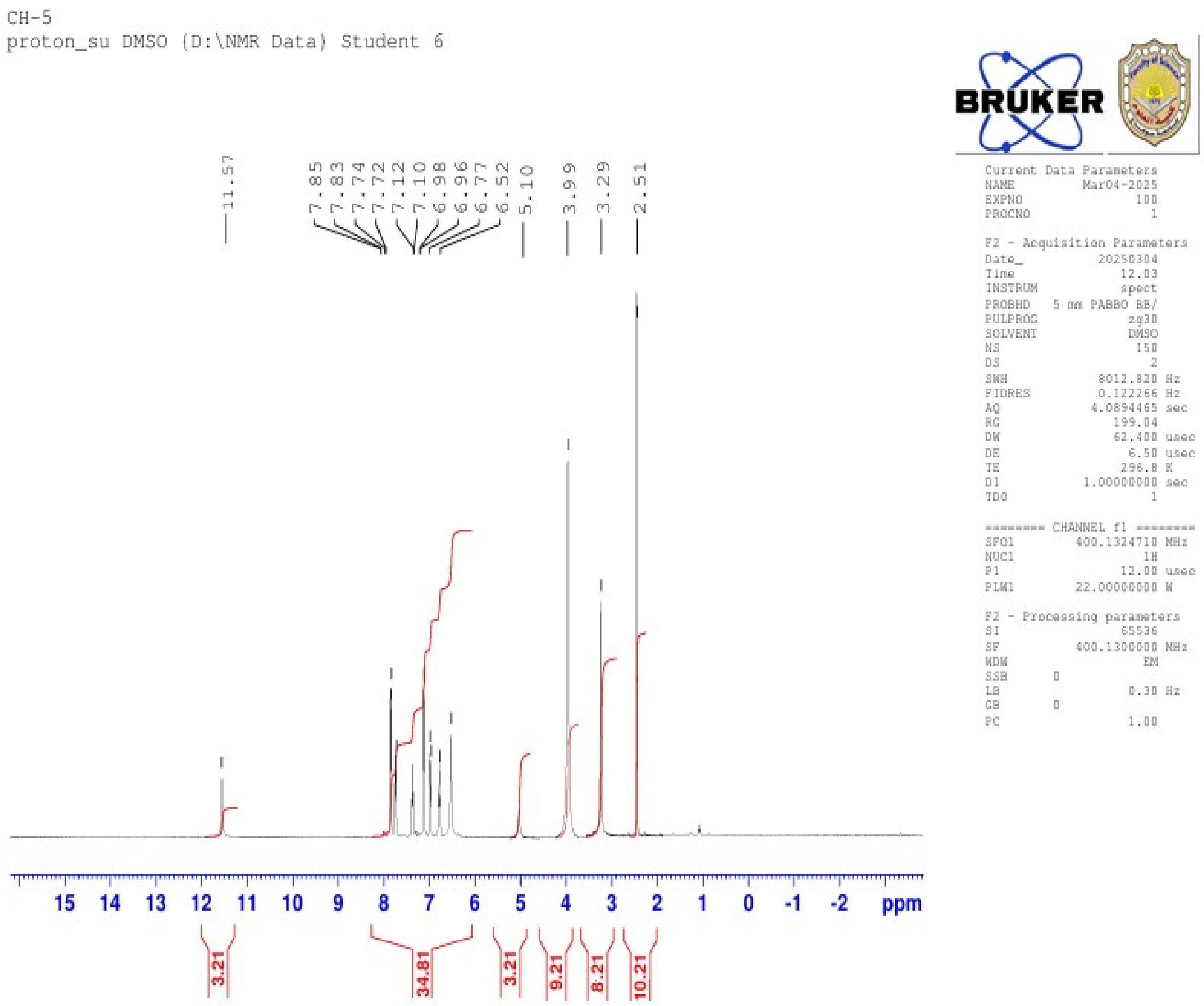

Figure S26. ^{1}HNMR spectrum of compound 7

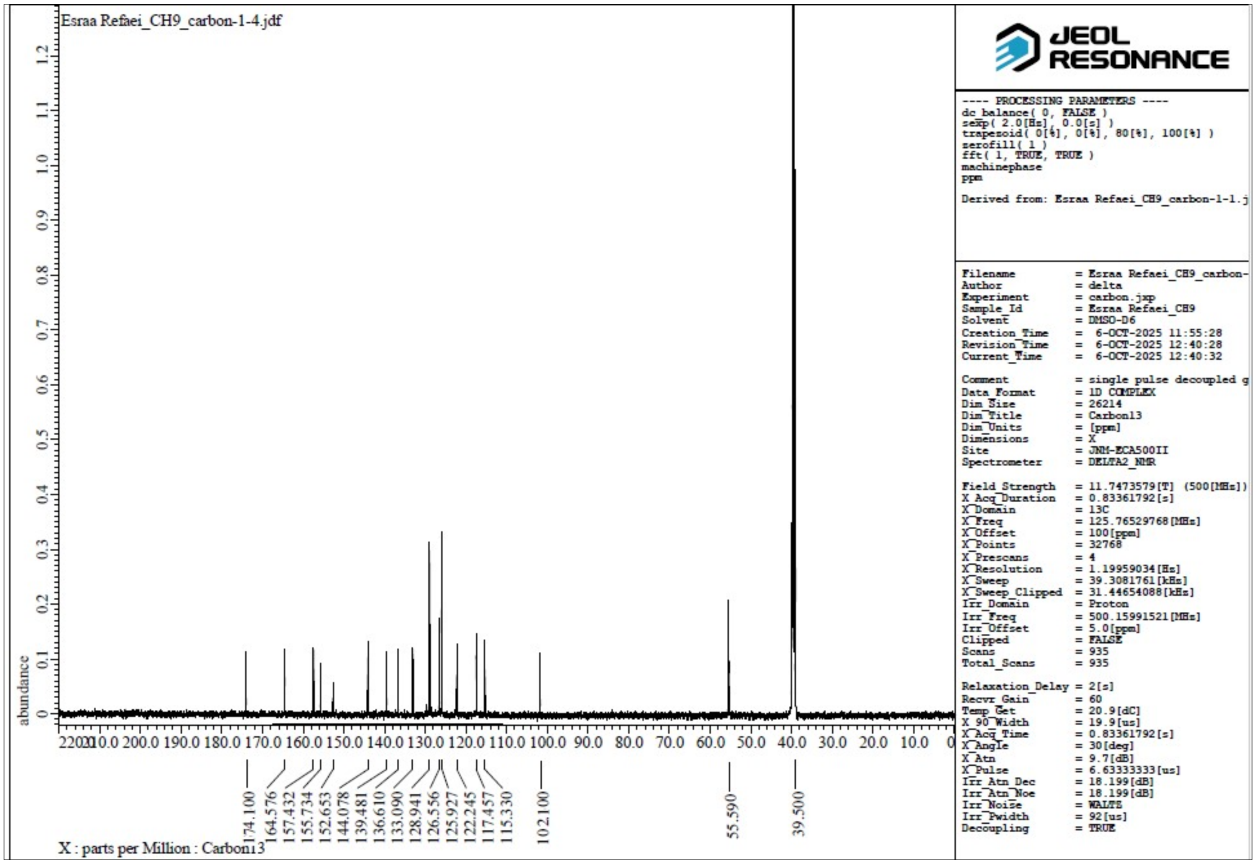

Figure S27. ^{13}CNMR spectrum of compound 7

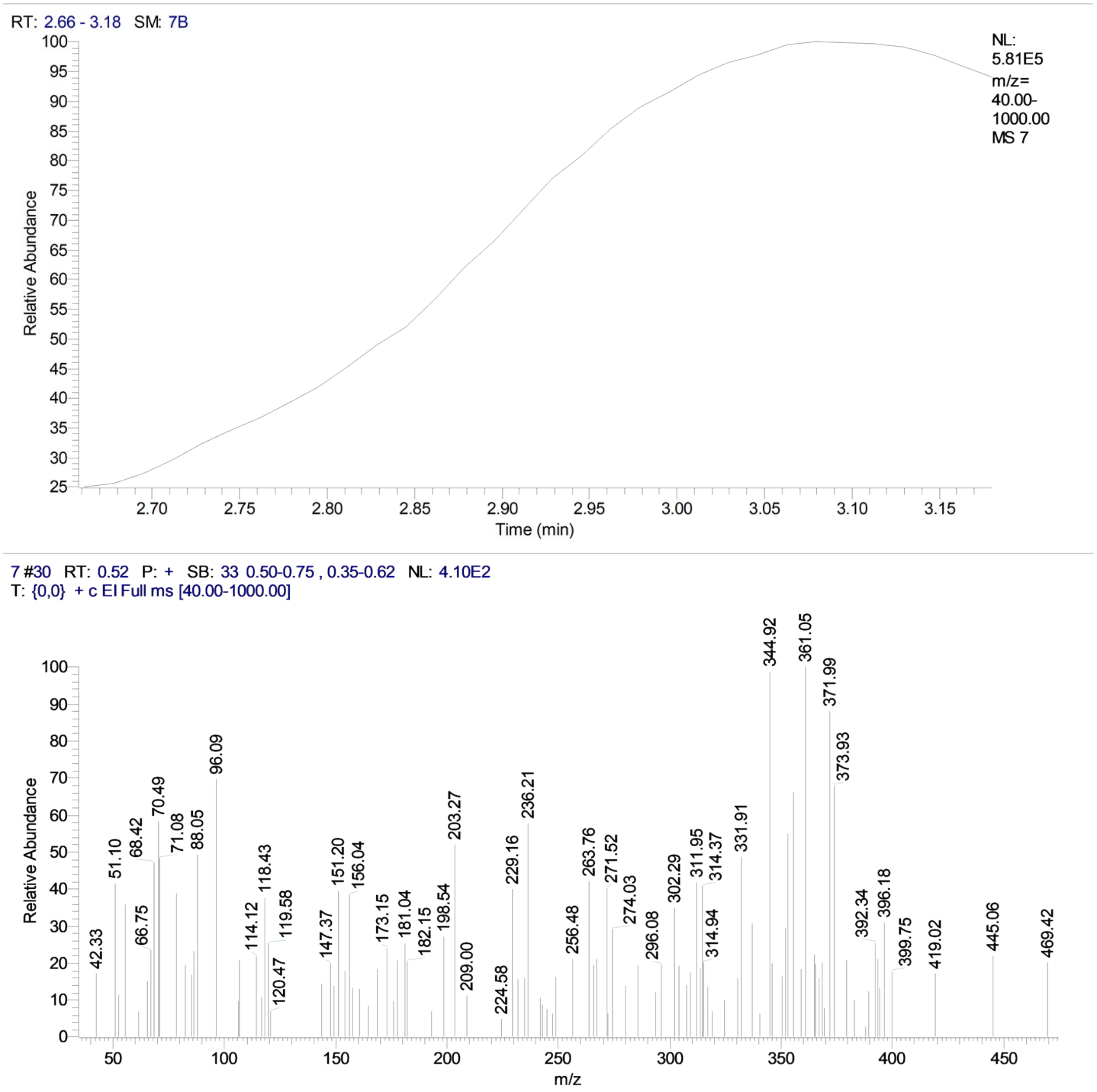

Figure S28. Mass spectrum of compound 7

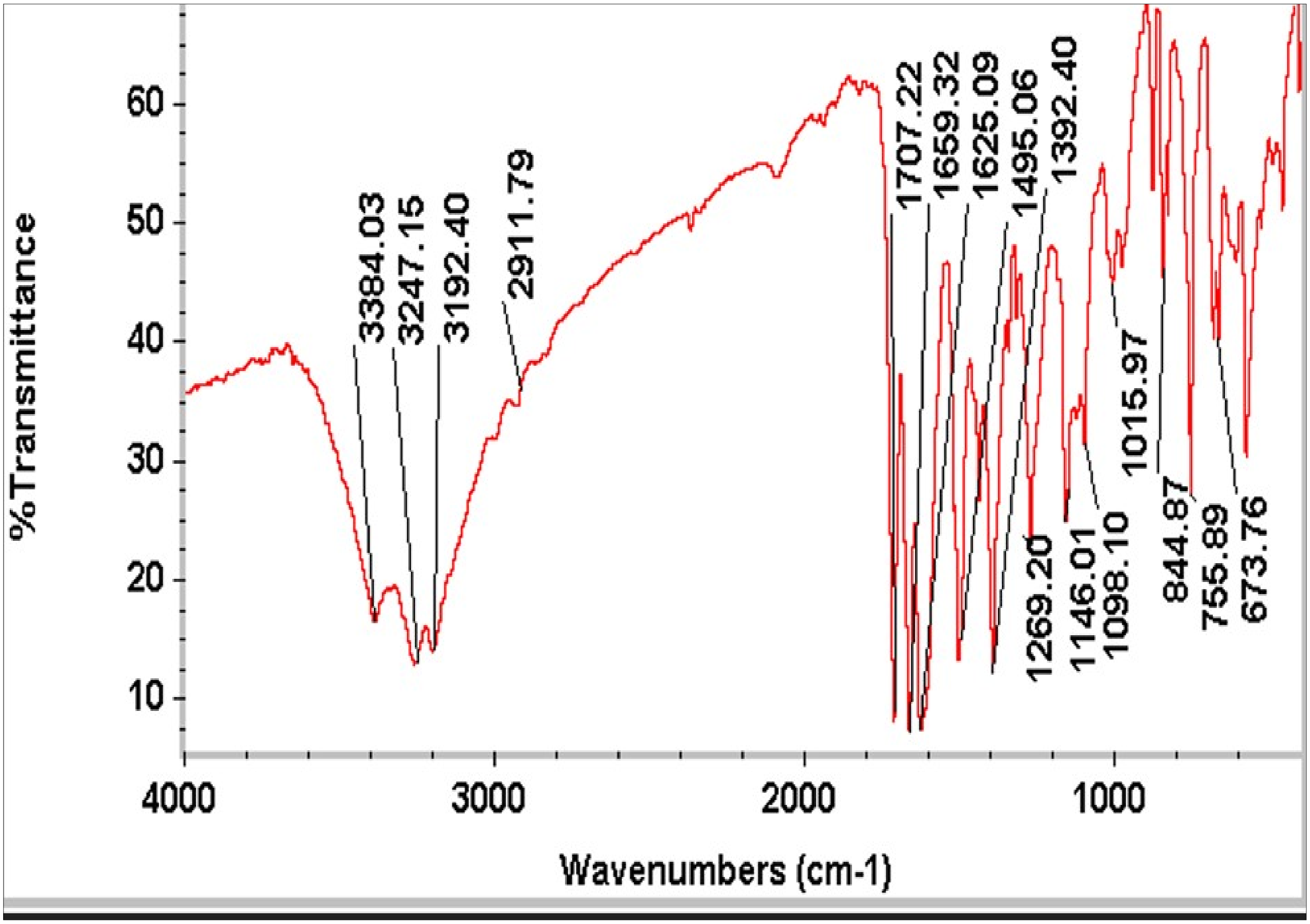

Figure S29.FT- IR spectrum of compound 8

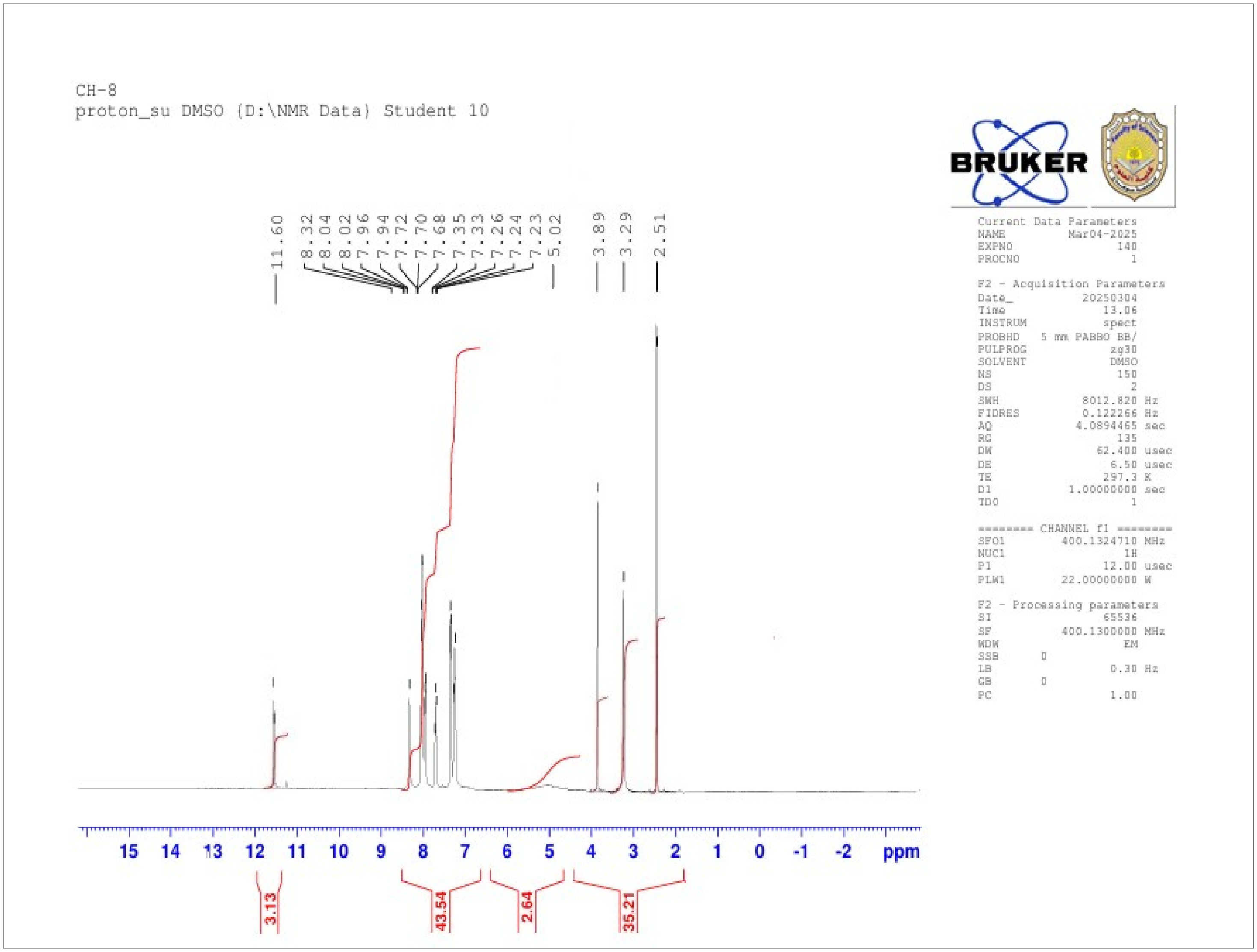

Figure S30. ^{1}HNMR spectrum of compound 8

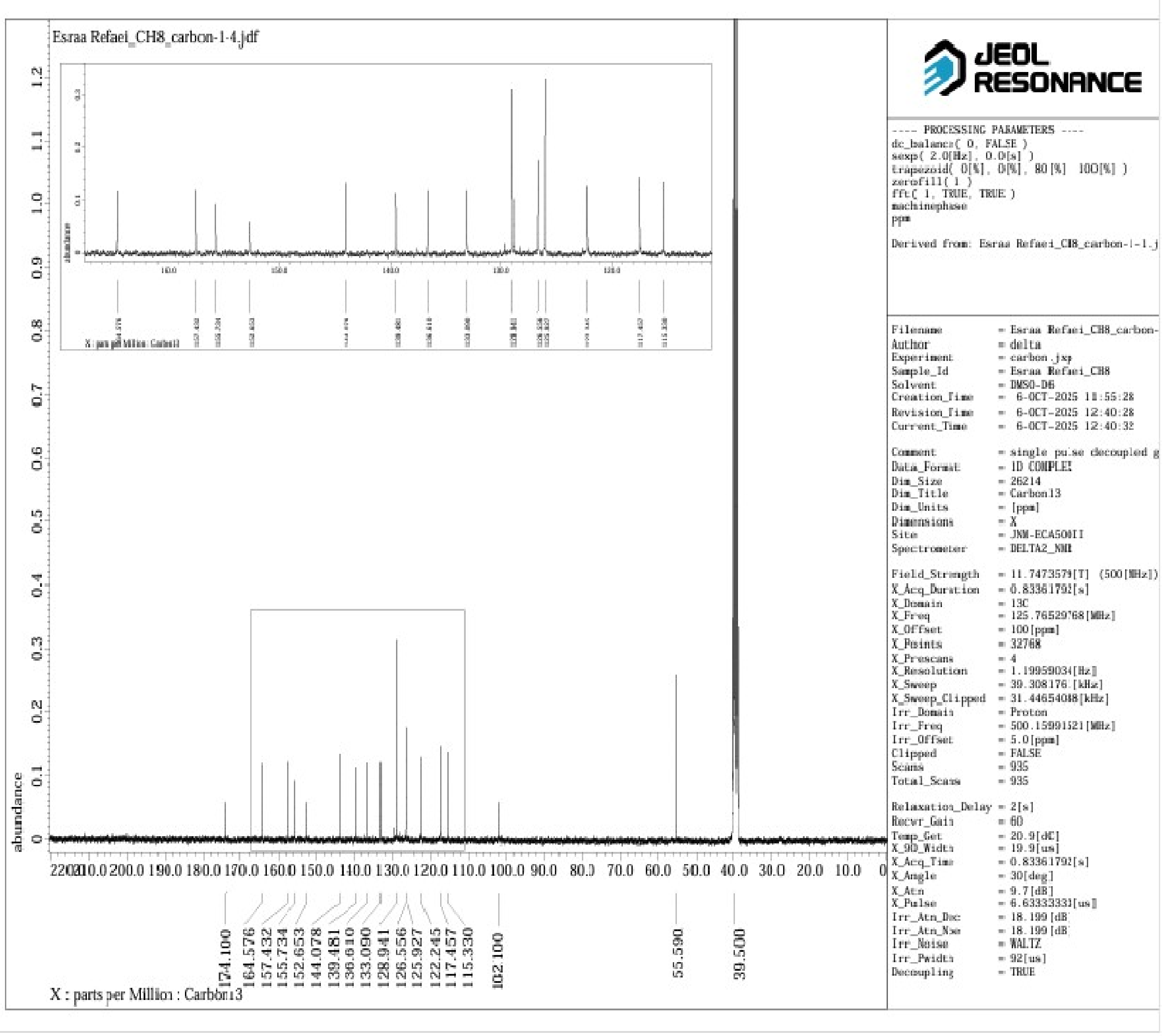

Figure S31. ^{13}CNMR spectrum of compound 8

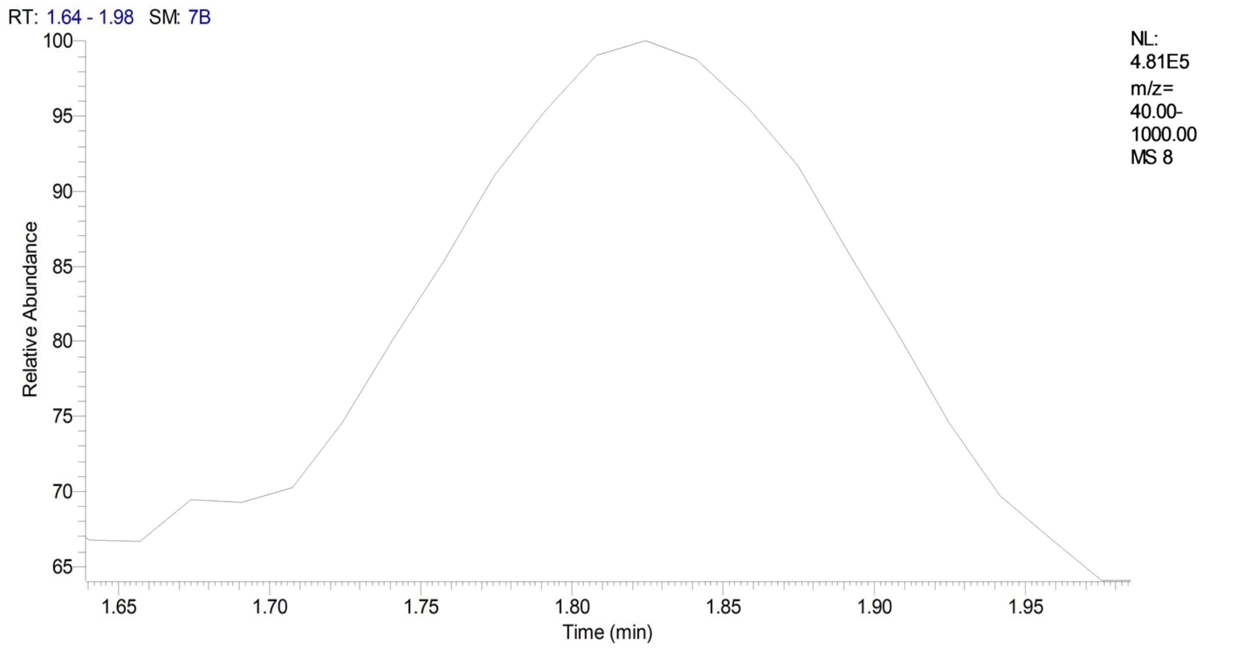

RT: 1.64 - 1.98 SM: 7B
NL: 4.81E5 m/z= 40.00-1000.00 MS 8
Relative Abundance
Time (min)

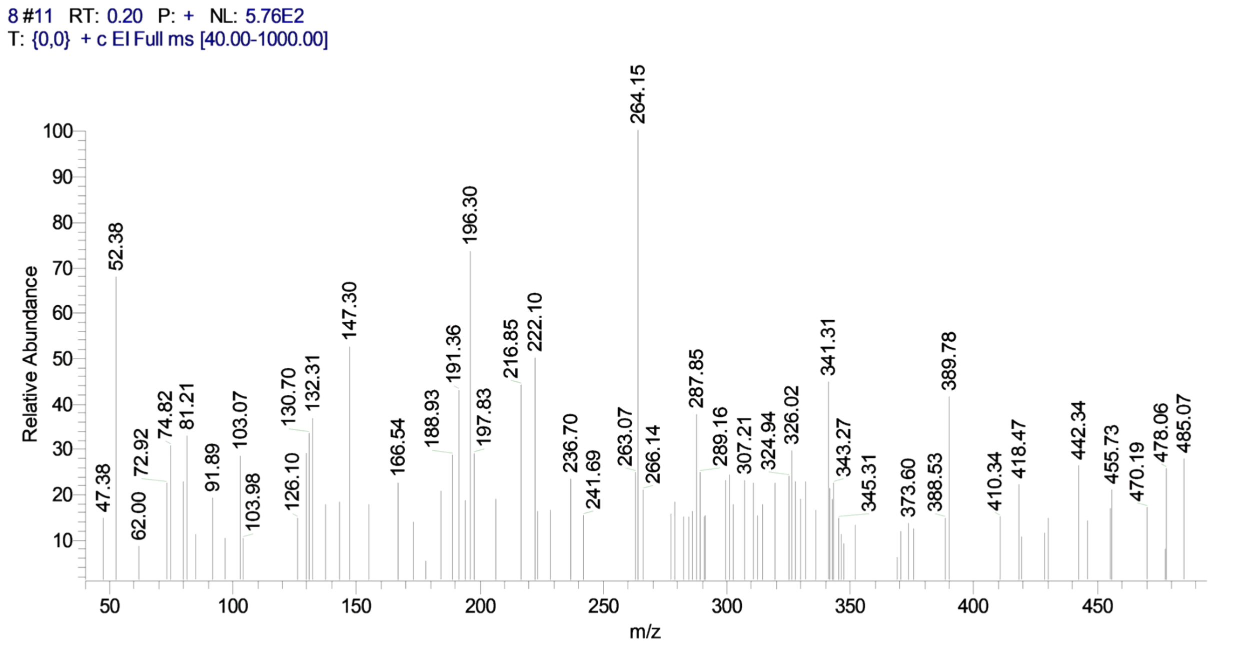

8 #11 RT: 0.20 P: + NL: 5.76E2
T: {0,0} + c EI Full ms [40.00-1000.00]
264.15
196.30
52.38
147.30
222.10
216.85
191.36
341.31
389.78
287.85
Relative Abundance
m/z

Figure

S32. Mass spectrum of compound 8

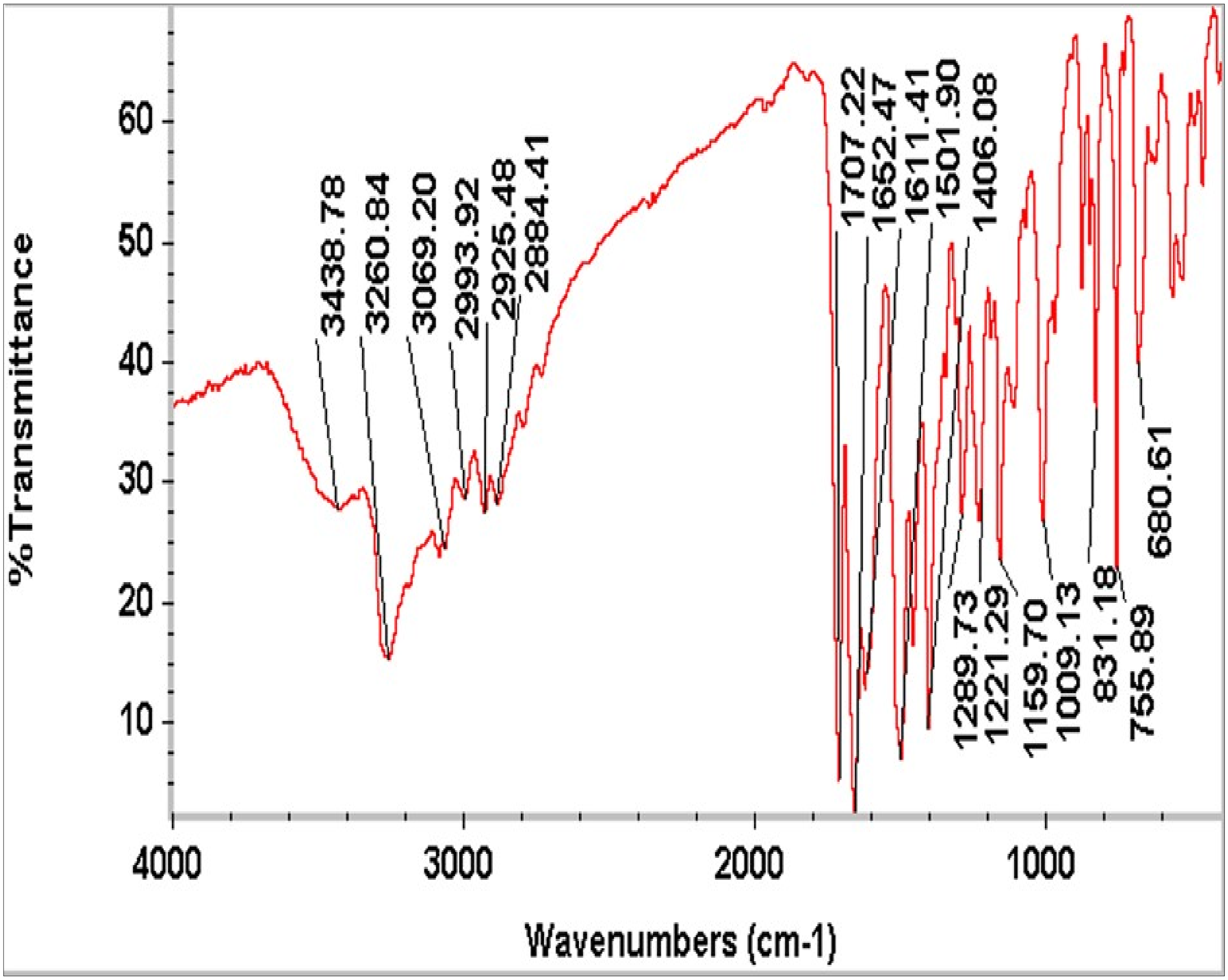

Figure S33. FT-IR spectrum of compound 9

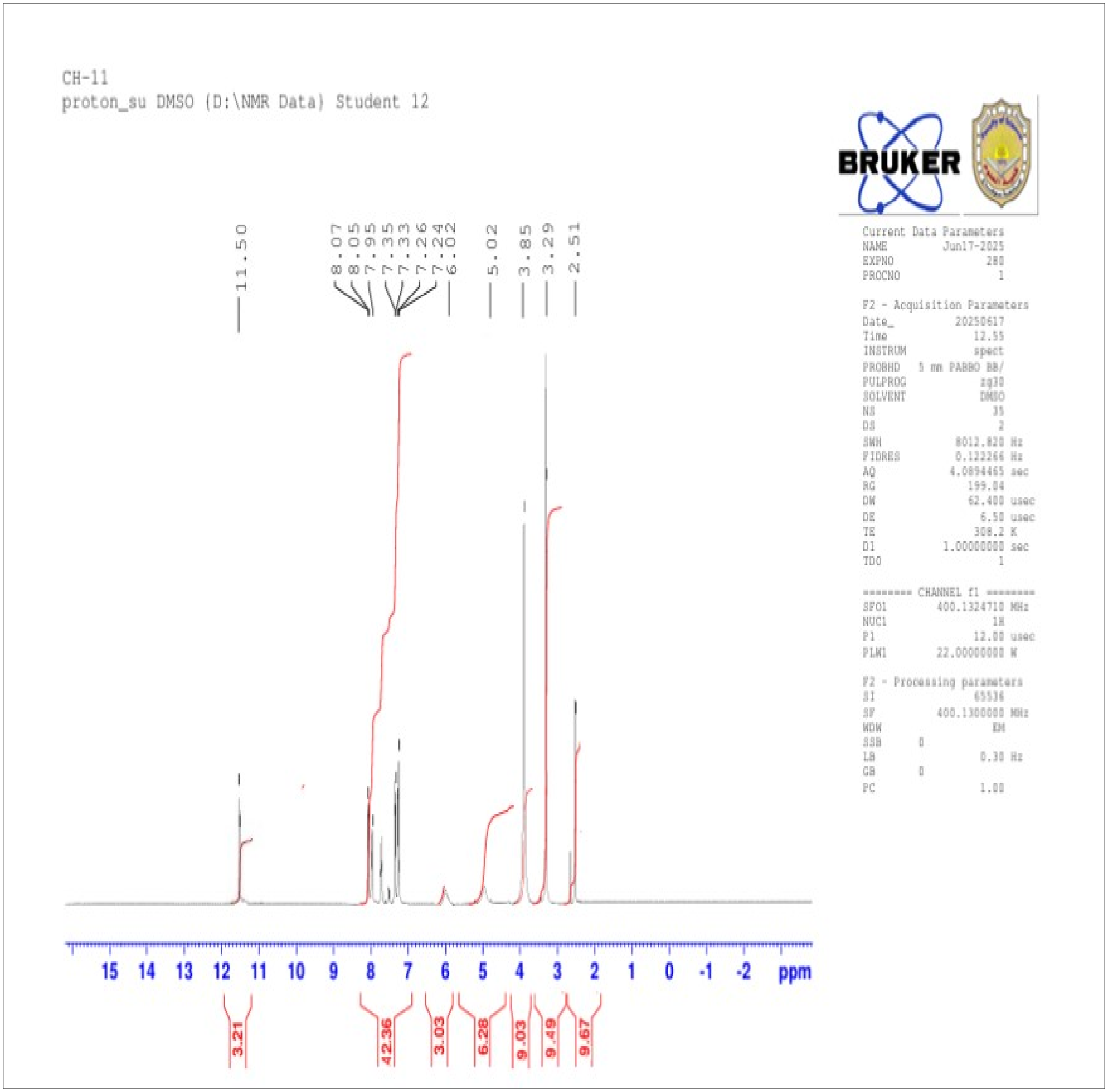

Figure S34. ^{1}HNMR spectrum of compound 9

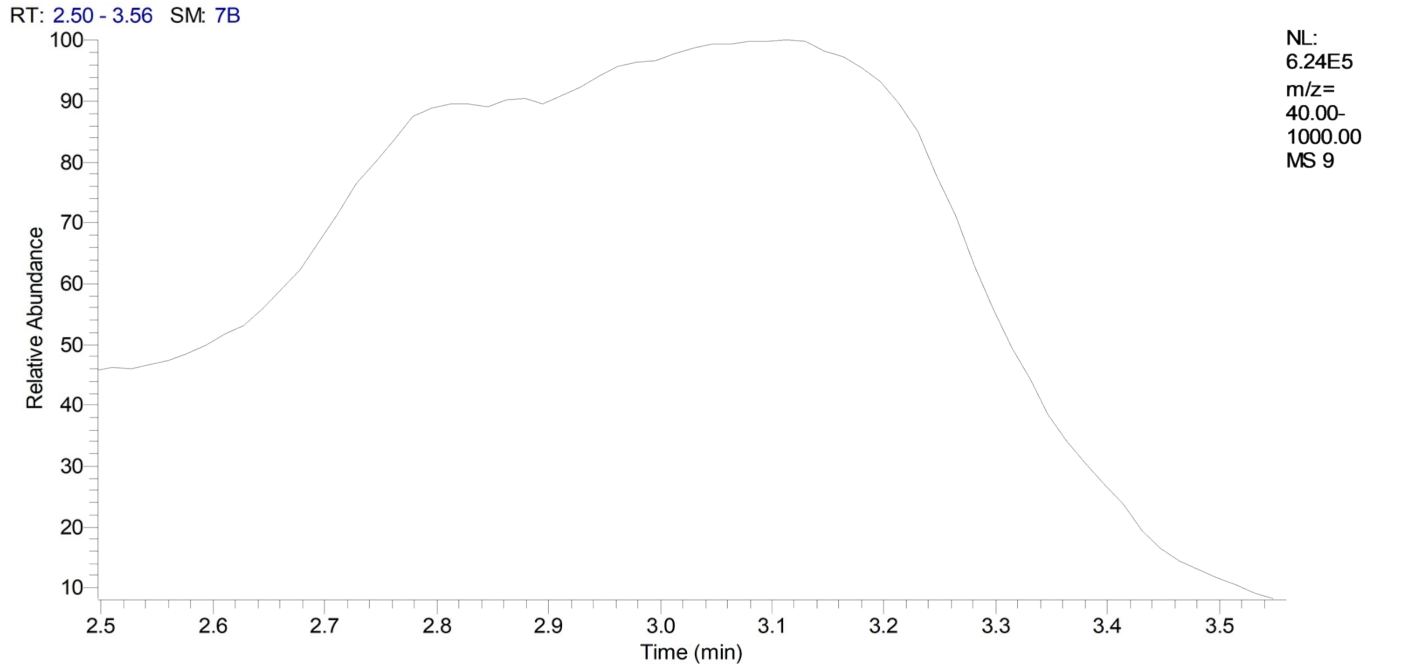
RT: 2.50 - 3.56 SM: 7B
NL: 6.24E5 m/z= 40.00-1000.00 MS 9
Relative Abundance
Time (min)

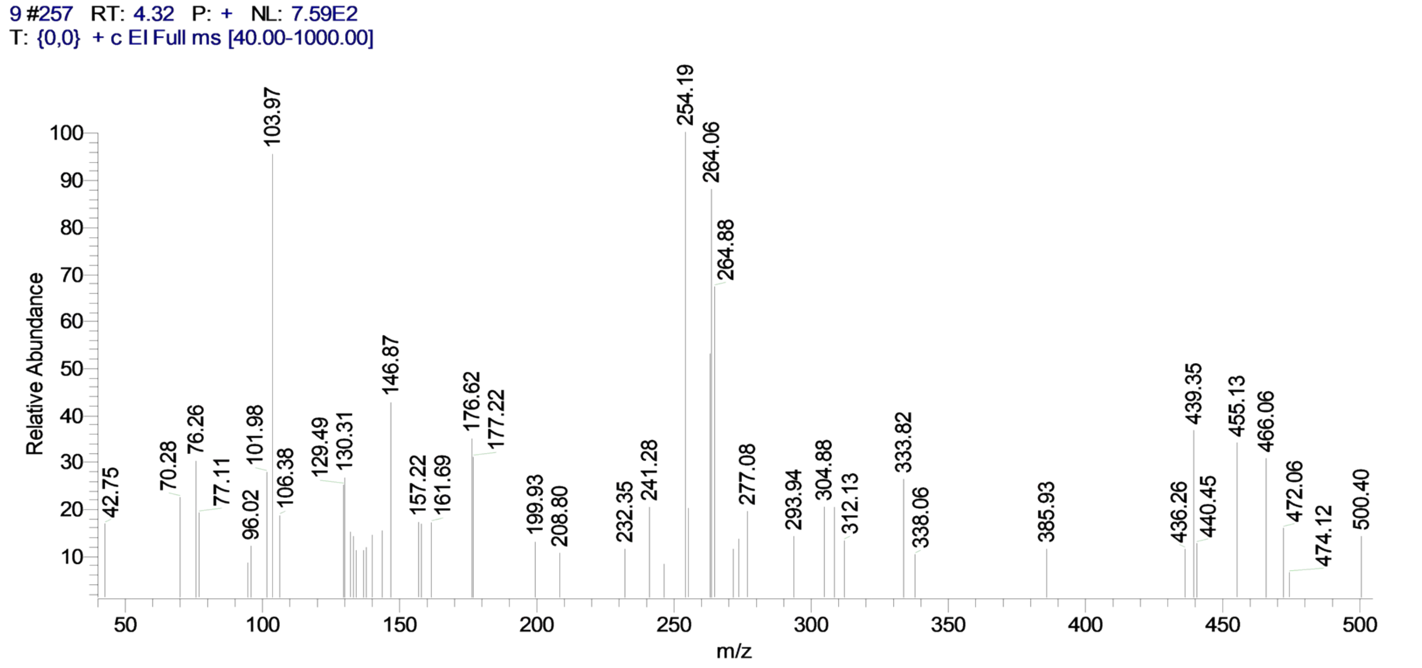
9 #257 RT: 4.32 P: + NL: 7.59E2
T: {0,0} + c EI Full ms [40.00-1000.00]
Relative Abundance
m/z
103.97
254.19
264.06
264.88
146.87
176.62
177.22
439.35
455.13
466.06
333.82

Figure S35. Mass spectrum of compound 9

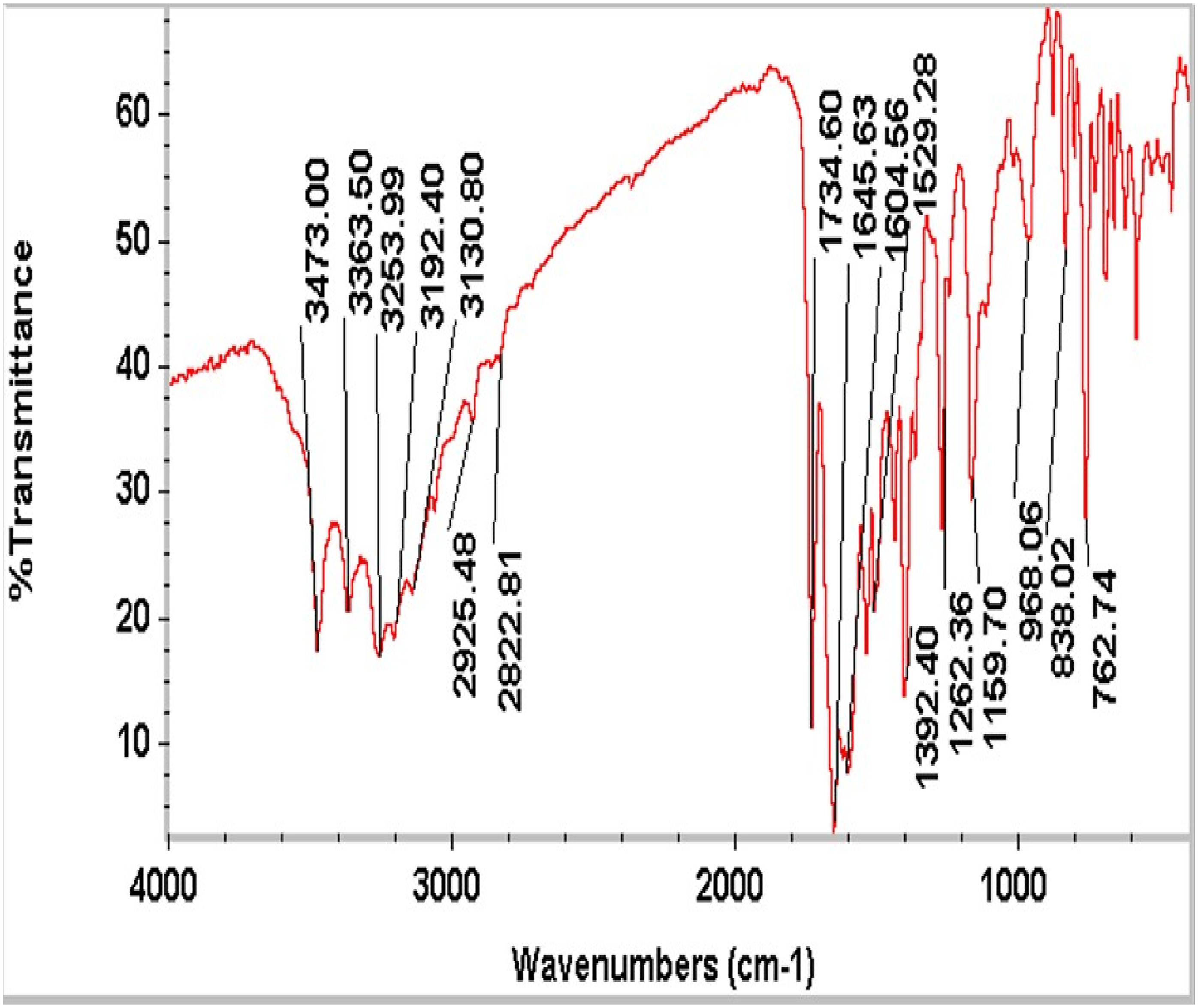

Figure S36. FT-IR spectrum of compound 10

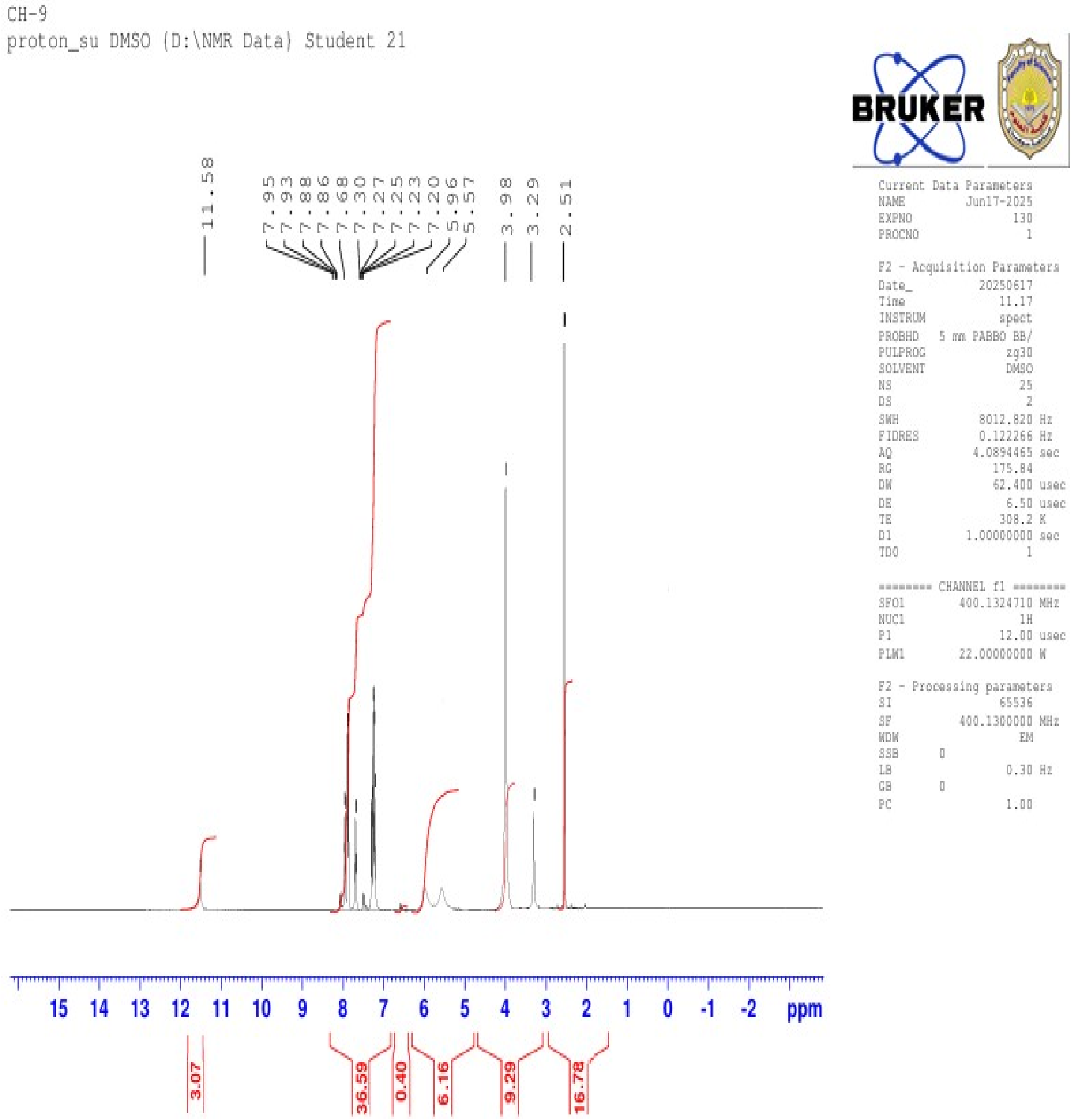

Figure S37. ^{1}HNMR spectrum of compound 10

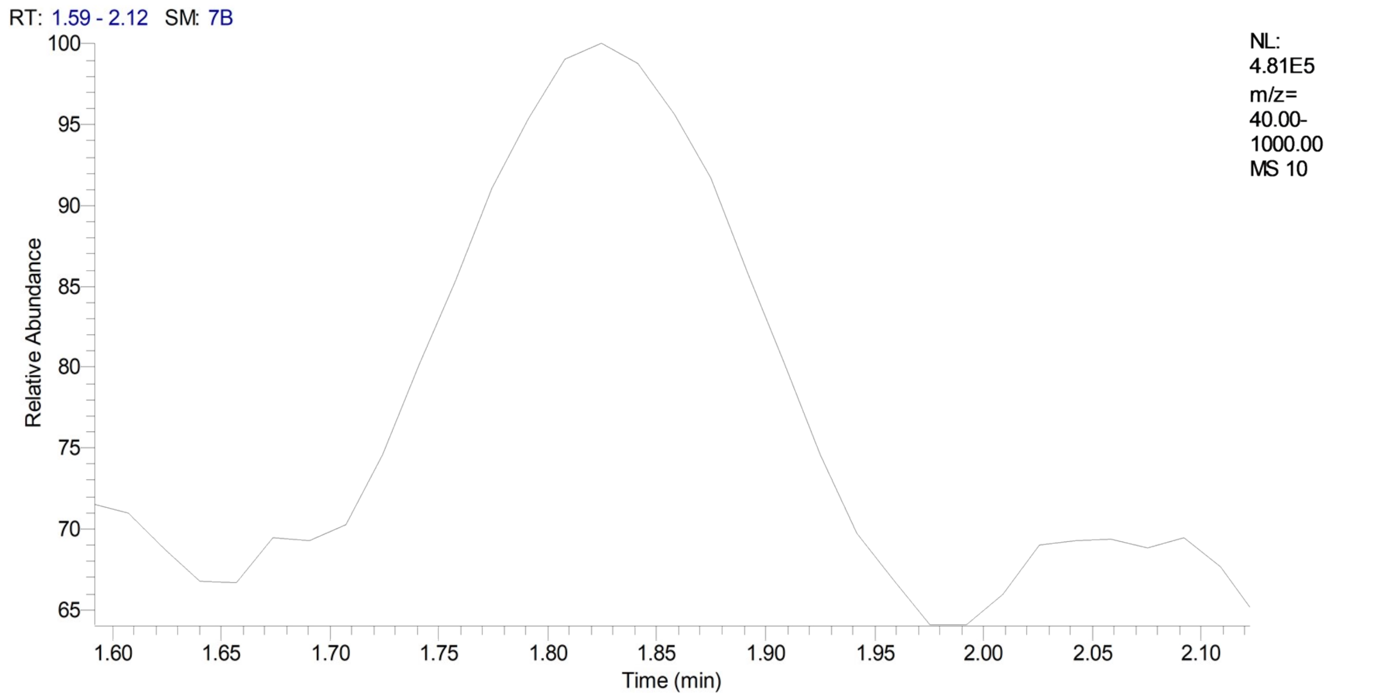
RT: 1.59 - 2.12 SM: 7B
NL:
4.81E5
m/z=
40.00-
1000.00
MS 10
Relative Abundance
Time (min)

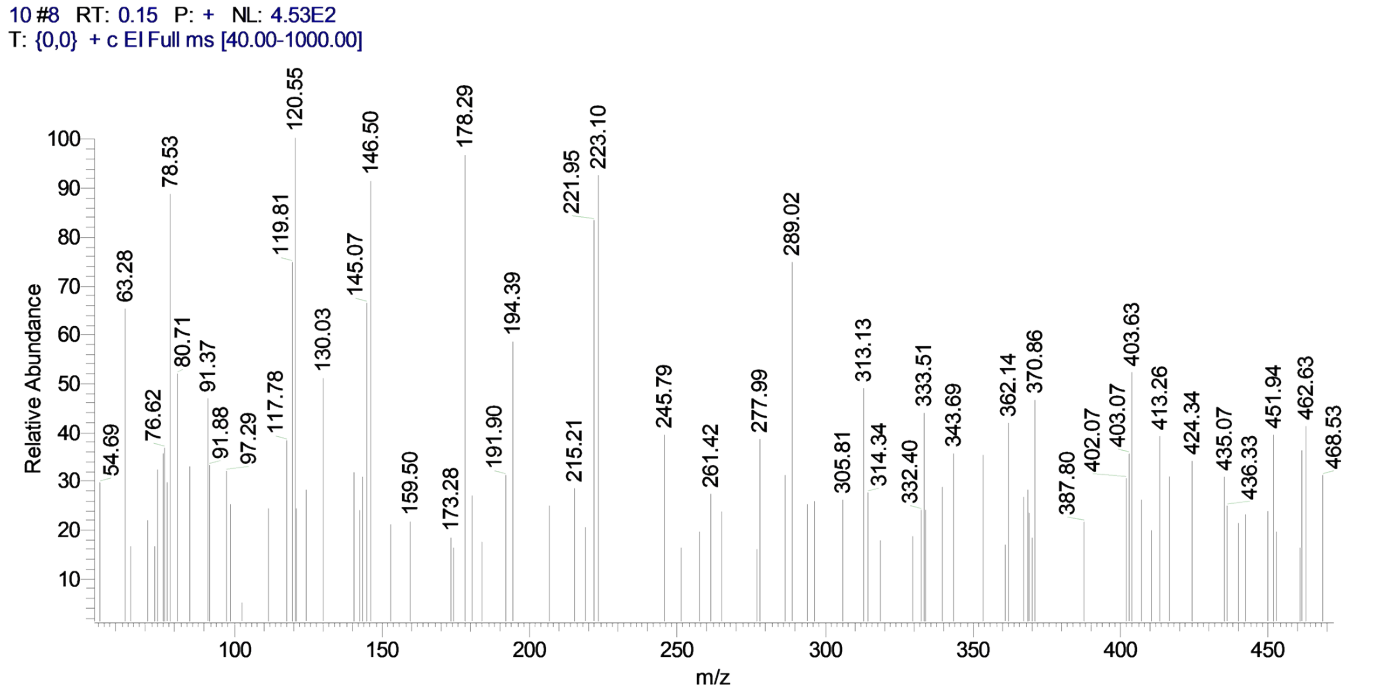
10 #8 RT: 0.15 P: + NL: 4.53E2
T: {0,0} + c EI Full ms [40.00-1000.00]
Relative Abundance
m/z
54.69
63.28
76.62
78.53
80.71
91.37
91.88
97.29
117.78
119.81
120.55
130.03
145.07
146.50
159.50
173.28
178.29
191.90
194.39
215.21
221.95
223.10
245.79
261.42
277.99
289.02
305.81
313.13
314.34
332.40
333.51
343.69
362.14
370.86
387.80
402.07
403.07
403.63
413.26
424.34
435.07
436.33
451.94
462.63
468.53

Figure S38. Mass spectrum of compound 10

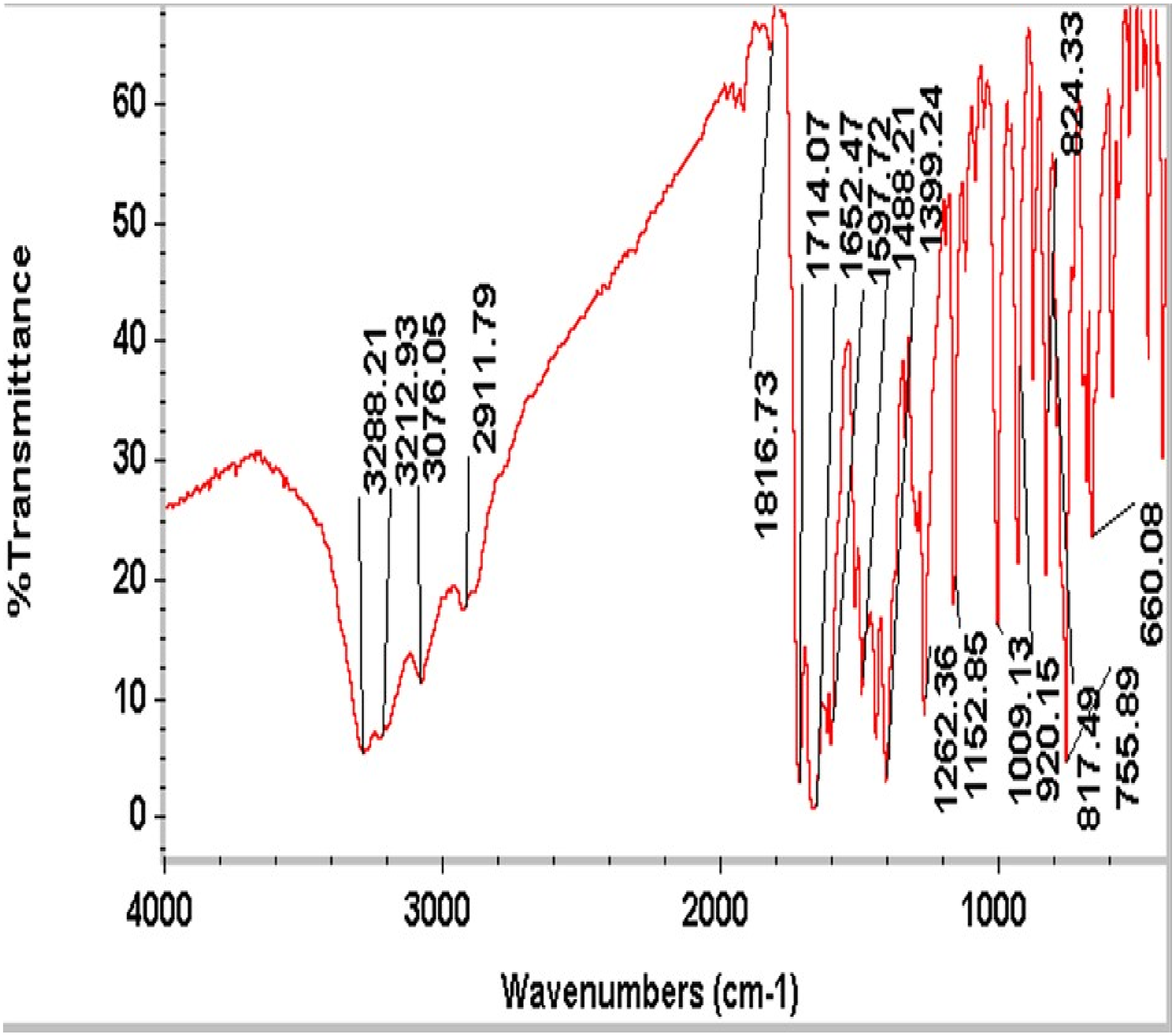

Figure S39. FT-IR spectrum of compound 11

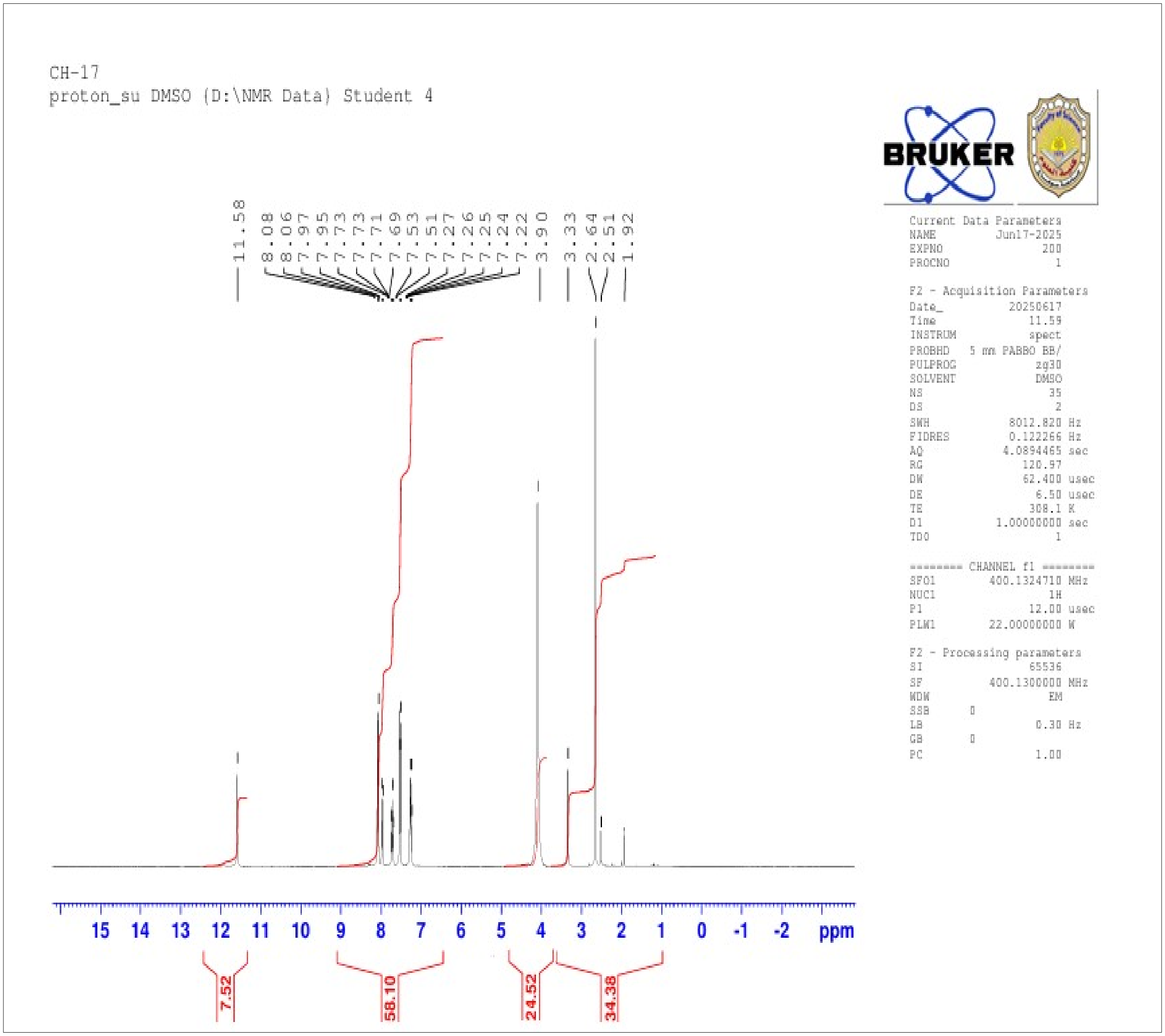

Figure S40. ^{1}HNMR spectrum of compound 11

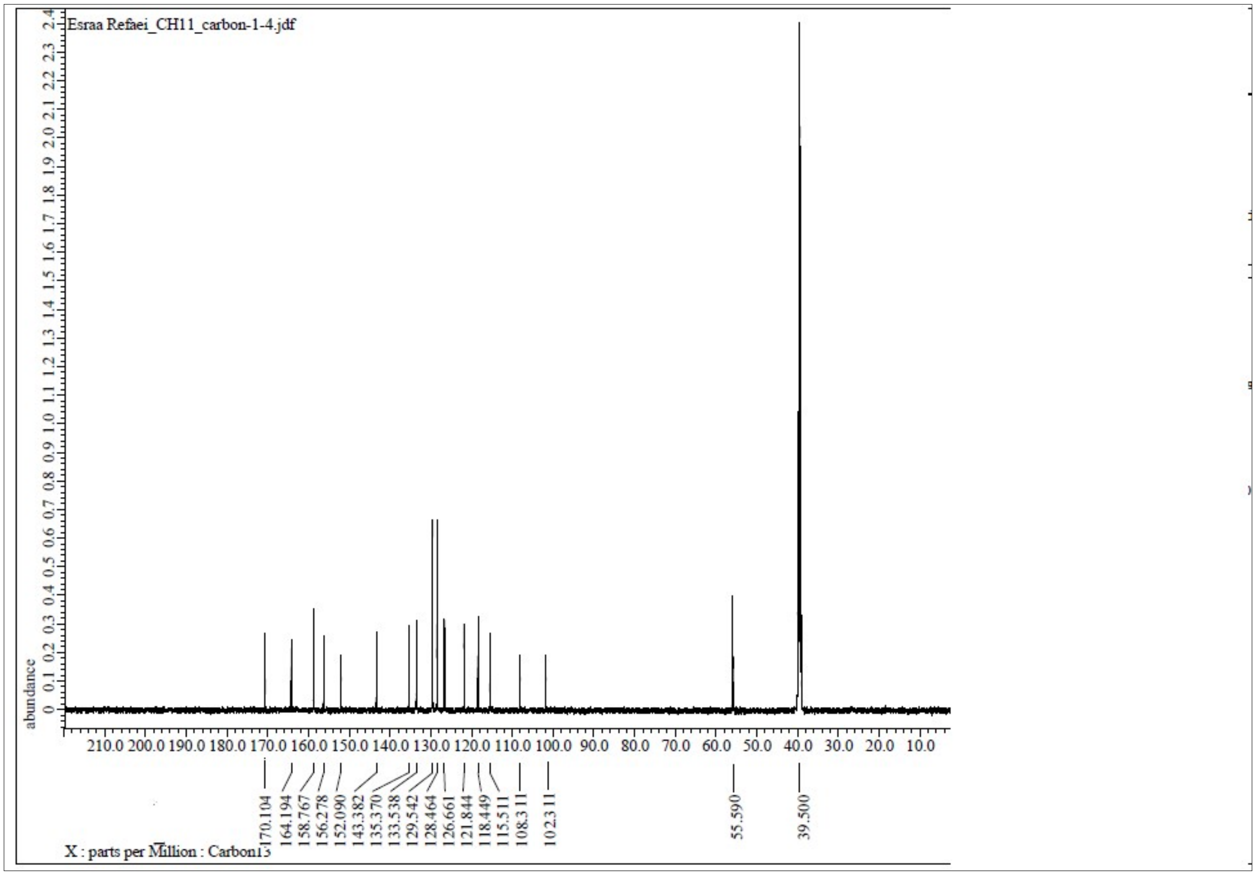

Figure S41. ^{13}CNMR spectrum of compound 11

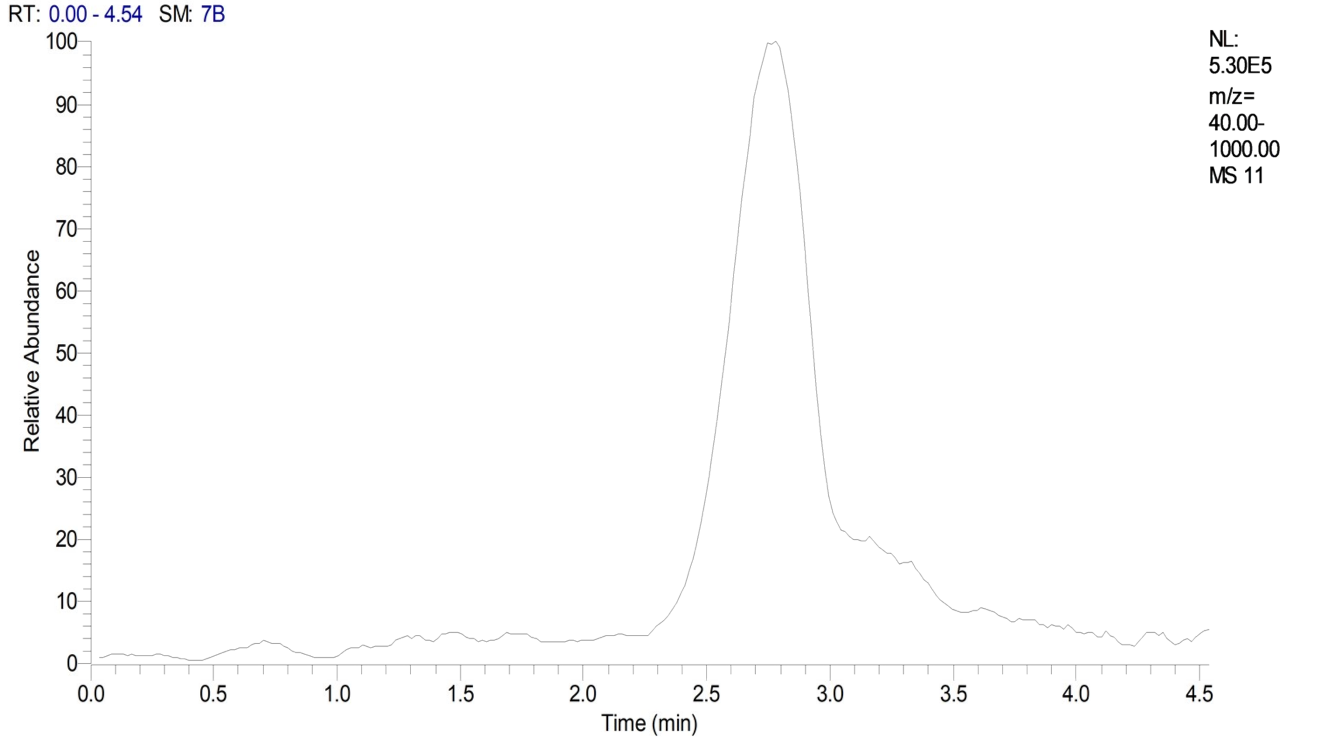
RT: 0.00 - 4.54 SM: 7B
NL:
5.30E5
m/z=
40.00-
1000.00
MS 11
Relative Abundance
Time (min)

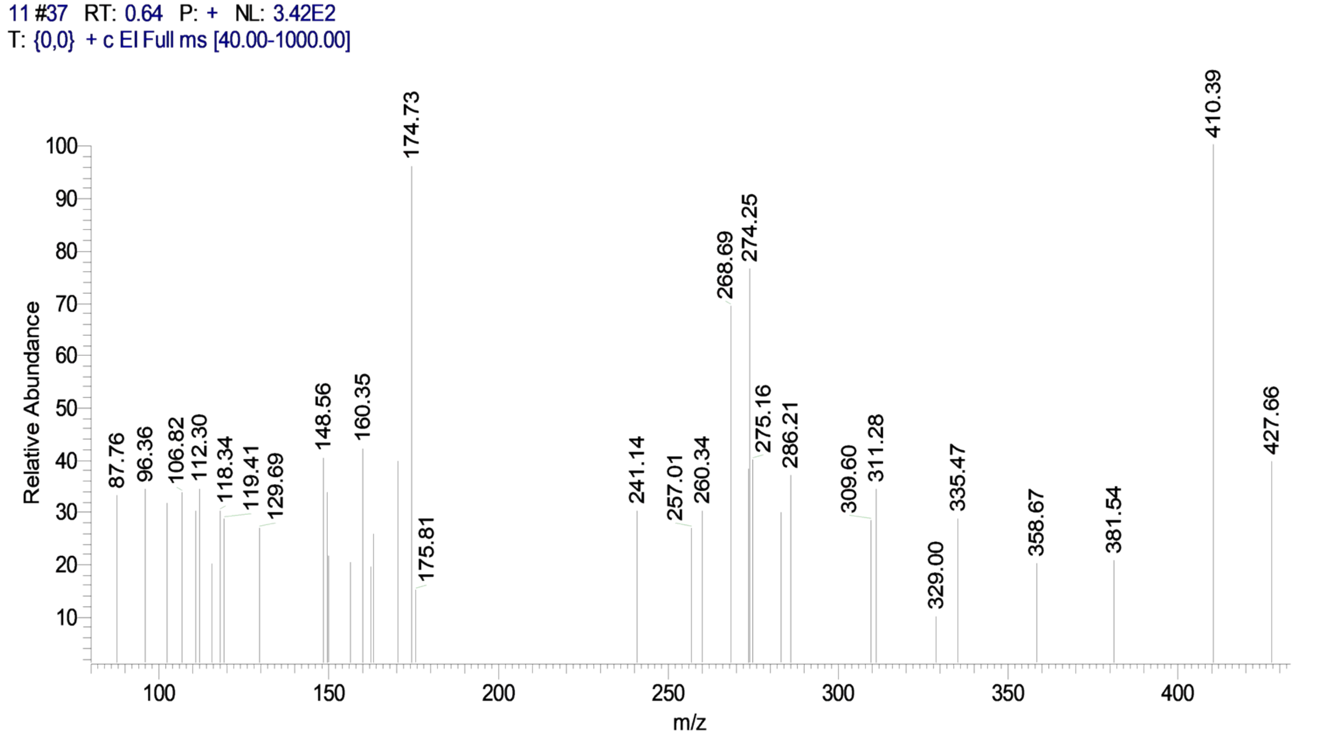
11 #37 RT: 0.64 P: + NL: 3.42E2
T: {0,0} + c EI Full ms [40.00-1000.00]
410.39
174.73
274.25
268.69
427.66
160.35
148.56
87.76
96.36
106.82
112.30
118.34
119.41
129.69
175.81
241.14
257.01
260.34
275.16
286.21
309.60
311.28
335.47
329.00
358.67
381.54
Relative Abundance
m/z

Figure S42. Mass spectrum of compound 11

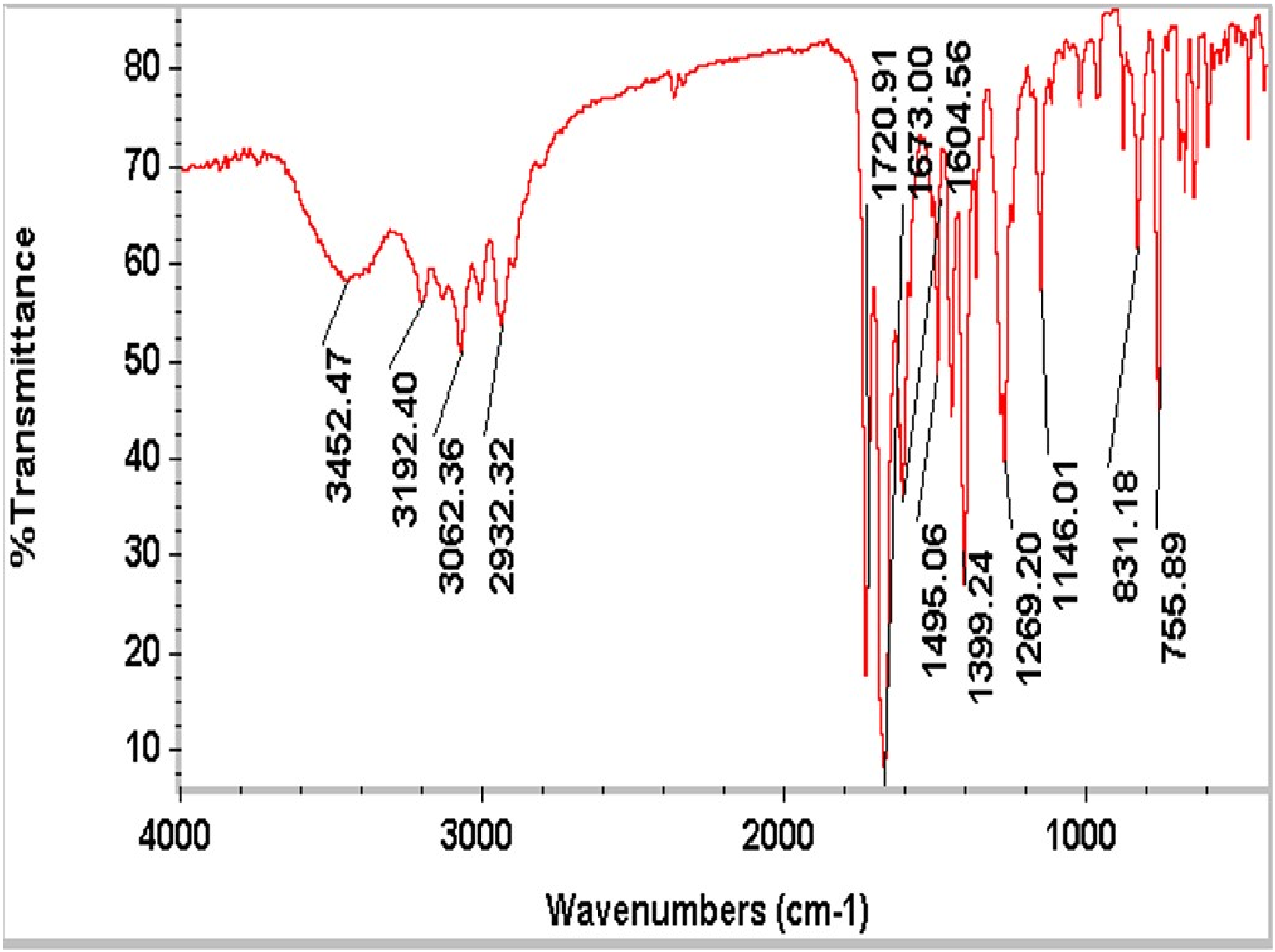

Figure S43. FT-IR spectrum of compound 12

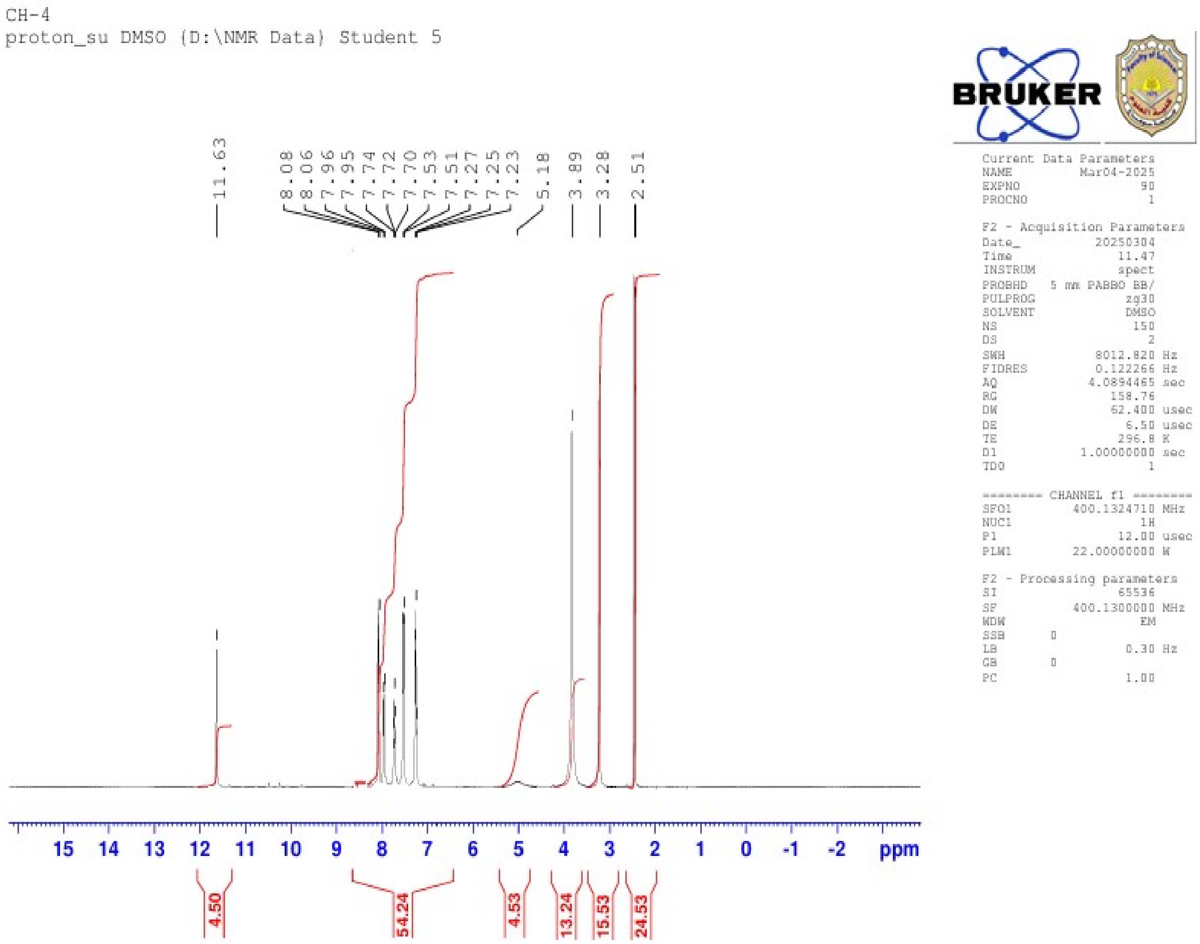

Figure S44. ^{1}HNMR spectrum of compound 12

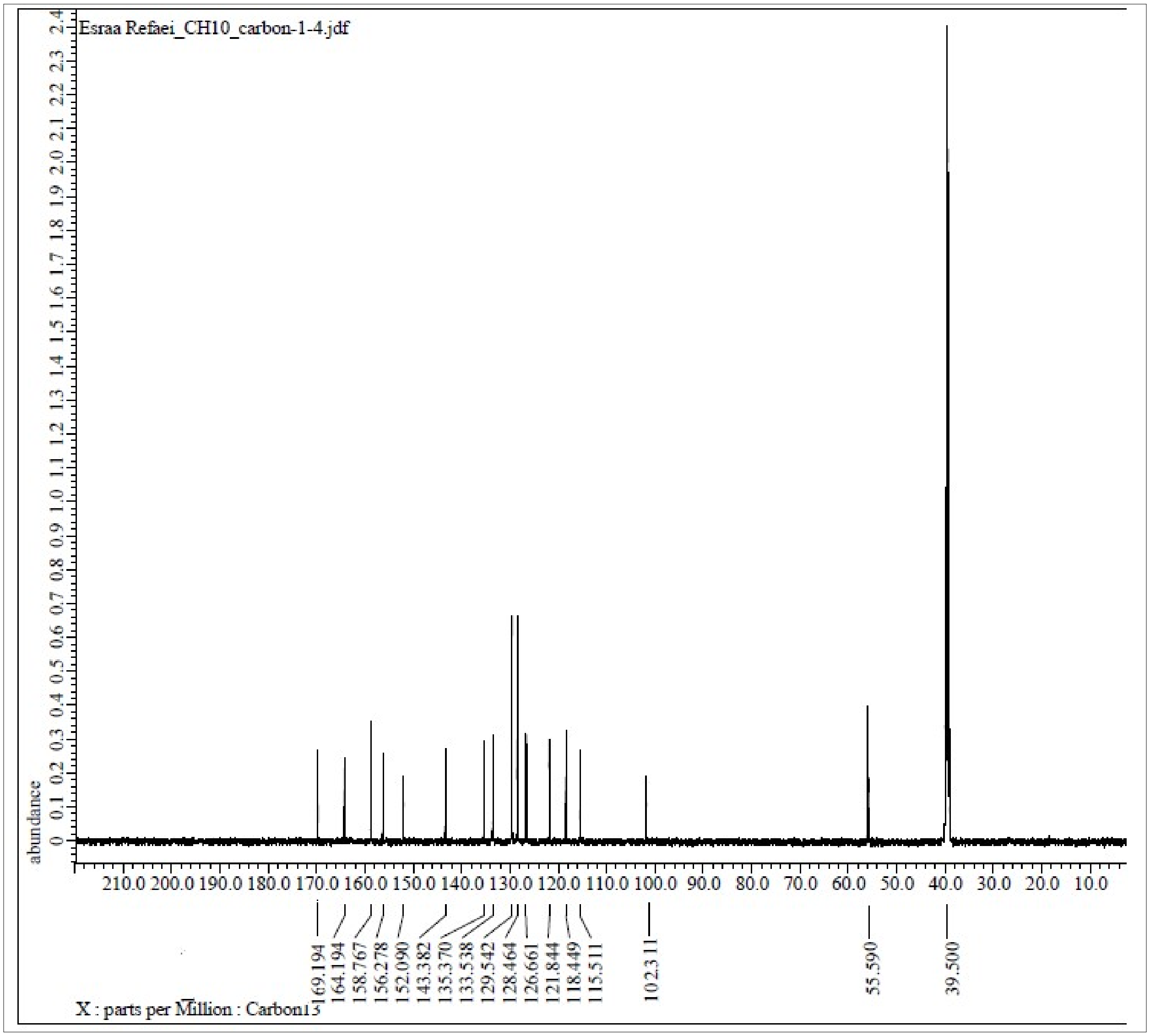

Figure S45. ^{13}CNMR spectrum of compound 12

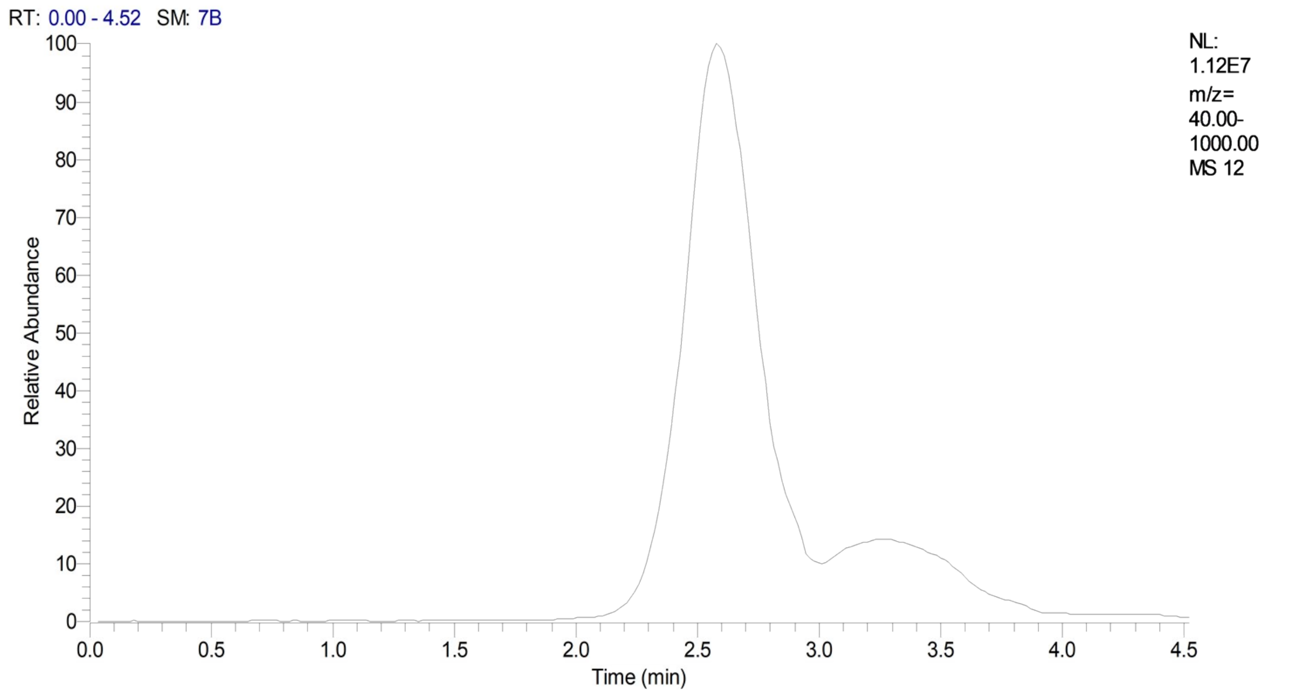

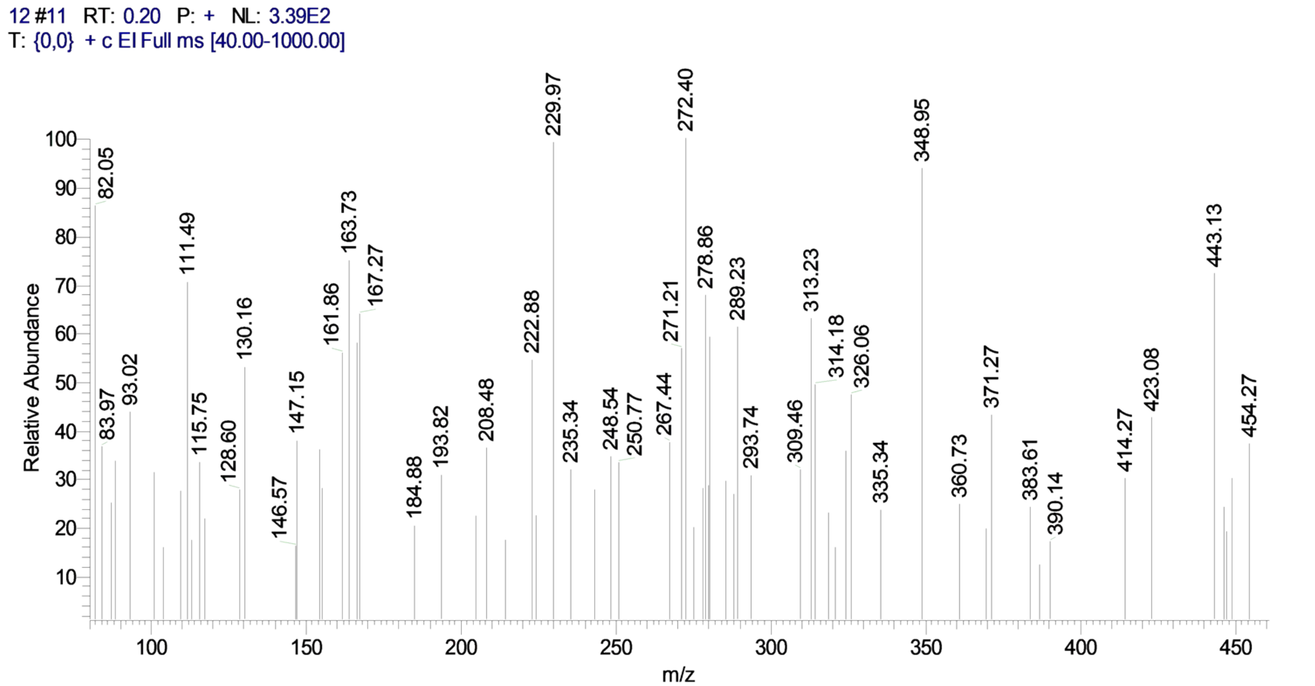

Figure S46. Mass spectrum of compound 12

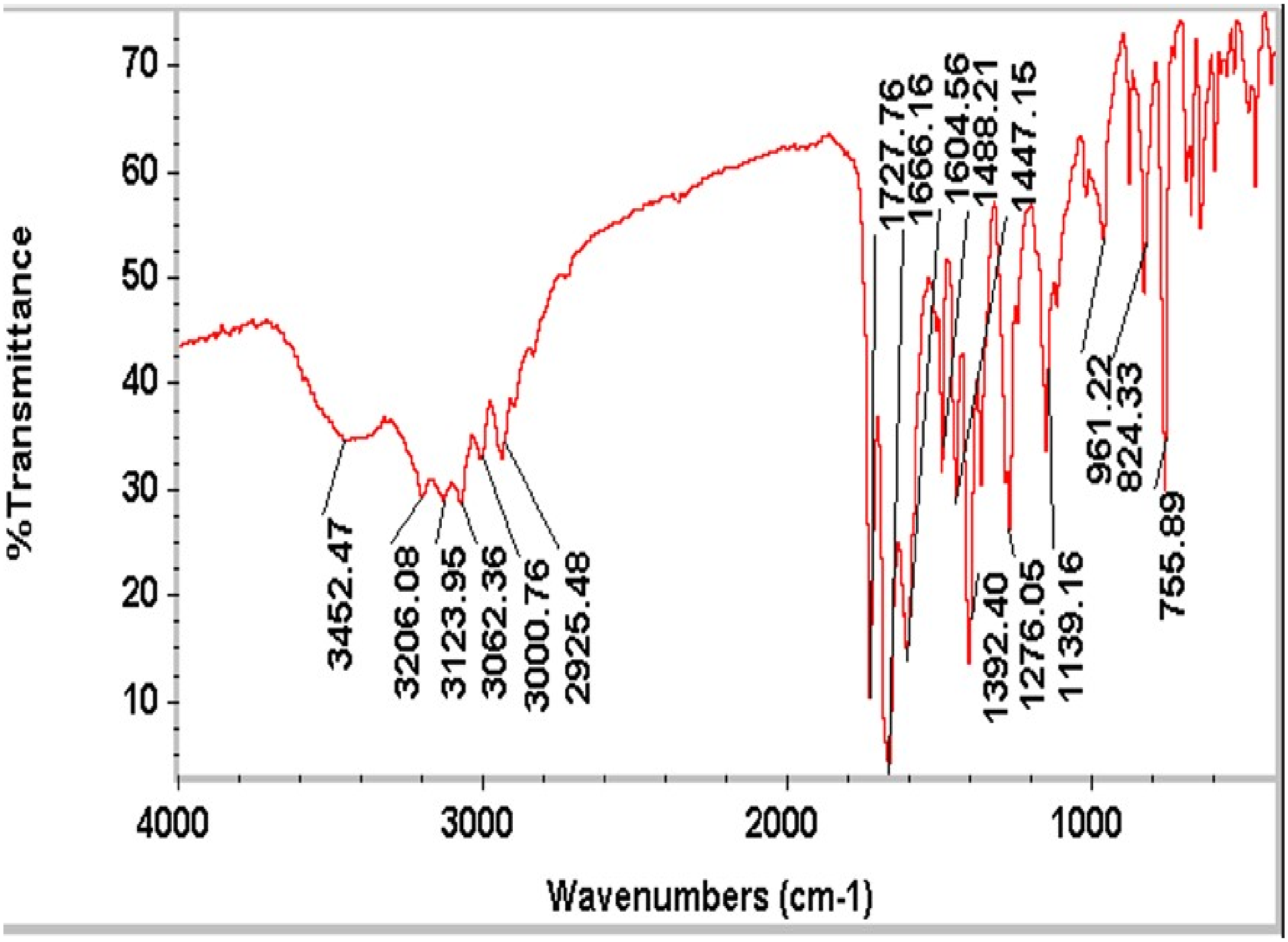

Figure S47.FT- IR spectrum of compound 13

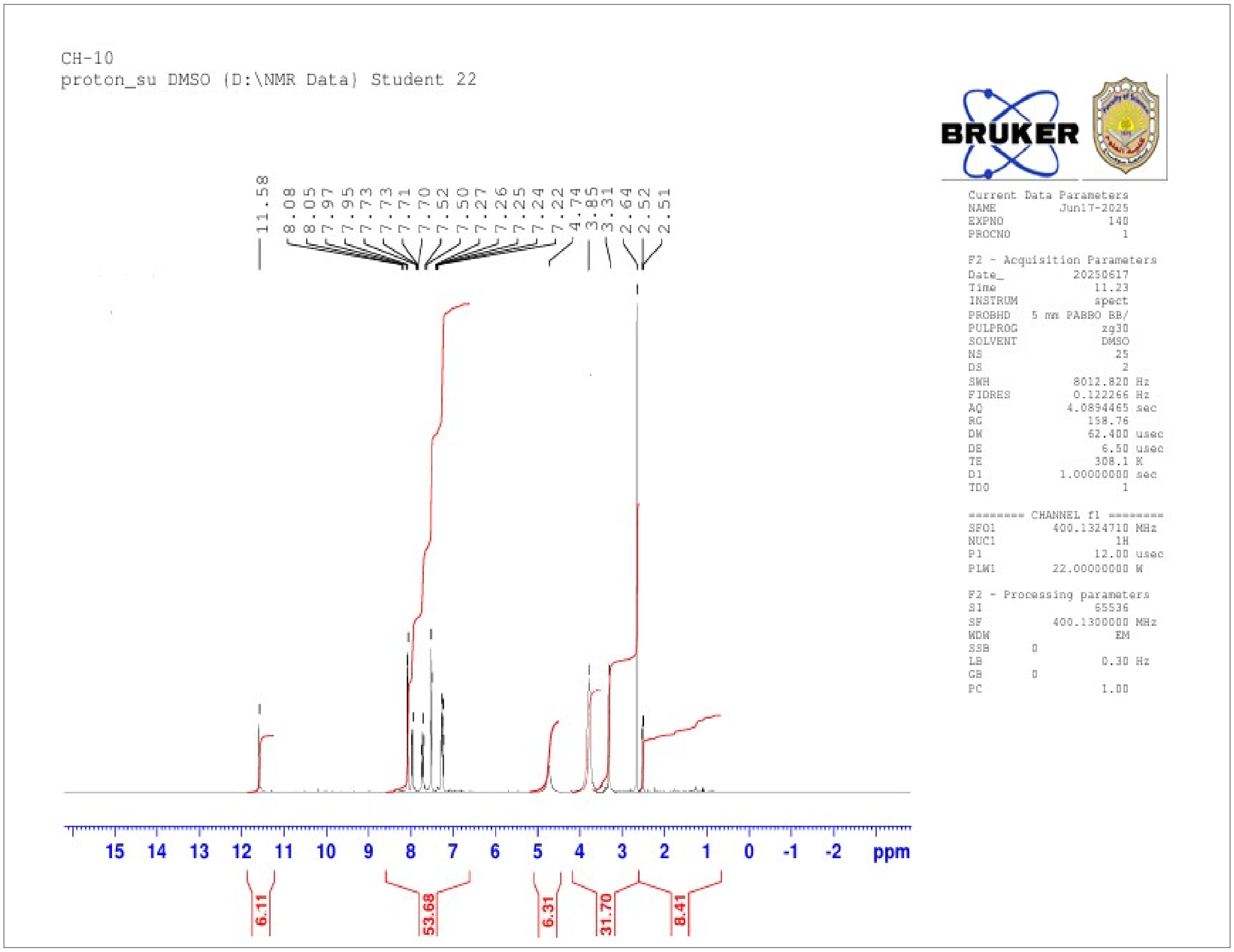

Figure S48. ^{1}HNMR spectrum of compound 13

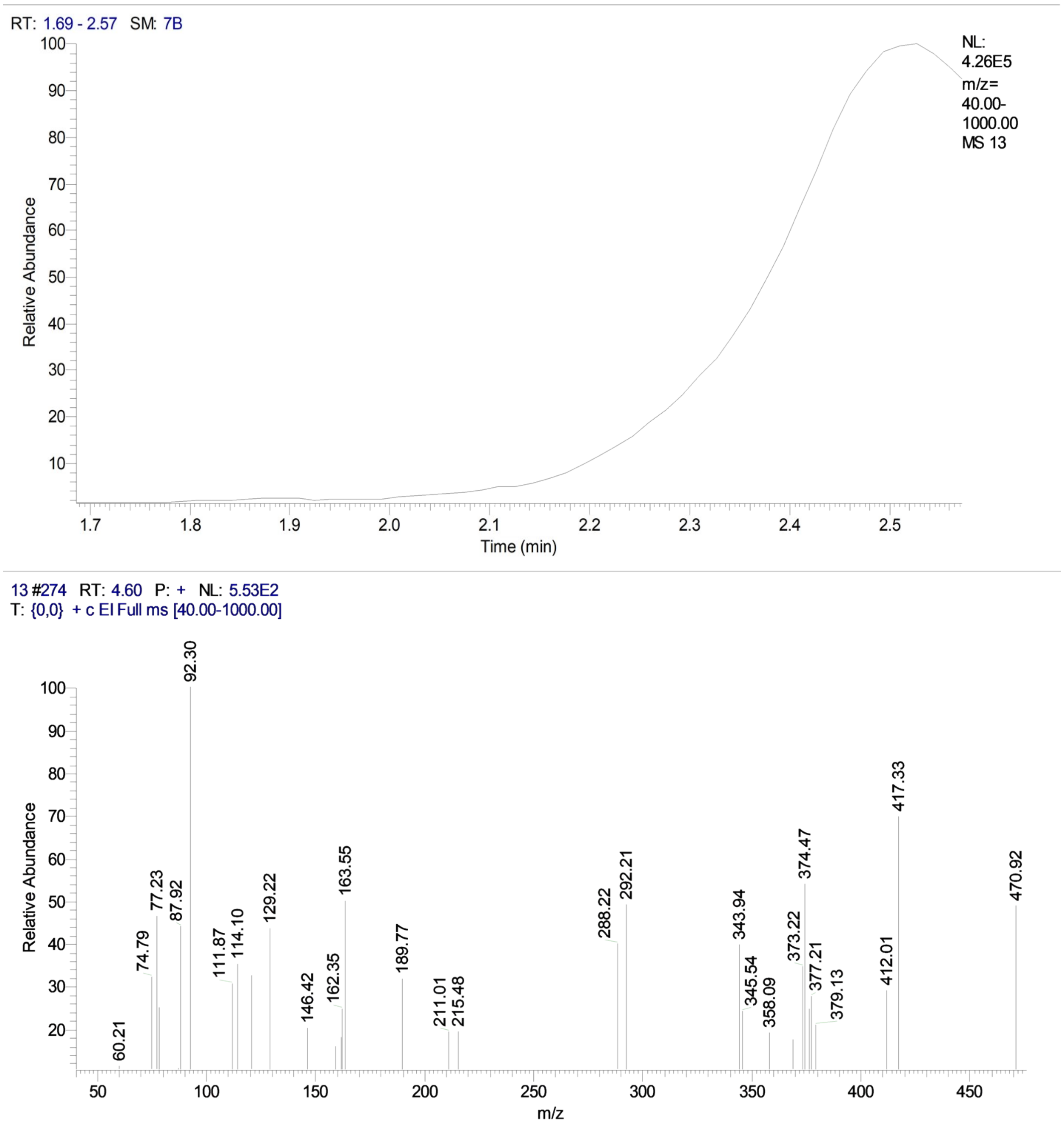

Figure S49. Mass spectrum of compound 13

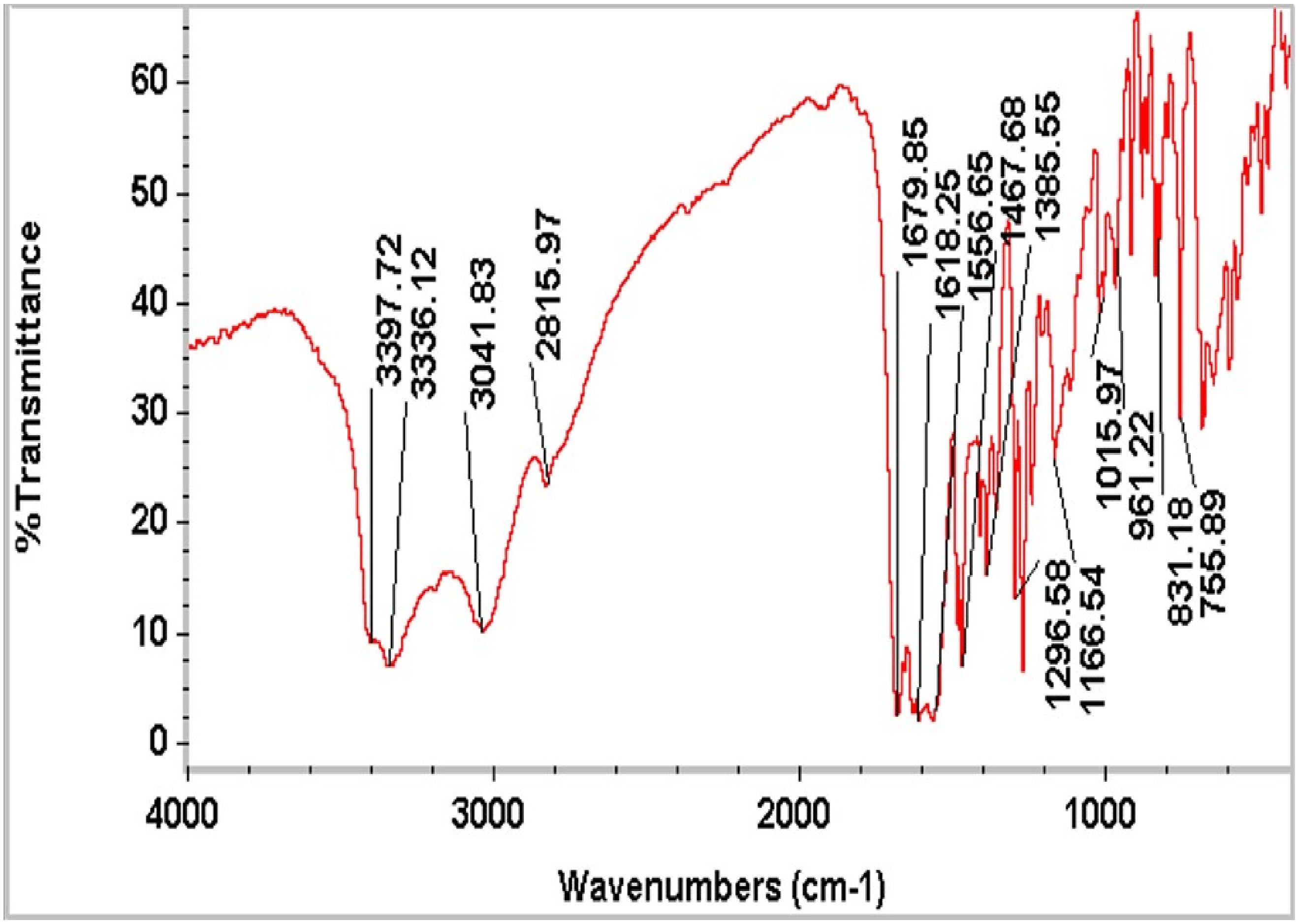

Figure S50. FT-IR spectrum of compound 14

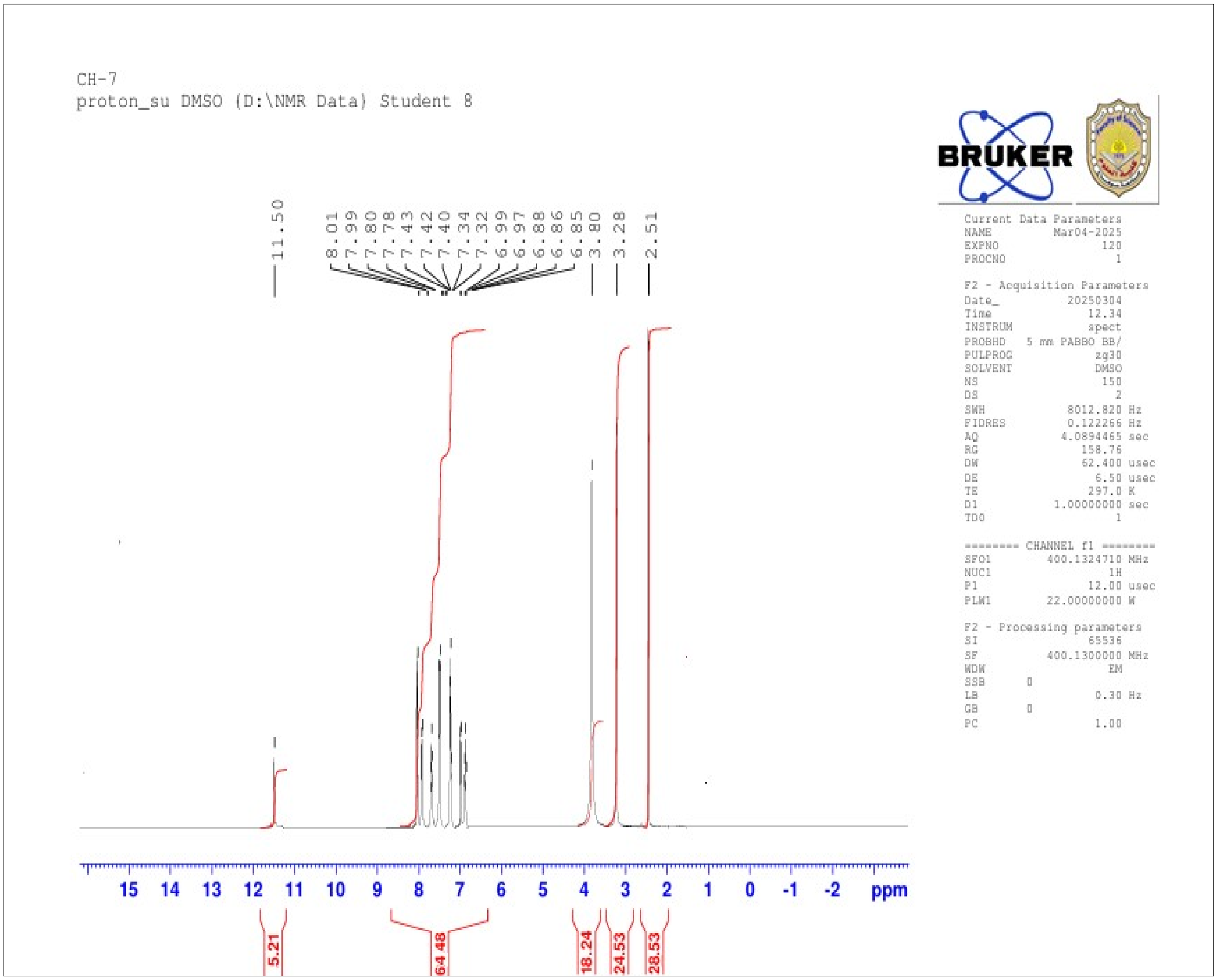

Figure S51. 1HNMR spectrum of compound 14

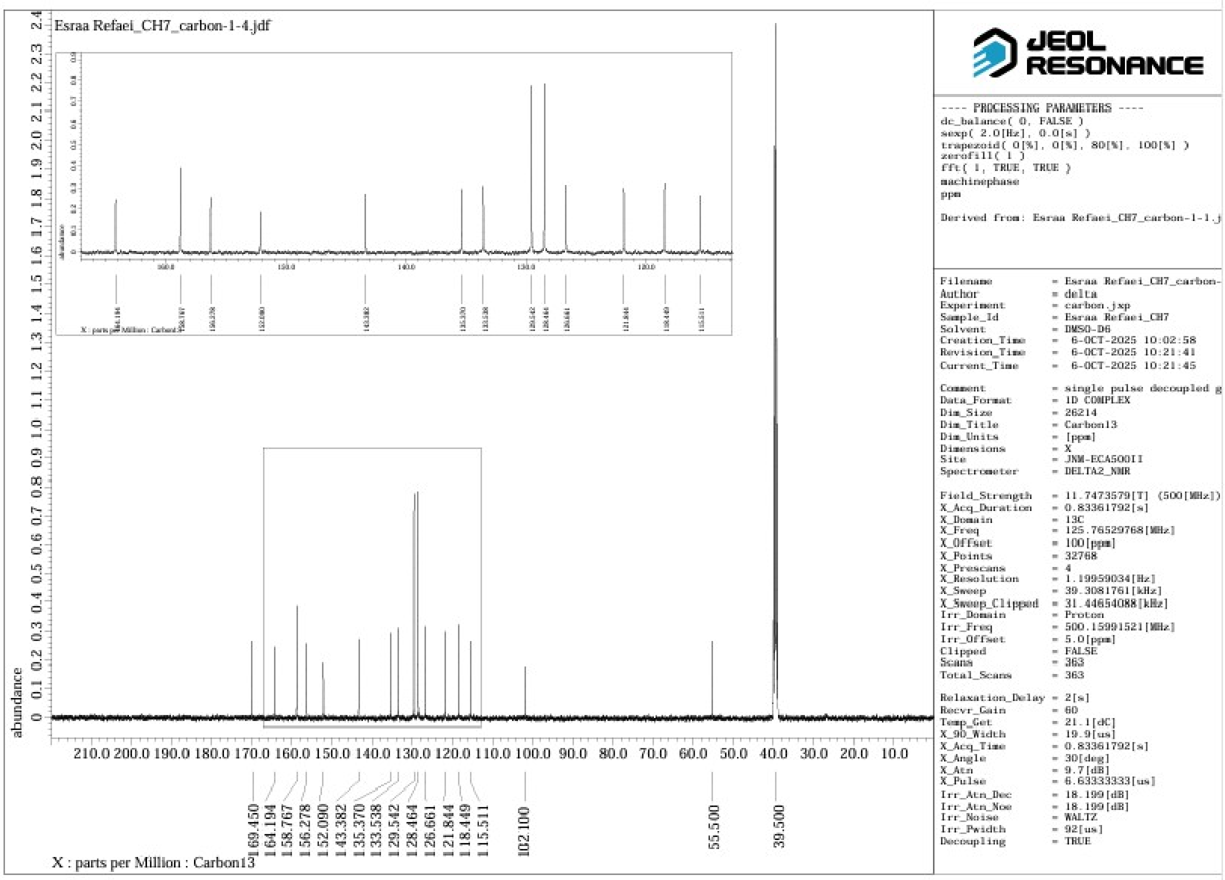

Figure S52. ^{13}CNMR spectrum of compound 14

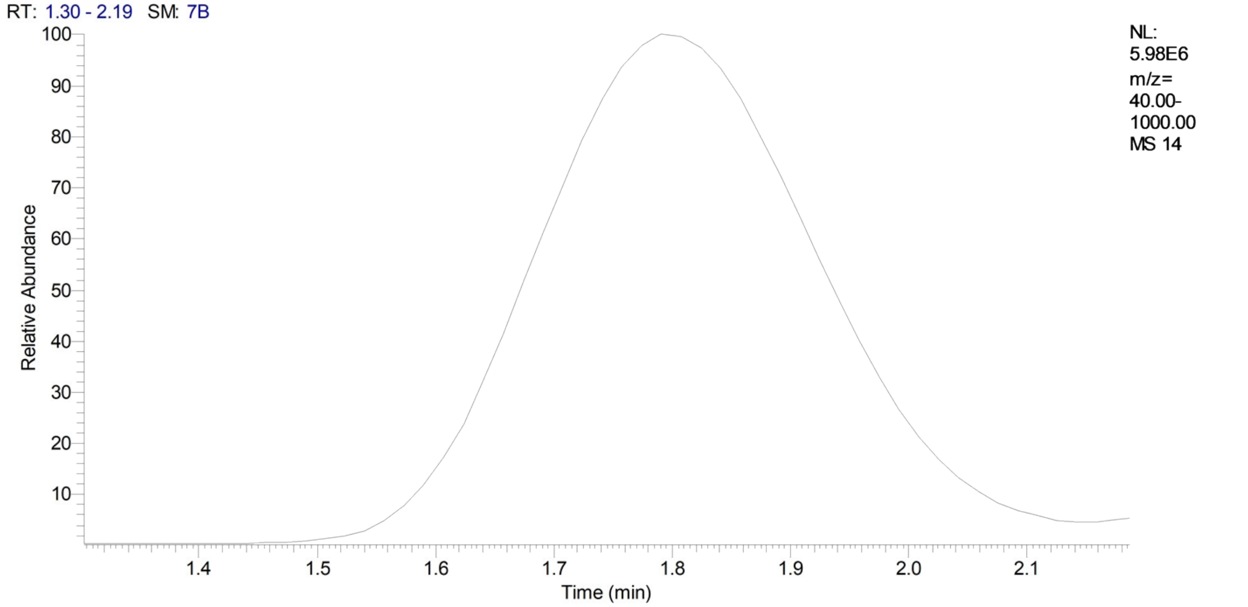

RT: 1.30 - 2.19 SM: 7B
NL: 5.98E6
m/z= 40.00-1000.00
MS 14
Relative Abundance
Time (min)

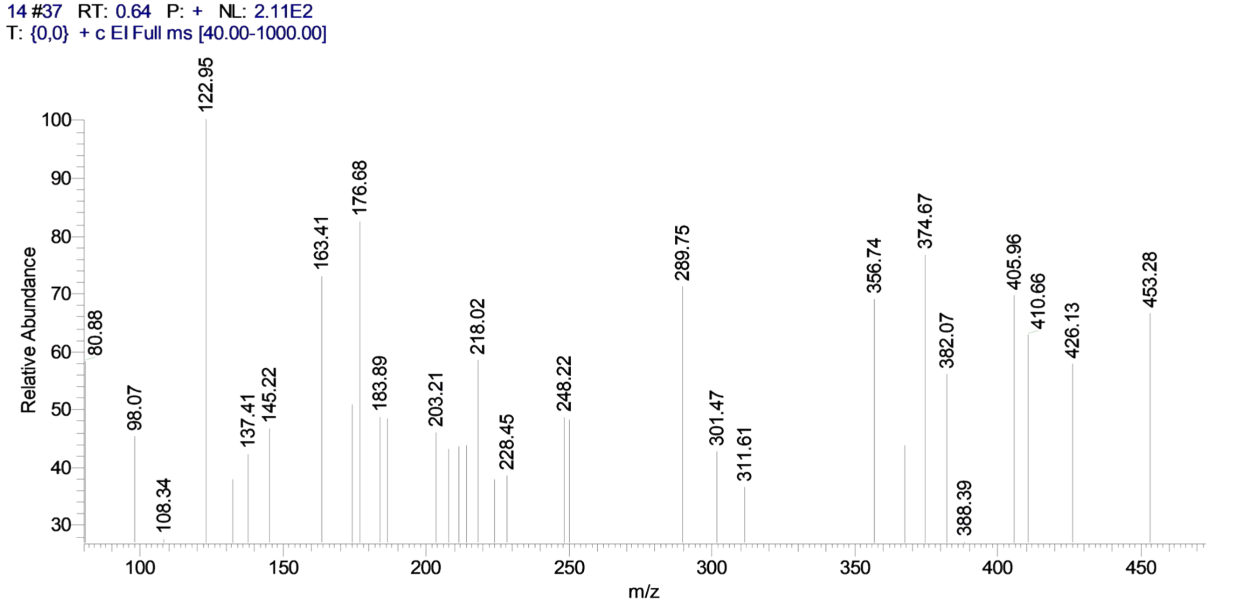

14 #37 RT: 0.64 P: + NL: 2.11E2
T: {0,0} + c EI Full ms [40.00-1000.00]
122.95
176.68
374.67
163.41
289.75
405.96
356.74
453.28
410.66
80.88
218.02
426.13
382.07
98.07
137.41
145.22
183.89
203.21
248.22
228.45
301.47
311.61
108.34
388.39
Relative Abundance
m/z

Figure S53. Mass spectrum of compound 14

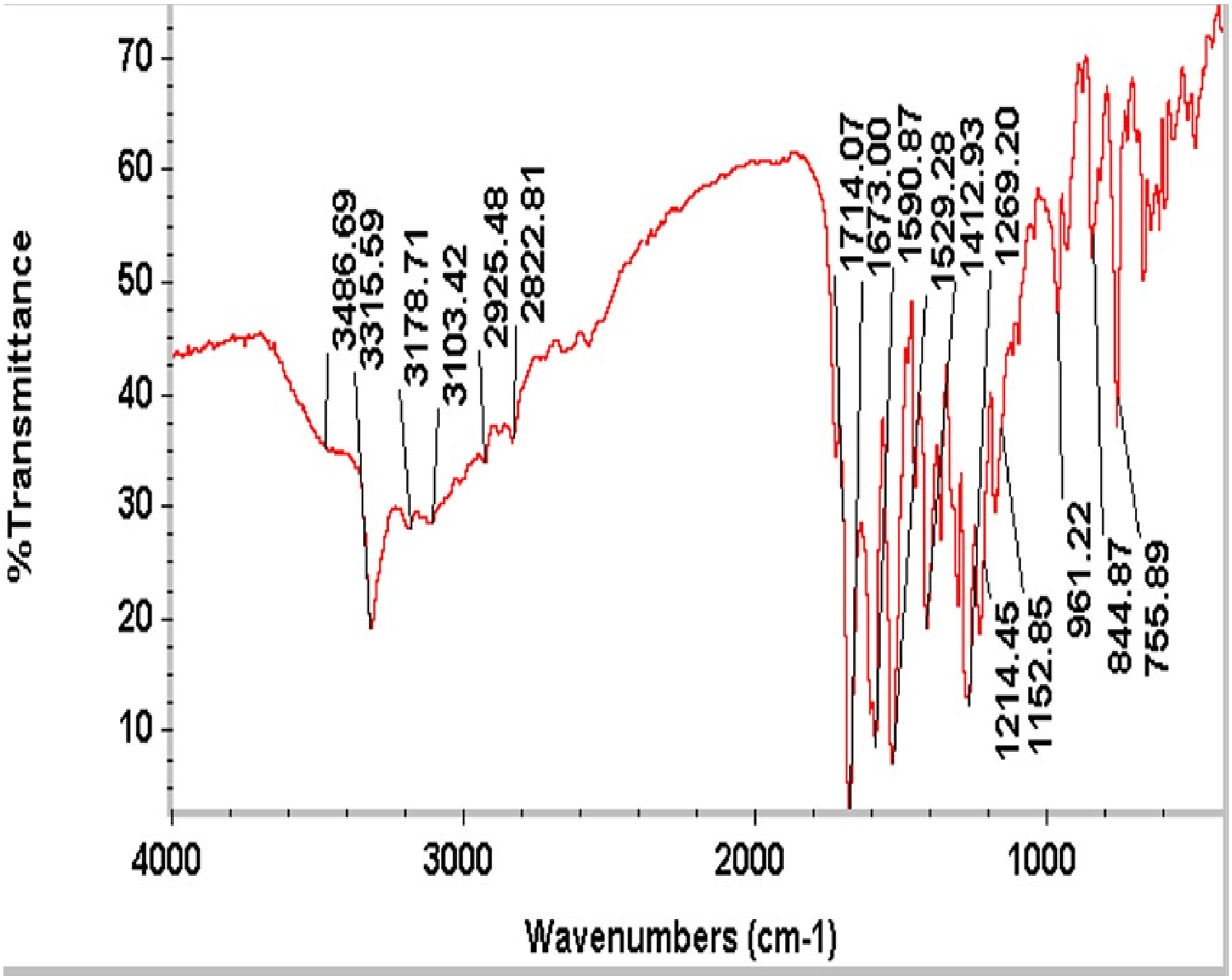

Figure S54.FT- IR spectrum of compound 15

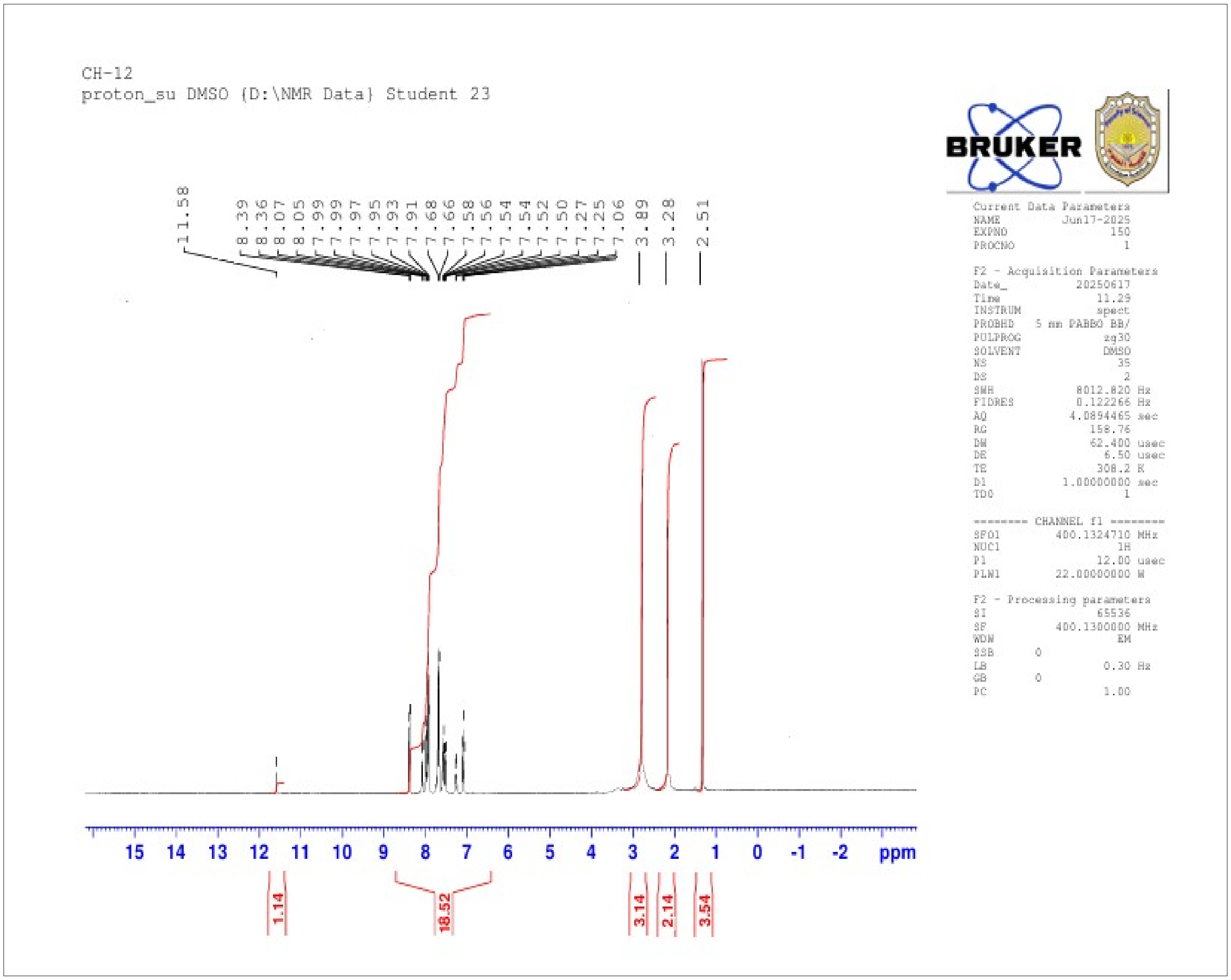

Figure S55. ^{1}HNMR spectrum of compound 15

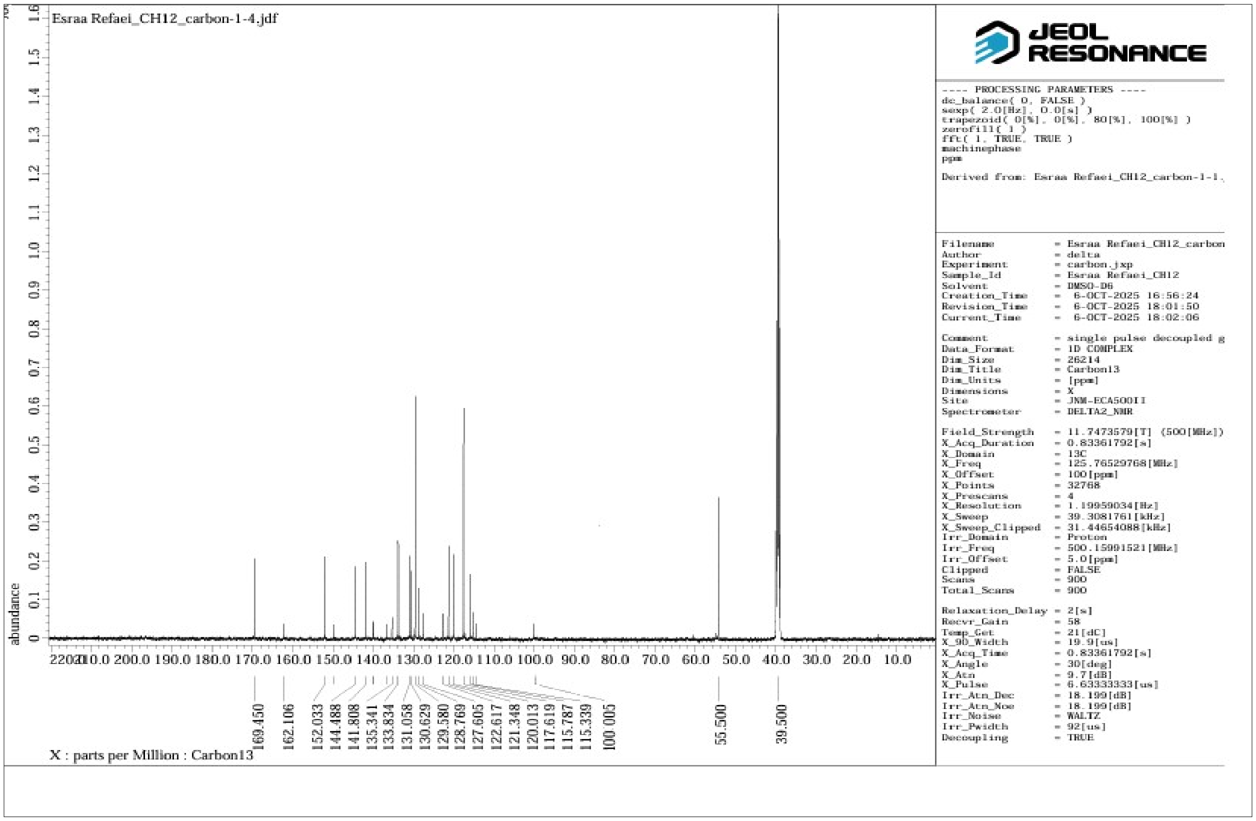

Figure S56. ^{13}CNMR spectrum of compound 15

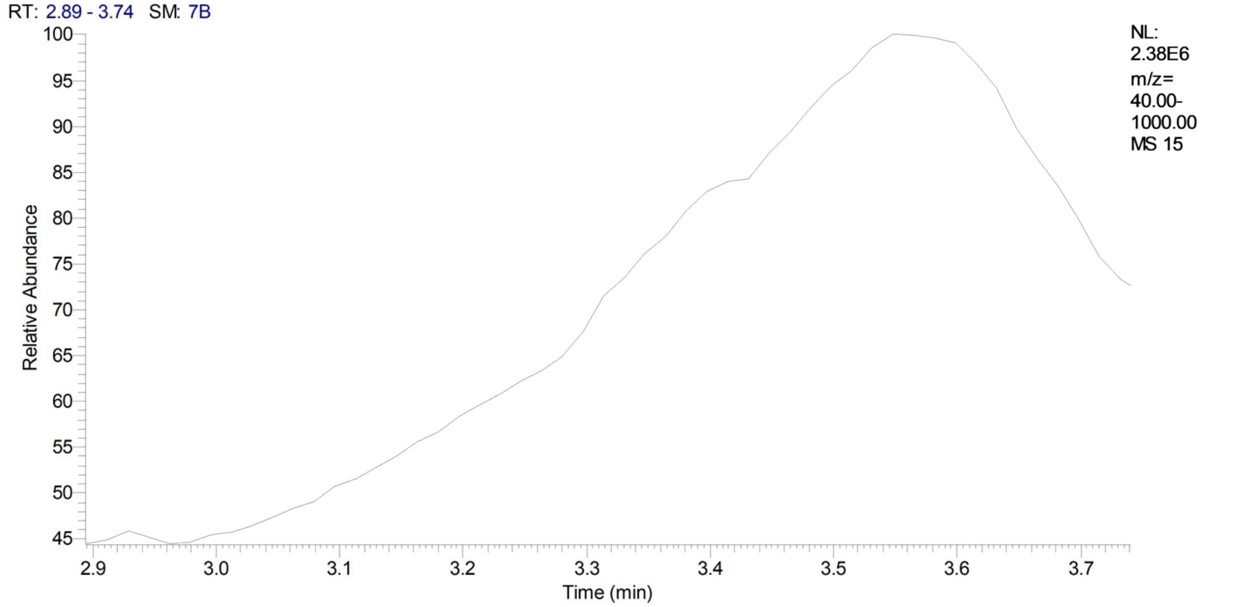
RT: 2.89 - 3.74 SM: 7B
NL: 2.38E6 m/z= 40.00-1000.00 MS 15
Relative Abundance
Time (min)

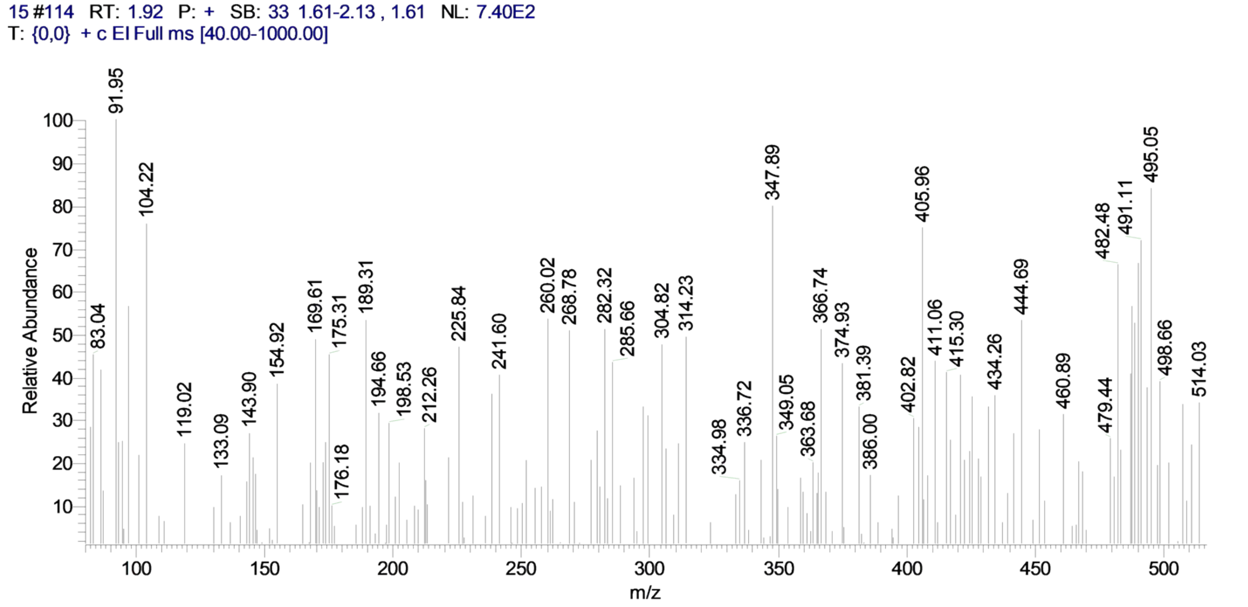
15 #114 RT: 1.92 P: + SB: 33 1.61-2.13 , 1.61 NL: 7.40E2
T: {0,0} + c EI Full ms [40.00-1000.00]
91.95
104.22
83.04
119.02
133.09
143.90
154.92
169.61
175.31
176.18
189.31
194.66
198.53
212.26
225.84
241.60
260.02
268.78
282.32
285.66
304.82
314.23
334.98
336.72
347.89
349.05
363.68
366.74
374.93
381.39
386.00
402.82
405.96
411.06
415.30
434.26
444.69
460.89
479.44
482.48
491.11
495.05
498.66
514.03
Relative Abundance
m/z

Figure S57. Mass spectrum of compound 15
